# Causal variants in Maturity Onset Diabetes of the Young (MODY) – A systematic review

**DOI:** 10.1186/s12902-021-00891-7

**Published:** 2021-11-11

**Authors:** Ibrar Rafique, Asif Mir, Muhammad Arif Nadeem Saqib, Muhammad Naeem, Luc Marchand, Constantin Polychronakos

**Affiliations:** 1 Department of Biological Sciences, International Islamic University, Islamabad, Pakistan; 2 Graduate Research Trainee, Department of Pediatrics and Human Genetics, McGill University Health Centre Research Institute, Montreal, Canada; 3 Research Officer, Pakistan Health Research Council, Sector G-5/2, Islamabad, Pakistan; 4 Department of Medical Laboratory Technology, National Skills University, Islamabad, Pakistan; 5 Department of Biotechnology, Quaid-i-Azam University, Islamabad, Pakistan; 6 Department of Pediatrics and Human Genetics, McGill University Health Centre Research Institute, Montreal, Canada; 7 Departments of Pediatrics and Human Genetics, McGill University Health Centre Research Institute, 1001 Decarie Boulevard, Montréal, Québec Canada

**Keywords:** Causal variants, MODY, Diabetes, Genetics

## Abstract

**Background:**

Maturity Onset Diabetes of the Young (MODY) is an autosomal dominant type of diabetes. Pathogenic variants in fourteen genes are reported as causes of MODY. Its symptoms overlap with type 1 and type 2 diabetes. Reviews for clinical characteristics, diagnosis and treatments are available but a comprehensive list of genetic variants, is lacking. Therefore this study was designed to collect all the causal variants involved in MODY, reported to date.

**Methods:**

We searched PubMed from its date of inception to December 2019. The search terms we used included disease names and name of all the known genes involved. The ClinVar database was also searched for causal variants in the known 14 MODY genes.

**Results:**

The record revealed 1647 studies and among them, 326 studies were accessed for full-text. Finally, 239 studies were included, as per our inclusion criteria. A total of 1017 variants were identified through literature review and 74 unpublished variants from Clinvar database. The gene most commonly affected was *GCK*, followed by *HNF1a*. The traditional Sanger sequencing was used in 76 % of the cases and 65 % of the studies were conducted in last 10 years. Variants from countries like Jordan, Oman and Tunisia reported that the MODY types prevalent worldwide were not common in their countries.

**Conclusions:**

We expect that this paper will help clinicians interpret MODY genetics results with greater confidence. Discrepancies in certain middle-eastern countries need to be investigated as other genes or factors, like consanguinity may be involved in developing diabetes.

**Supplementary Information:**

The online version contains supplementary material available at 10.1186/s12902-021-00891-7.

## Background

Maturity Onset diabetes of the Young (# 606,392) is an autosomal dominant genetic disease. Its prevalence is 1-5 % of all type of diabetes [1]. It is usually difficult to diagnose as most of the clinical symptoms overlap with type 1 and type 2 diabetes. A number of methods are available to assess the probability of having MODY and clinical tests like C-peptide, autoantibody testing can help distinguish type 1 diabetes from MODY [2]. The features that differentiate MODY from type 2 diabetes are lean body mass and early age at onset of the diabetes. The diagnosis of MODY is crucial as it has therapeutic implications.*GCK* MODY does not required any treatment and is also not associated with any complications and for *HNF1a* MODY3, sulphonylurea is used as first line antidiabetic treatment [3]. The genes mutated in MODY are listed in OMIM (# 606,391). MODY2 (*GCK*) and MODY3 (*HNF1a*) are most commonly reported [4].

The traditional method used for the identification of MODY was using direct sequencing of most commonly affected genes in MODY like *GCK, HNF1a* and *HNF4a*. But now with the advent of latest technology, most of the studies have been using targeted next generation sequencing or whole exome sequencing. Τargeted NGS, uses a panel of genes that are reported to be involved in MODY. The whole exome sequencing is advantageous as it helped in identification of new genes involved in MODY [5].

There are many reviews available for clinical symptoms and types of MODY but there was lack of reviews on genetic variants involved in MODY. The objective of this review was to collate the genetic variants reported so far from the literature on the known 14 genes involved in MODY, as listed in OMIM.

## Methods

### Search strategy

A systematic review was conducted following the PRISMA guidelines. The PubMed data base was used for the review. The literature was searched and articles were included until 01 December 2019. Different combinations of search terms were used for search strategy, as follows: Maturity Onset diabetes of the Young OR Monogenic diabetes AND MODY OR *ABCC8* OR *APPL1* OR *BLK* OR *CEL* OR *GCK* OR *HNF1A* OR *HNF1B* OR *HNF4A* OR *INS* OR *KCNJ11* OR *KLF11* OR *NEUROD1* OR *PAX4* OR *PDX1*. The search was conducted by one of the authors (IR) and the initial screening was done by reviewing the titles and abstracts by two authors (IR and CP). Those found relevant were accessed for fulltext. These articles were then reviewed as per the inclusion criteria.

### Study selection

**Fig. 1:**
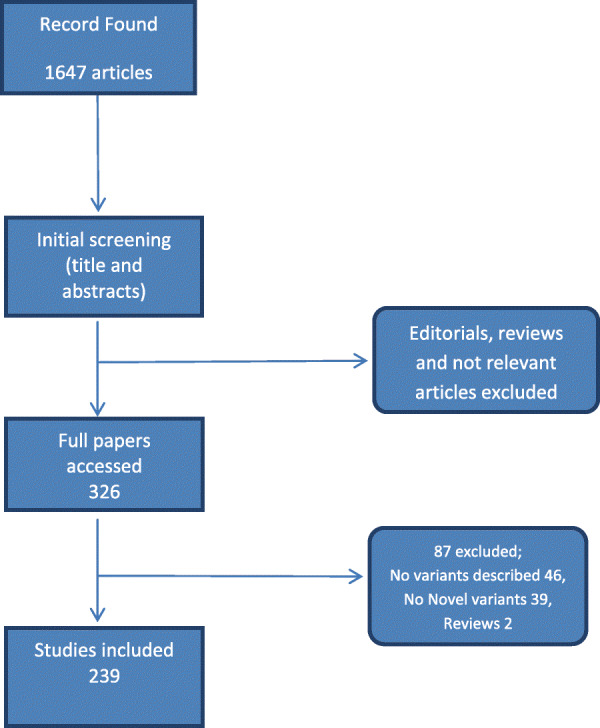
PRISMA flow diagram showing the steps for a systematic review of the literature

The studies were selected on the basis of following criteria (i) the study identified MODY cases i.e. which was young onset, non-autoimmune, non-insulin resistance non-neonatal and non-syndromic (ii) identified a novel pathogenic variant (iii) published in English Language (studies published in other language but with an abstract in English, and describing novel variants were also included in analysis). The studies from all geographical locations were included, irrespective of year of publication. The exclusion criteria were reviews, commentaries and already reported variants (Figure 1).

### Data extraction

The studies fulfilling the inclusion criteria were reviewed and information from these studies was tabulated. Data including author name, country of origin, publication year, variant, gene involved and methodology used to identify the variant was taken. Only those novel variants which were pathogenic, likely pathogenic or uncertain significance and having impact on protein structure (software prediction) as reported by the authors were included. The benign, likely benign were excluded. The ClinVar database of NCBI (https://www.ncbi.nlm.nih.gov/clinvar/) was also searched for variants in the 14 known genes involved in MODY, which were also included even if not reported in the literature. Investigators reviewed the extracted data from eligible publications independently from each other following inclusion and exclusion criteria as mentioned.

The variants were validated by using variant validator (https://variantvalidator.org/) and accession numbers were provided in the supplementary table. The ACMG criteria were assessed by the Intervar. The variants were also confirmed from Human Gene Mutation Database (HGMD) and PMID number of the articles were retrieved and mentioned in supplementary table. The GnomAD frequency was also taken for the variants.

## Results

A total of 1647 results were retrieved after literature searching. After reviewing their titles and abstracts, full articles were retrieved for 326 citations. After final review, 87 studies were excluded resulting in 239 studies to be included in analysis. The reasons for exclusion were reviews, no variants reported and no novel variants (Supplementary Table 1).

**Fig. 2:**
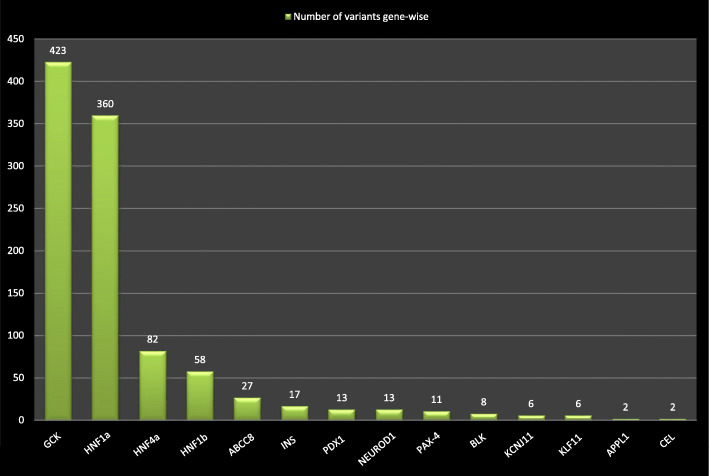
Number of gene wise variants identified in review. The x-axis showing the name of the gene while y-axis presenting the number of variants in literature. GCK gene being the highest in number of variants identified

**Fig. 3:**
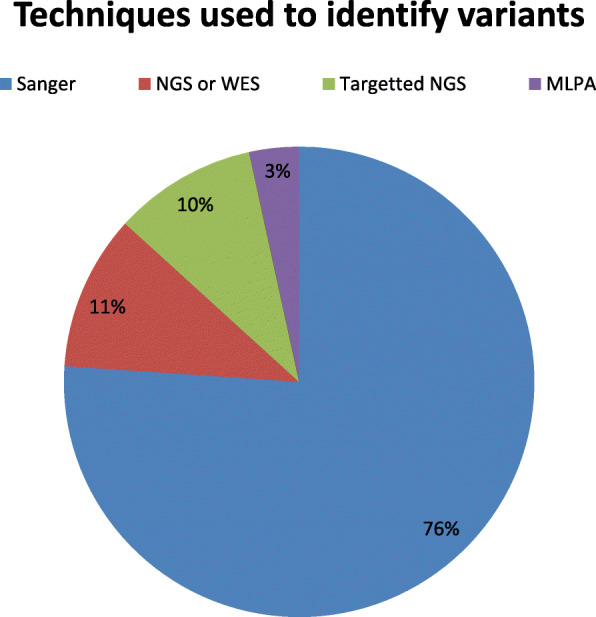
Percentage of different methods used for reported variants in MODY. Sanger sequencing was most commonly used as compared to others

The most frequently reported mutated gene was *GCK*, followed by *HNF1a* (Fig. 2). When analyzing the methods employed for identifying the variants causing MODY, a majority (76 %) used traditional Sanger sequencing while 11 % used whole exome sequencing (Fig. 3). The whole exome sequencing and targeted next generation sequencing were reported from studies from 2012 onwards till 2019. These techniques have played major role in identifying the new MODY types. The variants in *PDX1, INS, ABCC8, KCNJ11, NEUROD1, KLF11, BLK* and *APPL1* were identified through targeted next generation sequencing or whole exome sequencing. Significantly more (65 %) studies were conducted in 2011-2019 as compared to 23.5 % in 2001-2010 and 11.5 % in1990-2000.

**Fig. 4:**
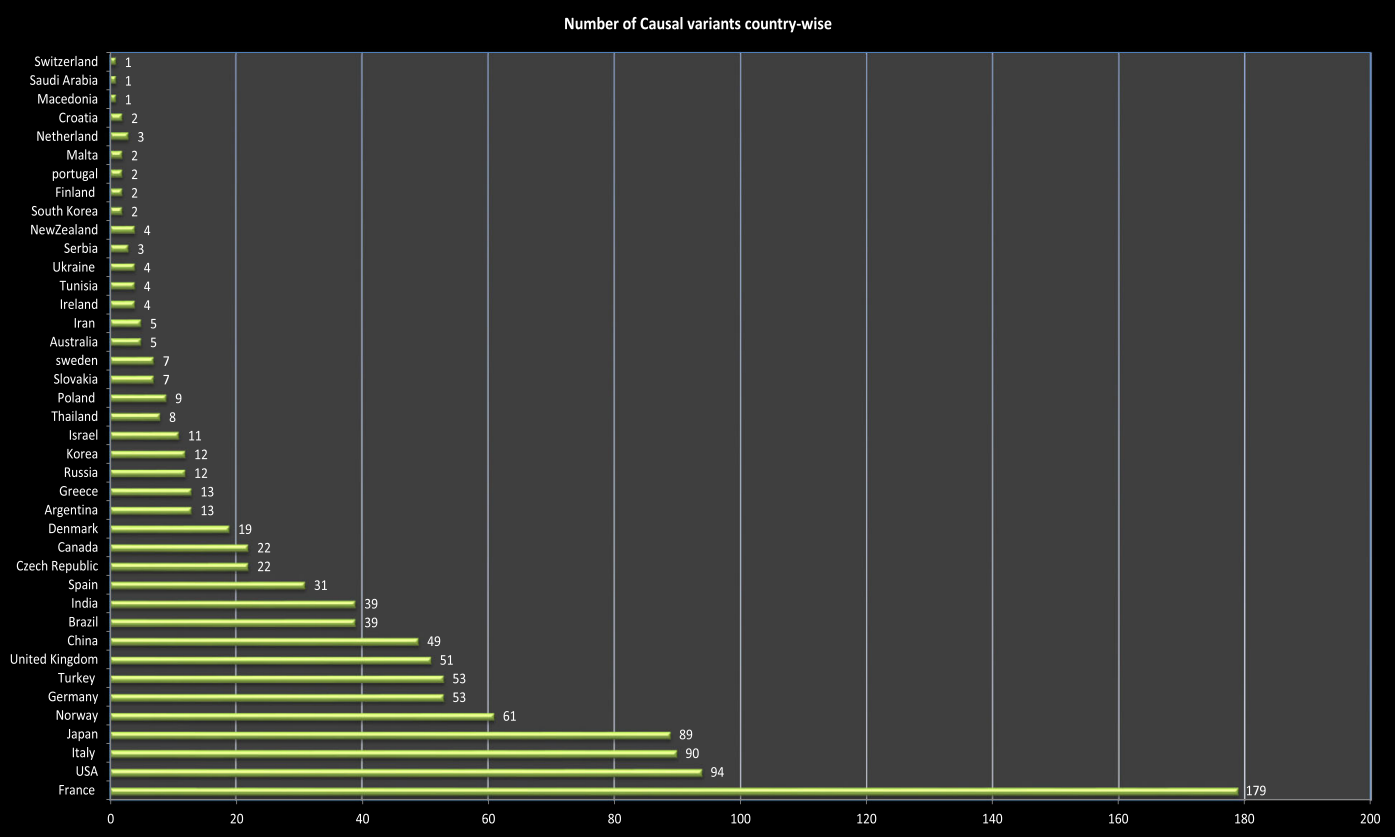
Graph showing Country-wise number of causal variants reported in the literature. The number of variants is presented on the x-axis while countries are shown on Y-axis. More variants were reported from France than other countries

When analyzing according to the countries, variants in MODY genes were reported from every region of the world. However there are few studies from countries like Jordan, Oman and Tunisia reporting that a majority of the MODY types that are prevalent worldwide were not common in their countries [6–8]. The largest number of variants was found from the French population followed by Italy, Japan and United States of America (Fig. 4).

A total of 1017 variants were identified through literature review in 14 known genes and attached as supplementary Table 2. A total of 94 pathogenic variants were identified from ClinVar and 20 among them were already in the list and were therefore excluded. The list of those 74 variants is attached as supplementary Table 3.

## Discussion

This is the comprehensive systematic review to investigate the causal variants in the 14 genes of MODY listed in OMIM, as reported so far in the literature. We found 1017 variants with majority in *GCK* and *HNF1a* from the published literature and 74 from Clinvar. This review provides the comprehensive list of causal variants identified so far for MODY.

The first causal variant in the *GCK* gene was identified in 1992 [9]. The glucokinase (*GCK*)gene is member of hexokinase proteins. It plays its role in first step in metabolic pathway i.e. conversion of glucose to glucose 6 phosphate. This type of MODY is characterized by mild fasting hyperglycemia and Hemoglobin A1C ranges from 5.6 to 7.3 % [10]. It is not known how many cases remain undiagnosed, and prevalence depends on screening and referral patterns in these asymptomatic patients. True prevalence may be even higher.

The MODY3 (*HNF1a*) variants constituted the second most common among all types. The gene encodes a widely expressed transcription factor, whose haplo-insufficiency appears to be deleterious specifically for beta-cells [11]. The patients with MODY 3 had variable symptoms with appearance at early adult life and increasing hyperglycemia with increased risk of micro-vascular and macro-vascular complications. The patients with HNF1A MODY are sensitive to sulphonylurea and low dose of sulphonylurea is generally the first line of treatment for these patients [12].

The variants in *HNF4a* (MODY1) were the 3rd most common in the list. This gene encodes a transcription factor. Its symptoms is similar to the MODY3 [13]. Variants in *INS*, encoding a protein regulating crucial metabolic processes [14] lead to misfolding of insulin and defective trafficking. The clinical severity varies among different cases [15, 16]. MODY12 (*ABCC8*) variants were also found in the literature and the symptoms were similar to the MODY1 and 3 i.e. *HNF4a* and *HNF1a*. The *ABCC8* gene contains 39 exons and encodes the sulphonylurea receptor 1 protein that controls insulin release [17]. Another type of MODY is MODY4 (*PDX1*), The *PDX1* gene is involved in insulin gene transcription regulation [18]. Its symptoms may include overweight or obesity in some cases [19]. Dorsal pancreatic agenesis was reported with this MODY type [20].

In *NEUROD1* (MODY6) we found 13 variants. This gene belong to basic helix loop helix transcription factors and played a role in transcription of E-box genes [21]. The PAX4 gene is mutated in MODY9 and is essential for beta cell generation during pancreas development [22]. The other five types i.e. *BLK* (MODY 11), *KCNJ11* (MODY 13), *KLF11* (MODY7), *APPL1* (MODY 14) and *CEL* (MODY 8) have less than 10 variants each that were found in our literature review.

The use of latest technologies like targeted next generation sequencing and whole exome sequencing become crucial for identification of new gene variant involved in the MODY. It is reported in the literature that for many rare diseases, new genes were identified by the use of these latest techniques [23–25]. It is recommended that the latest technologies (WES) must be used so that identification of new variants in the genes involved in MODY can be identified.

It was observed that countries like Tunisia [8, 26] Oman [6] Jordan [7] reported that MODY types that are more common in Caucasian population were not common in their countries. This implies to the fact that there might be other genes to be involved in early onset of diabetes in these countries. Therefore there is a need for whole exome studies of suspected MODY patients from these countries so that new genes or types involved in the MODY can be identified. There may be the recessive mutations involved, as reported in China for the early onset of diabetes due to non-syndromic recessive WFS1 mutations [27]. As these countries like Tunisia, Oman, Jordan, Pakistan have high rate of consanguinity [28–32] so there may be possible forms of recessive mutations causing diabetes.

In the last decade, there are other studies that reported genes other than known OMIM MODY genes to be involved in early onset of diabetes and MODY i.e. *RFX6* and *WFS1* genes [33, 34].

The limitation of this review was that we only collect the novel and pathogenic variants from published studies, a source that may distort the relative prevalence of different genes, based on how distinct the phenotype is (e.g. MODY2) or which genes are included in the diabetes panels. In the future, the systematic use of exome sequencing will reveal the true relative prevalence.

## Conclusions

The results will help clinicians in interpret MODY genetics results with greater confidence. Discrepancies in certain middle-eastern countries need to be investigated as other genes or factors, like consanguinity may be involved in developing diabetes.

### Author information

IR is PhD student and worked as Graduate Research Trainee at McGill University Health Research Institute.

## Supplementary information

Additional file 1 **Appendix A: Supplementary Table 1**.

Additional file 2 **Appendix B: Supplementary Table 2**.

Additional file 3 **Appendix C: Supplementary Table 3**.

## Data Availability

The datasets used and/or analysed during the current study are available from the corresponding author on reasonable request.

## Abbreviations

MODY: Maturity Onset Diabetes of the Young
HGM: Human Genome Mutation Data base
PMID: Pubmed Identification Number
OMIM: Online Mendelian Inheritance in Man
