# Supplementary material for: Causal variants in Maturity Onset Diabetes of the Young (MODY) – A systematic review
Source: BMC Endocr Disord. 2021 Nov 11;21:223. doi: 10.1186/s12902-021-00891-7 (PMC8582101; doi:10.1186/s12902-021-00891-7)
Supplement: Supplementary file 1 — Appendix A: Supplementary Table 1. [file 12902_2021_891_MOESM1_ESM.docx]

### Supplementary table 1: Full-text studies excluded from the final analysis

| **S. No** | **Reasons for exclusion** | **Number of studies** |
| --- | --- | --- |
|  | No novel variants | 39 |
|  | No variants described in 14 known MODY genes | 46 |
|  | Reviews | 2 |
|  | **Total** | **87** |

**Reason for exclusion:**

| **S.No** | **Excluded studies** | **Reason** |
| --- | --- | --- |
|  | Early Onset of Liver Steatosis in a Japanese Girl with Maturity-Onset Diabetes of the Young Type 3 (MODY3)  [Nakamura A](https://www.ncbi.nlm.nih.gov/pubmed/?term=Nakamura%20A%5BAuthor%5D&cauthor=true&cauthor_uid=22672869) 2012  PMID: 22672869 | No novel variant |
|  | Pregnancy Complicated by Maternal MODY 3 and Paternal MODY 2 Diabetes and Subsequent Rapidly Falling Insulin Requirement  Mikuscheva 2018  PMID 30356406 | No novel variant |
|  | Maturity-onset diabetes of the young type 5:a case report  Bañares JJ 2011  PMID: 22233861 | No variant described |
|  | Using highly sensitive C-reactive protein measurement to diagnose MODY in a family with suspected type 2 diabetes  Besser 2012, 22787179 | No variant described |
|  | HNF1A gene p.I27L is associated with earlyonset, maturity-onset diabetes of the  young-like diabetes in Turkey  Beysel S 2019,  PMID: 31109344 | Association studies |
|  | GCK-MODY in the US National Monogenic Diabetes Registry: Frequently Misdiagnosed and Unnecessarily Treated  Carmody 2016  PMID 27106716 | No variant described |
|  | Next-generation sequencing identifies monogenic diabetes in 16% of patients with late adolescence/adult-onset diabetes  selected on a clinical basis: a crosssectional  analysis  Donath, 2019  PMID: 31291970 | No variant described |
|  | Hepatocyte nuclear factor 1-alpha mutation  in normal glucose-tolerant subjects and early onset type 2 diabetic patients  Lim 2008, PMID: 19119252 | No novel variant |
|  | Diabetic ketoacidosis in the setting of  HNF1A-maturity onset diabetes of the young  Egan, PMID: 25837654 | NO novel variant |
|  | First UK survey of paediatric type 2 diabetes and MODY  Ehtisham 2004, 15155395 | No variant described |
|  | Optimal Glycemic Control in a Patient With HNF1A MODY With GLP-1 RA  Monotherapy: Implications for Future Therapy  Fantasia, 2018, PMID: 31737858 | No variant described |
|  | Prevalence of monogenic diabetes amongst Polish children after a nationwide genetic screening campaign  W. Fendler 2012, PMID: 22782286 | No variant described |
|  | An evolving spectrum of diabetes in a woman with GCK-MODY  Garrahy 2019, PMID: 30608898 | No novel variant |
|  | Maturity-Onset Diabetes of the Young Caused by a Balanced Translocation Where the 20q12 Break Point Results in Disruption Upstream of the Coding Region of Hepatocyte Nuclear Factor-4_ (HNF4A) Gene  Anna L. Gloyn 2002, PMID: 12086970 | No variant described |
|  | Assessment of Newly Proposed Clinical Criteria to Identify HNF1A MODY in Patients with an Initial Diagnosis of Type 1 or Type 2 Diabetes Mellitus  Grzanka 2016, PMID: 26942212 | No variant described |
|  | Exploring Phenotype-Genotype Correlations Using Interstitial Glucose Results in a Family With a Glucokinase Mutation  Lunt 2018, PMID: 29944009 | No variant described |
|  | Low Frequencies of Autoimmunity-Associated  PTPN22 Polymorphisms in MODY Patients,  Including Those Transiently Expressing Islet Cell  Autoantibodies  Heneberg 2015, PMID: 25896041 | No variant described |
|  | Assessing the phenotypic effects in the general population of rare variants in genes for a dominant mendelian form of diabetes  Flannick2013, PMID: 24097065 | No novel variant |
|  | Identification of a Locus for Maturity-Onset Diabetes of the Young on Chromosome 8p23  Kim 2004, PMID: 15111509 | No variant described |
|  | Structural and Functional Study of the GlnB22-Insulin Mutant Responsible for Maturity-Onset Diabetes of the Young  Krˇı´zˇkova´ 1 2014, PMID: 25423173 | No novel variant |
|  | Juvenile-Onset Diabetes and Congenital Cataract:  “Double-Gene” Mutations Mimicking Syndromic Diabetes Presentation  Lenfant 2017, PMID: 29112131 | Not relevant |
|  | Dysmorphic Features, Frontal Cerebral Cavernoma, and Hyperglycemia in a Girl with a De Novo Deletion of 7.23 Mb in Region 7p13 p12.1  López 2017, PMID: 28387648 | Syndromic |
|  | Non-penetrance in a MODY 3 family with a  mutation in the hepatic nuclear factor 1á gene:  implications for predictive testing  Miedzybrodzka, 1999, PMID: 10482964 | No novel or causal variant |
|  | Clinical and molecular characterization of  maturity onset-diabetes of the young caused by hepatocyte nuclear factor-4 alpha mutation: red flags for prediction of the diagnosis  Mohamed 2014, PMID: 25266181 | No novel variant |
|  | Functional Investigations of HNF1A Identify Rare Variants as Risk Factors for Type 2 Diabetes in the General Population  Najmi 2017, PMID: 27899486 | No variant described |
|  | Low serum level of high-sensitivity C-reactive  protein in a Japanese patient with maturityonset diabetes of the young type 3 (MODY3)  Ohki 2014, PMID: 25411618 | No novel variant |
|  | Maturity Onset Diabetes of the Young due  to Glucokinase, HNF1-A, HNF1-B, and HNF4-A  Mutations in a Cohort of Turkish Children Diagnosed as Type 1 Diabetes Mellitus  Ozsu, 2018, PMID: 30481753 | No novel variant |
|  | Heterozygous RFX6 protein truncating variants are associated with MODY with reduced penetrance  Patel 2017, PMID: 29026101 | No variants described |
|  | Maturity Onset Diabetes of the Young is Not  Necessarily Associated with Autosoma Inheritance: Case Description of a De Novo HFN1A Mutation  Salzano 2019, PMID: 31098941 | No novel variant |
|  | Genetic causes of maturity onset diabetes of the young may be less prevalent in American pregnant women recently diagnosed with diabetes mellitus than in previously studied European populations  . Sewell 2015, PMID: 25012807 | No variants described |
|  | Digenic heterozygous HNF1A and HNF4A mutations in two siblings with childhood-onset diabetes  Shankar 2013, PMID: 23551881 | No novel variant |
|  | A UK nationwide prospective study of treatment change in MODY: genetic subtype and clinical characteristics predict optimalglycaemic control after discontinuing insulin and metformin  Shepherd 2018, PMID: 30229274 | No variant described |
|  | Can Biomarkers Help Target Maturity-Onset  Diabetes of the Young Genetic Testing  in Antibody-Negative Diabetes?  Majidi 2018, PMID: 29355436 | No variant described |
|  | Expression of mutant mRNA and protein in  pancreatic cells derived from MODY3- iPS cells  Yabe 2019, PMID: 31145732 | No variant described |
|  | Novel Presentations of Congenital Hyperinsulinism due to Mutations in the MODY genes: HNF1A and HNF4A  Stanescu 2012, PMID: 22802087 | No novel variant |
|  | In silico and in vitro analyses of the  pathological relevance of the R258H mutation  of hepatocyte nuclear factor 4a identified in  maturity-onset diabetes of the young type 1  Sugawara 2019, PMID: 30325586 | No novel variant |
|  | Genetic testing for monogenic diabetes using  targeted next-generation sequencing in  patients with maturity-onset diabetes of the young  Szopa 2015, PMID: 26552609 | No variant described |
|  | Systematic Assessment of Etiology in  Adults With a Clinical Diagnosis of Young-Onset Type 2 Diabetes Is a Successful Strategy for Identifying Maturity-Onset Diabetes of the Young  THANABALASINGHAM, 2012, PMID: 22432108 | No novel variant |
|  | Letter to the Editor  Identification of novel and recurrent glucokinase mutations in Belgian and Luxembourg maturity onset diabetes of the young patients  Vits 2006, PMID: 16965331 | No novel variant |
|  | Bilateral Cataracts in a 6-year-old with New Onset Diabetes: A novel presentation of a known INS gene mutation  Wasserman, 2016, PMID: [26530398](https://www.ncbi.nlm.nih.gov/pubmed/26530398) | No novel variant |
|  | Clinically-Defined Maturity Onset Diabetes of  the Young in Omanis Absence of the common Caucasian gene mutations  Woodhouse 2010, PMID: 21509085 | No variants |
|  | Assessing the Pathogenicity, Penetrance,  and Expressivity of Putative Disease-Causing Variants in a Population Setting  Wright 2019, PMID: 30665703 | No variants for MODY |
|  | Studies of genetic variability of the  hepatocyte nuclear factor‑1α gene in an Indian  maturity‑onset diabetes of the young family  Yang, 2016, PMID: 27148439 | No pathogenic variants |
|  | Like-Triple Diabetes as First  Manifestation of MODY2 in an Overweight Teenager With Transient Multiple Antibodies  Wedrychowicz 2014, PMID: 24652732 | No variant described |
|  | Absence of Islet Autoantibodies  and Modestly Raised Glucose Values at Diabetes Diagnosis Should Lead to Testing for MODY: Lessons From a 5-Year Pediatric Swedis National Cohort Study  Carlsson 2019, PMID: 31704690 | No variant described |
|  | Linkage of maturity-onset diabetes of the young to the glucokinase gene - evidence of genetic heterogeneity  Hattersley 1992, PMID: 8449303 | No variant described |
|  | Structure–function studies of HNF1A  (MODY3) gene mutations in South Indian patients with monogenic diabetes  Balamurugan 2016, PMID: 26853433 | No novel variant |
|  | Bioinformatic detection of copy number variation in HNF4A causing maturity onset diabetes of the young  Berberich 2019, PMID: 31309534 | No variant described. |
|  | The coexistence of type 1 diabetes, MODY2 and metabolic syndrome in a young girl  Calcaterra 2012, PMID: 21688019 | No novel variant |
|  | Exonic Duplication of the Hepatocyte Nuclear Factor-1_ Gene (Transcription Factor 2, Hepatic) as a Cause of Maturity Onset Diabetes of the Young Type 5  Carette 2007, PMID: 17440011 | No variant described |
|  | Research: Pregnancy  An Irish National Diabetes in Pregnancy Audit aiming for best outcomes for women withdiabetes  Egan 2019, PMID: 30710451 | No variant described |
|  | From Biology to Genes and Back Again: Gene Discovery for Monogenic Forms of Beta-Cell  Dysfunction in Diabetes  De Franco 2016, PMID: 31479665 | Review |
|  | Identification of Twelve Novel Mutations in Patients With Classic and Variant Forms of Maple Syrup Urine Disease  Henneke 2003, PMID: 14517957 | No variant for MODY |
|  | Molecular background and clinical characteristics of HNF1A MODY in a Polish population  Skupien 2008, PMID: 18838325 | No novel variant |
|  | Whole-exome sequencing for mutation detection in pediatric disorders of insulin secretion: Maturity onset diabetes of the young and congenital hyperinsulinism  Johnson 2017, PMID: 29417725 | No novel variant |
|  | HNF1α Mutations Are Present in Half of Clinically Defined MODY Patients in South-Brazilian Individuals  MARASCHIN 2008, PMID: 19169489 | No novel variant |
|  | A case of Type-1 diabetes mellitus formerly diagnosed as maturity-onset diabetes of the young (MODY) carrying suggestive MODY3 gene  Miura 1997, PMID: 9483378 | No variant described |
|  | An analysis of the sequence of the BAD gene among patients with maturity-onset diabetes  Antosik 2017, PMID: 27935851 | Not relevant gene |
|  | A Common Haplotype for the 677T Thermolabile  Variant of the 5,10-Methylenetetrahydrofolate Reductase Gene in Thrombophilic Patients and  Controls  Linnebank 2002, PMID: 12442281 | No relevant variant |
|  | High Prevalence of Rare Monogenic Forms of Obesity in Obese Guadeloupean Afro-Caribbean Children  Foucan 2018, PMID: 29216354 | No relevant variant described |
|  | Monogenic diabetes prevalence among Polish children— Summary of 11 years-long nationwide geneti screening program  Małachowska 2018, PMID: 28436179 | No variant described |
|  | Onset of type 1 diabetes mellitus in two  patients with maturity onset diabetes of the young  Maltoni G, 2012, PMID: 21696527 | Not relevant |
|  | GCK-MODY diabetes as a protein misfolding disease: The mutation R275C promotes protein misfolding, self-association and cellular degradation  Negahdar M 2014, PMID: 24001579 | No novel variant |
|  | Meglitinide Analogues in Adolescent Patients With HNF1A-MODY (MODY 3)  Marianne Becker 2013, PMID: 24567025 | No variant described |
|  | High prevalence of glucokinase mutations in italian children with MODY. Influence on glucose tolerance, first phase insulin response, insulin sensitivity and BMI  Massa 2001, PMID: 11508276 | No novel variant |
|  | Case Report  An infant with combination gene mutations for  Monogenic Diabetes of Youth (MODY) 2 and  4, presenting with Diabetes Mellitus Requiring  Insulin (DMRI) at 8 months of age  Odem 2009, PMID: 19515026 | No novel variant |
|  | A description of clinician reported diagnosis of type 2 diabetes and other non-type 1 diabete included in a large international multicentered pediatric diabetes registry (SWEET)  Pacaud 2016, PMID: 27748026 | No variant described |
|  | Short Report: Epidemiology  Substantial proportion of MODY among multiplex families participating in a Type diabetes prediction  Petruzelkova 2015, PMID: 26641800 | No novel variant |
|  | Prevalence of  Maturity-Onset Diabetes of the Young Mutations in Brazilian Families With Autosomal- Dominant Early- Onset Type 2 Diabetes  Moises 2001, PMID: 11315851 | No novel variant |
|  | Dimerization defective MODY mutations of hepatocyte nuclear factor 4α  Singh, 2019, PMID: 30648609 | No variant described |
|  | Screening of mutations in the GCK gene in Jordanian maturity-onset diabetes of the young type 2 (MODY2) patients  Khalil, 2009, PMID: 19551638 | No variant described |
|  | Etiology of Early-Onset Type 2 Diabetes in Indians: Islet Autoimmunity and Mutations in Hepatocyte Nuclear Factor 1_ and Mitochondrial Gene  Ravi 2007, PMID: 17440016 | No novel variant |
|  | Phenotype, genotype and glycaemic variability in people with activating mutations in the ABCC8 gene response to appropriate therapy  Reilly 2019, PMID: 31562829 | No variant described |
|  | Diagnostic screening of  NEUROD1 (MODY6) in subjects  with MODY or gestational diabetes mellitus  Sagen 2005, PMID: 16026366 | No novel variant |
|  | A Prevalent Amino Acid Polymorphism at Codon 98 (Ala98Val) of the Hepatocyte Nuclear Factor-1 Is Associated With Maturity-Onset Diabetes of the Young and Younger Age at Onset of Type 2 Diabetes Younger Age at Onset of Type Diabetes in Asian Indians  ANURADHA 2005, PMID: 16186275 | No variant described |
|  | Minigene splicing assessment of 20 novel synonymous and intronic glucokinase gene variants identified in patients with maturity‐onset diabetes of the young  Tiulpakov 2019, PMID: 31529753 | No variant described |
|  | The unique clinical spectrum of maturity onset diabetes of the young type 3  Lebenthal 2017, PMID: 29107759 | No novel variant |
|  | Hepatocyte Nuclear Factor-1_ Gene Inactivation:  Cosegregation between Liver Adenomatosis and  Diabetes Phenotypes in Two Maturity-Onset Diabetes of the Young (MODY)3 Families  REZNIK 2004, PMID: 15001650 | No novel variant |
|  | Hepatocyte nuclear factor-α genetic mutation in a  Chinese pedigree with maturity-onset diabetes of  the young (MODY3)  Zhang 2015, PMID: 26436572 | No novel variant |
|  | Mutation screening of the hepatocyte nuclear factor (HNF)-6 gene in Japanese subjects with diabetes mellitus  Zhu 2001, PMID: 11323086 | No variant described |
|  | Heterozygous lys169Glu mutation of glucokinase gene in a Chinese family having glucokinas maturity-onset diabetes of the young (GCK-MODY)  [Zhou](https://www.ncbi.nlm.nih.gov/pubmed/?term=Zhou%20W%5BAuthor%5D&cauthor=true&cauthor_uid=31571622) 2019, PMID: 31571622 | No novel variant |
|  | Variation in Maturity-Onset Diabetes of the Young Genes Influence Response to Interventions for Diabetes Prevention  [Billings](https://www.ncbi.nlm.nih.gov/pubmed/?term=Billings%20LK%5BAuthor%5D&cauthor=true&cauthor_uid=28453780) 2017, PMID: 28453780 | No variants described |
|  | Molecular diagnosis of maturity onset diabetes of the young in India  [Nair](https://www.ncbi.nlm.nih.gov/pubmed/?term=Nair%20VV%5BAuthor%5D&cauthor=true&cauthor_uid=23869298) 2013, PMID: 23869298 | Review |
|  | Familial early-onset diabetes is not a typical MODY in several Tunisian patients.  [Amara](https://www.researchgate.net/profile/Abdelbasset_Amara) 2012, PMID: 23247789 | No novel variant |
|  | Davis TM, Makepeace AE, Ellard S, Colclough K, Peters K, The prevalence of monogenic diabetes in Australia: the Fremantle Diabetes Study Phase II.  Davis 2017, PMID: 29020906 | No novel variant |
|  | Glucokinase diabetes in 103 families from a country-based study in the Czech Republic: geographically restricted distribution of two prevalent GCK mutations.  Pruhova2010,PMID: 20337973 | No novel variant |
|  | Prevalence of diabetes in Australia: insights from the Fremantle Diabetes Study Phase II.  Davis 2018, PMID: 29512259 | No novel variant |
