# Supplementary material for: Causal variants in Maturity Onset Diabetes of the Young (MODY) – A systematic review
Source: BMC Endocr Disord. 2021 Nov 11;21:223. doi: 10.1186/s12902-021-00891-7 (PMC8582101; doi:10.1186/s12902-021-00891-7)
Supplement: Supplementary file 2 — Appendix B: Supplementary Table 2. [file 12902_2021_891_MOESM2_ESM.docx]

**Supplementary table 2: List of variants reported in known 14 MODY genes identified through literature review**

| **S.No** | ***Gene*** | **Nucleotide position** | **Protein position** | **Accession number**  **(PMID number from HGMD)** | **Gnom AD frequency** | **Country** | **ACMG**  **(Intervar)*** | **Publication Year** | **Reference** |
| --- | --- | --- | --- | --- | --- | --- | --- | --- | --- |
|  | *GCK* | c.908G>T | p.Arg303Leu | NM_001354800.1 NP_001341729.1  (25921421) | NA | Greece | LP | 2015 | ([1](#_ENREF_1)) |
|  | *GCK* | c.748C>T | p.Arg250Cys | NP_000153.1  (17204055) | NA | Serbia | VUS | 2006 | ([2](#_ENREF_2)) |
|  | *GCK* | c.182G>A | p.T61I | NP_000153.1  (8433729) | NA | Spain | VUS | 2000 | ([3](#_ENREF_3)) |
|  | *GCK* | c.358C>T | p.A120T | ? | NA | Spain | VUS |  |  |
|  | *GCK* | c.238delT | M238fsdelT | NP_000153.1 | NA | Spain | - |  |  |
|  | *GCK* | c.226deltinsAA | V226fsdelTinsAA | NP_000153.1 | NA | Spain | - |  |  |
|  | *GCK* | c.intron418-7del11 | S418–7del11 | NP_000153.1 | NA | Spain | - |  |  |
|  | *GCK* | c.76C>T, | p.Gln26Ter | NP_000153.1  (11508276) | NA | Brazil | VUS | 2013 | ([4](#_ENREF_4)) |
|  | *GCK* | c.1012G > A | p.Q338X | NP_000153.1  (26226118) | NA | Turkey | P | 2015 | ([5](#_ENREF_5)) |
|  | *GCK* | c.755G > C | p.C252S | NP_000153.1  (26226118) | NA | Turkey | VUS |  |  |
|  | *GCK* | c.257T>C | p.Val86Ala | NP_000153.1  (26226118) | NA | Turkey | VUS |  |  |
|  | *GCK* | c.43A>T | p.Lys15* | NM_001354800.1  NP_001341729.1  (23771925) | NA | Italy | P | 2017 | ([6](#_ENREF_6)) |
|  | *GCK* | c.579G>T, | p.Gly193Gly | NM_001354800.1  NP_001341729.1 | NA | Italy | VUS |  |  |
|  | *GCK* | c.505A>G, | p.Lys169Glu | NP_000153.1 | NA | Italy | VUS |  |  |
|  | *GCK* | c.580-3C>A | - | NM_001354800.1  NP_001341729.1 | NA | Italy | - |  |  |
|  | *GCK* | c. 595G>A | p.Val199Met | NP_000153.1 | NA | Italy | VUS |  |  |
|  | *GCK* | c.944T>C, | p.Leu315Pro | NP_000153.1 | NA | Italy | LP |  |  |
|  | *GCK* | c.1229G>T, | p.Gly410Val | NP_000153.1 | NA | Italy | LP |  |  |
|  | *GCK* | c.1103_1122_del19nt_ | p.Arg368fs27* | NP_000153.1 | NA | Italy | - |  |  |
|  | *GCK* | c.1322 C>T | p.Ser441Leu | NP_000153.1 | NA | Italy | LP |  |  |
|  | *GCK* | c.1226delA | p.D409VfsX21 | NM_001354800.1  NP_001341729.1 | NA | Canada |  | 2016 | ([7](#_ENREF_7)) |
|  | *GCK* | c.880_891delinsCATGGCGAGCTGGTGT | p.Gly294HisfsTer179) | NM_001354800.1  NP_001341729.1 | NA | Argentina |  | 2016 | ([8](#_ENREF_8)) |
|  | *GCK* | c.895G>C | [p.Gly299Arg](https://variantvalidator.org/service/validate/) | NM_001354800.1  NP_001341729.1  (1303265) | NA | Argentina | LP |  |  |
|  | *GCK* | c.260T>C | p.Val86Ala | NP_000153.1  (26226118) | NA | Turkey | VUS | 2018 | ([9](#_ENREF_9)) |
|  | *GCK* | c.313delC | p.His105ThrfsX11 | NM_001354800.1  NP_001341729.1 | NA | Turkey |  |  |  |
|  | *GCK* | c.349G>A | p.Gly117Ser | NP_000153.1 | NA | Turkey | VUS |  |  |
|  | *GCK* | c.387C>A | p.Cys129X | NP_000153.1  (11508276) | NA | Turkey | P |  |  |
|  | *GCK* | c.390delC | p.Ser131ProfsX9 | NM_001354800.1 NP_001341729.1 | NA | Turkey |  |  |  |
|  | *GCK* | c.475A>G | p.Ile159Val | NM_001354800.1 NP_001341729.1  (21978167) | NA | Turkey | VUS |  |  |
|  | *GCK* | c.478G>C | p.Asp160His | NM_001354800.1  [NP_001341729.1](https://www.ncbi.nlm.nih.gov/nuccore/NP_001341729.1) | NA | Turkey | LP |  |  |
|  | *GCK* | c.512T>C | p.Phe171Ser | [NM_001354800.1](https://www.ncbi.nlm.nih.gov/nuccore/NM_001354800.1)  [NP_001341729.1](https://www.ncbi.nlm.nih.gov/nuccore/NP_001341729.1) | NA | Turkey | VUS |  |  |
|  | *GCK* | c.686delG | p.Gly229AfsX65 | [NM_001354800.1](https://www.ncbi.nlm.nih.gov/nuccore/NM_001354800.1)  [NP_001341729.1](https://www.ncbi.nlm.nih.gov/nuccore/NP_001341729.1) | NA | Turkey |  |  |  |
|  | *GCK* | c.713T>C | p.Met238Thr | [NM_001354800.1](https://www.ncbi.nlm.nih.gov/nuccore/NM_001354800.1)  [NP_001341729.1](https://www.ncbi.nlm.nih.gov/nuccore/NP_001341729.1) | NA | Turkey | VUS |  |  |
|  | *GCK* | c.819T >G | p. Tyr273X | [NM_001354800.1](https://www.ncbi.nlm.nih.gov/nuccore/NM_001354800.1)  [NP_001341729.1](https://www.ncbi.nlm.nih.gov/nuccore/NP_001341729.1) | NA | Turkey | LP |  |  |
|  | *GCK* | c.841T>G | p.Ser281Ala | [NM_001354800.1](https://www.ncbi.nlm.nih.gov/nuccore/NM_001354800.1)  [NP_001341729.1](https://www.ncbi.nlm.nih.gov/nuccore/NP_001341729.1) | NA | Turkey | VUS |  |  |
|  | *GCK* | c.950A>C | p.His317Pro | [NM_001354800.1](https://www.ncbi.nlm.nih.gov/nuccore/NM_001354800.1) [NP_001341729.1](https://www.ncbi.nlm.nih.gov/nuccore/NP_001341729.1) | NA | Turkey | LP |  |  |
|  | *GCK* | c.1055T>C | p.Leu352Pro | [NM_001354800.1](https://www.ncbi.nlm.nih.gov/nuccore/NM_001354800.1) [NP_001341729.1](https://www.ncbi.nlm.nih.gov/nuccore/NP_001341729.1) | NA | Turkey | VUS |  |  |
|  | *GCK* | c.1179_1192delGCGCGAGAGCCGCA | p.Glu395Glyfs*59 | [NM_001354800.1](https://www.ncbi.nlm.nih.gov/nuccore/NM_001354800.1)  [NP_001341729.1](https://www.ncbi.nlm.nih.gov/nuccore/NP_001341729.1) | NA | Turkey |  |  |  |
|  | *GCK* | c.1222G>T | p.Val408Leu | [NM_001354800.1](https://www.ncbi.nlm.nih.gov/nuccore/NM_001354800.1)  [NP_001341729.1](https://www.ncbi.nlm.nih.gov/nuccore/NP_001341729.1) | NA | Turkey | VUS |  |  |
|  | *GCK* | c.1256T>G | p.Pfe419Cys | [NM_001354800.1](https://www.ncbi.nlm.nih.gov/nuccore/NM_001354800.1)  [NP_001341729.1](https://www.ncbi.nlm.nih.gov/nuccore/NP_001341729.1)  (19790256) | NA | Turkey | VUS |  |  |
|  | *GCK* | c.1391_1392delGC |  | ? | NA | Turkey |  |  |  |
|  | *GCK* | IVS8+1delG | g.51709_51709delG | ? | NA | Turkey |  |  |  |
|  | *GCK* | c.208+3A>T |  | [NM_001354800.1](https://www.ncbi.nlm.nih.gov/nuccore/NM_001354800.1)  [NP_001341729.1](https://www.ncbi.nlm.nih.gov/nuccore/NP_001341729.1) | NA | Turkey |  |  |  |
|  | *GCK* | c.729delG | p.Val244TrpfsTer50 | [NM_001354800.1](https://www.ncbi.nlm.nih.gov/nuccore/NM_001354800.1)  [NP_001341729.1](https://www.ncbi.nlm.nih.gov/nuccore/NP_001341729.1) | NA | Czech Republic |  | 2010 | ([10](#_ENREF_10)) |
|  | *GCK* | c.1268T>A | p.Phe423Tyr | [NM_001354800.1](https://www.ncbi.nlm.nih.gov/nuccore/NM_001354800.1)  [NP_001341729.1](https://www.ncbi.nlm.nih.gov/nuccore/NP_001341729.1)  (19790256) | NA | Portugal | LP | 2014 | ([11](#_ENREF_11)) |
|  | *GCK* | c.718A>G | p.Asn240Asp | [NM_001354800.1](https://www.ncbi.nlm.nih.gov/nuccore/NM_001354800.1)  [NP_001341729.1](https://www.ncbi.nlm.nih.gov/nuccore/NP_001341729.1)  (24735133) | NA | Italy | VUS | 2015 | ([12](#_ENREF_12)) |
|  | *GCK* | c.757G>T | p.Val253Phe | [NM_001354800.1](https://www.ncbi.nlm.nih.gov/nuccore/NM_001354800.1)  [NP_001341729.1](https://www.ncbi.nlm.nih.gov/nuccore/NP_001341729.1)  (24735133) | NA | Italy | LP |  |  |
|  | *GCK* | c.872A>C | p.Lys291Thr | [NM_001354800.1](https://www.ncbi.nlm.nih.gov/nuccore/NM_001354800.1)  [NP_001341729.1](https://www.ncbi.nlm.nih.gov/nuccore/NP_001341729.1)  (24735133) | NA | Italy | VUS |  |  |
|  | *GCK* | c.1151C>T | p.Ala384Val | [NM_001354800.1](https://www.ncbi.nlm.nih.gov/nuccore/NM_001354800.1)  [NP_001341729.1](https://www.ncbi.nlm.nih.gov/nuccore/NP_001341729.1)  (24735133) | NA | Italy | VUS |  |  |
|  | *GCK* | c.175C>T | p.Pro59Ser | [NM_001354800.1](https://www.ncbi.nlm.nih.gov/nuccore/NM_001354800.1)  [NP_001341729.1](https://www.ncbi.nlm.nih.gov/nuccore/NP_001341729.1)  (22060211) | NA | Italy | LP | 2013 | ([13](#_ENREF_13)) |
|  | *GCK* | c.49G>T | p.Glu17X | [NM_001354800.1](https://www.ncbi.nlm.nih.gov/nuccore/NM_001354800.1)  [NP_001341729.1](https://www.ncbi.nlm.nih.gov/nuccore/NP_001341729.1)  (22335469) | NA | Italy | P |  |  |
|  | *GCK* | c.1114G>T | p.Glu372X | [NM_001354800.1](https://www.ncbi.nlm.nih.gov/nuccore/NM_001354800.1) [NP_001341729.1](https://www.ncbi.nlm.nih.gov/nuccore/NP_001341729.1)  (22335469) | NA | Italy | P |  |  |
|  | *GCK* | c.118G>A | p.Glu40Lys | NP_000153.1  (12627330) | NA | Czech Republic | LP | 2012 | ([14](#_ENREF_14)) |
|  | *GCK* | c.944T>A | p.Leu315His | NP_000153.1  (17204055) | NA | Czech Republic | LP |  |  |
|  | *GCK* | c.952G>A | p.Gly318Arg | NP_000153.1  (12627330) | NA | Czech Republic | LP |  |  |
|  | *GCK* | c.98T>C | p.Val33Ala | NP_000153.1  (18271687) | NA | Czech Republic | LP |  |  |
|  | *GCK* | c.605T>C | p.Met202Thr | NP_000153.1  (17937063) | NA | Israel |  | 2007 | ([15](#_ENREF_15)) |
|  | *GCK* | c.616A>C | p.T206P | NP_000153.1  (17937063) | NA | Israel | LP |  |  |
|  | *GCK* | c.1113C>A | p.Cys371X | NP_000153.1 |  | Greece |  | 2020 | ([16](#_ENREF_16)) |
|  | *GCK* | c.605T>C | p.Met202Thr | [NM_001354800.1](https://www.ncbi.nlm.nih.gov/nuccore/NM_001354800.1)  [NP_001341729.1](https://www.ncbi.nlm.nih.gov/nuccore/NP_001341729.1)  (17937063) | NA | Spain | LP | 2007 | ([17](#_ENREF_17)) |
|  | *GCK* | c.1258A>G | p.Lys420Glu | [NM_001354800.1](https://www.ncbi.nlm.nih.gov/nuccore/NM_001354800.1)  [NP_001341729.1](https://www.ncbi.nlm.nih.gov/nuccore/NP_001341729.1)  (17573900) | NA | Spain | VUS |  |  |
|  | *GCK* | 96_98delAAG, | p.Lys32del | NP_000153.1 | NA | Brazil |  | 2013 | ([18](#_ENREF_18)) |
|  | *GCK* | c.533G>C | p.Gly178Ala | [NM_001354800.1](https://www.ncbi.nlm.nih.gov/nuccore/NM_001354800.1) [NP_001341729.1](https://www.ncbi.nlm.nih.gov/nuccore/NP_001341729.1) | NA | Brazil | LP | 2017 | ([18](#_ENREF_18), [19](#_ENREF_19)) |
|  | *GCK* | c.227C>G | p.Ser76Cys | [NM_001354800.1](https://www.ncbi.nlm.nih.gov/nuccore/NM_001354800.1)  [NP_001341729.1](https://www.ncbi.nlm.nih.gov/nuccore/NP_001341729.1)  (21978167) | NA | Israel | VUS | 2012 | ([20](#_ENREF_20)) |
|  | *GCK* | c.455T>C | p.Phe152Ser | [NM_001354800.1](https://www.ncbi.nlm.nih.gov/nuccore/NM_001354800.1)  [NP_001341729.1](https://www.ncbi.nlm.nih.gov/nuccore/NP_001341729.1)  (21978167) | NA | Israel | VUS |  |  |
|  | *GCK* | c.476T>A | p.Ile159Asn | [NM_001354800.1](https://www.ncbi.nlm.nih.gov/nuccore/NM_001354800.1)  [NP_001341729.1](https://www.ncbi.nlm.nih.gov/nuccore/NP_001341729.1)  (21978167) | NA | Israel | VUS |  |  |
|  | *GCK* | c.499T>C | p.Trp167Arg | [NM_001354800.1](https://www.ncbi.nlm.nih.gov/nuccore/NM_001354800.1)  [NP_001341729.1](https://www.ncbi.nlm.nih.gov/nuccore/NP_001341729.1)  (21978167) | NA | Israel | VUS |  |  |
|  | *GCK* | c.532G>T | p.Gly178Trp | [NM_001354800.1](https://www.ncbi.nlm.nih.gov/nuccore/NM_001354800.1)  [NP_001341729.1](https://www.ncbi.nlm.nih.gov/nuccore/NP_001341729.1)  (21978167) | NA | Israel | LP |  |  |
|  | *GCK* | c.820G>C | p.Asp274His | [NM_001354800.1](https://www.ncbi.nlm.nih.gov/nuccore/NM_001354800.1)  [NP_001341729.1](https://www.ncbi.nlm.nih.gov/nuccore/NP_001341729.1)  (21978167) | NA | Israel | VUS |  |  |
|  | *GCK* | c.567_568insTATC | p.Lys190fs197X | [NM_001354800.1](https://www.ncbi.nlm.nih.gov/nuccore/NM_001354800.1)  [NP_001341729.1](https://www.ncbi.nlm.nih.gov/nuccore/NP_001341729.1) | NA | Israel |  |  |  |
|  | *GCK* |  | p.Gly80Ser | NP_000153.1  (10694920) | NA | Italy |  | 1998 | ([21](#_ENREF_21)) |
|  | *GCK* | c.661G>A | p.Glu221Lys | NP_000153.1  (10694920) | NA | Italy |  |  |  |
|  | *GCK* |  | p.Gly227Cys | NP_000153.1  (10694920) | NA | Italy |  |  |  |
|  | *GCK* | c.579+1G>C |  | [NM_001354800.1](https://www.ncbi.nlm.nih.gov/nuccore/NM_001354800.1) [NP_001341729.1](https://www.ncbi.nlm.nih.gov/nuccore/NP_001341729.1) | NA | Norway |  | 2013 | ([22](#_ENREF_22)) |
|  | *GCK* | c.556C>G | p.Arg186Gly | [NM_001354800.1](https://www.ncbi.nlm.nih.gov/nuccore/NM_001354800.1)  [NP_001341729.1](https://www.ncbi.nlm.nih.gov/nuccore/NP_001341729.1)  (24097065) | NA | Norway | VUS |  |  |
|  | *GCK* | c.227C>A | p.Ser76Tyr | [NM_001354800.1](https://www.ncbi.nlm.nih.gov/nuccore/NM_001354800.1)  [NP_001341729.1](https://www.ncbi.nlm.nih.gov/nuccore/NP_001341729.1)  (21978167) | NA | Norway | VUS |  |  |
|  | *GCK* | c.1175G>C | p.Arg392Pro | [NM_001354800.1](https://www.ncbi.nlm.nih.gov/nuccore/NM_001354800.1)  [NP_001341729.1](https://www.ncbi.nlm.nih.gov/nuccore/NP_001341729.1)  (24097065) | NA | Norway | LP |  |  |
|  | *GCK* | c.629T>A | p.Met210Lys | [NM_001354800.1](https://www.ncbi.nlm.nih.gov/nuccore/NM_001354800.1)  [NP_001341729.1](https://www.ncbi.nlm.nih.gov/nuccore/NP_001341729.1)  (11372010) | NA | Norway | LP |  |  |
|  | *GCK* | c.697T>C | p.Cys233Arg | [NM_001354800.1](https://www.ncbi.nlm.nih.gov/nuccore/NM_001354800.1)  [NP_001341729.1](https://www.ncbi.nlm.nih.gov/nuccore/NP_001341729.1)  (17573900) | NA | Norway | VUS |  |  |
|  | *GCK* | c.579+1G>C |  | [NM_001354800.1](https://www.ncbi.nlm.nih.gov/nuccore/NM_001354800.1)  [NP_001341729.1](https://www.ncbi.nlm.nih.gov/nuccore/NP_001341729.1) | NA | Norway |  |  |  |
|  | *GCK* | c.766G>A | p.Glu256Lys | [NM_001354800.1](https://www.ncbi.nlm.nih.gov/nuccore/NM_001354800.1)  [NP_001341729.1](https://www.ncbi.nlm.nih.gov/nuccore/NP_001341729.1)  (8446612) | NA | Norway | LP |  |  |
|  | *GCK* | c.706G>A | p.Glu237Lys | NP_000153.1  (15305805) | NA | Canada |  | 2004  2003 | ([23](#_ENREF_23), [24](#_ENREF_24)) |
|  | *GCK* | c.971T>C | p.Leu324Pro | NP_000153.1  (15305805) | NA | Canada |  |  |  |
|  | *GCK* | IVS3 -8G>A |  | ? | NA | Canada |  |  |  |
|  | *GCK* | c.349G>C | p.G117R | [NM_001354800.1](https://www.ncbi.nlm.nih.gov/nuccore/NM_001354800.1)  [NP_001341729.1](https://www.ncbi.nlm.nih.gov/nuccore/NP_001341729.1) | NA | USA |  | 2015 | ([25](#_ENREF_25)) |
|  | *GCK* | c.397T>C | p.F133L | [NM_001354800.1](https://www.ncbi.nlm.nih.gov/nuccore/NM_001354800.1)  [NP_001341729.1](https://www.ncbi.nlm.nih.gov/nuccore/NP_001341729.1)  (25555642) | NA | USA | VUS |  |  |
|  | *GCK* | c.601G>C | p.A201P | [NM_001354800.1](https://www.ncbi.nlm.nih.gov/nuccore/NM_001354800.1)  [NP_001341729.1](https://www.ncbi.nlm.nih.gov/nuccore/NP_001341729.1)  (25555642) | NA | USA | LP |  |  |
|  | *GCK* | c.883G>T | p.G295C | [NM_001354800.1](https://www.ncbi.nlm.nih.gov/nuccore/NM_001354800.1)  [NP_001341729.1](https://www.ncbi.nlm.nih.gov/nuccore/NP_001341729.1)  (25555642) | NA | USA | LP |  |  |
|  | *GCK* | c.951delC | p.H317Qfs*36 | [NM_001354800.1](https://www.ncbi.nlm.nih.gov/nuccore/NM_001354800.1)  [NP_001341729.1](https://www.ncbi.nlm.nih.gov/nuccore/NP_001341729.1)  (25555642) | NA | USA |  |  |  |
|  | *GCK* | c.1113C>A | p.C371* | [NM_001354800.1](https://www.ncbi.nlm.nih.gov/nuccore/NM_001354800.1)  [NP_001341729.1](https://www.ncbi.nlm.nih.gov/nuccore/NP_001341729.1) | NA | USA |  |  |  |
|  | *GCK* | c.1268T>C | p.F423S | [NM_001354800.1](https://www.ncbi.nlm.nih.gov/nuccore/NM_001354800.1) [NP_001341729.1](https://www.ncbi.nlm.nih.gov/nuccore/NP_001341729.1)  (25555642) | NA | USA | VUS |  |  |
|  | *GCK* | c.464G>C | p.Arg155Thr | NP_000153.1  (19410318) | NA | Spain |  | 2009 | ([26](#_ENREF_26)) |
|  | *GCK* | c.764C>T | p.Thr255Ile | NP_000153.1  (19410318) | NA | Spain |  |  |  |
|  | *GCK* | c.995C>A | p.Thr332Lys | NP_000153.1  (19410318) | NA | Spain |  |  |  |
|  | *GCK* |  | p.His424Y | NP_000153.1  (19410318) |  | Spain |  |  |  |
|  | *GCK* |  | p.Arg449Ser | NP_000153.1  (19410318) |  | Spain |  |  |  |
|  | *GCK* |  | p.Tyr289X | NP_000153.1 |  | Spain |  |  |  |
|  | *GCK* |  | p.Lys143del | NP_000153.1 |  | Spain |  |  |  |
|  | *GCK* | c.571C>T | p.Arg191Trp | NP_000153.1  (10753050) |  | South Korea |  | 2006 | ([27](#_ENREF_27)) |
|  | *GCK* | c. 24G>A | p.Met8Ile | [NM_001354800.1](https://www.ncbi.nlm.nih.gov/nuccore/NM_001354800.1) [NP_001341729.1](https://www.ncbi.nlm.nih.gov/nuccore/NP_001341729.1)  (15928245) |  | Denmark | VUS | 2005 | ([28](#_ENREF_28)) |
|  | *GCK* | c.365T>C | p.Leu122Pro | [NM_001354800.1](https://www.ncbi.nlm.nih.gov/nuccore/NM_001354800.1)  [NP_001341729.1](https://www.ncbi.nlm.nih.gov/nuccore/NP_001341729.1)  (15928245) |  | Denmark | VUS |  |  |
|  | *GCK* | c.533G>A | p.Gly178Glu | [NM_001354800.1](https://www.ncbi.nlm.nih.gov/nuccore/NM_001354800.1)  [NP_001341729.1](https://www.ncbi.nlm.nih.gov/nuccore/NP_001341729.1)  (15928245) | NA | Denmark | LP |  |  |
|  | *GCK* | c.617C>T | p.Thr206Met | [NM_001354800.1](https://www.ncbi.nlm.nih.gov/nuccore/NM_001354800.1)  [NP_001341729.1](https://www.ncbi.nlm.nih.gov/nuccore/NP_001341729.1)  (11508276) | NA | Denmark | VUS |  |  |
|  | *GCK* | c.1019+1G>A | - | [NM_001354800.1](https://www.ncbi.nlm.nih.gov/nuccore/NM_001354800.1)  [NP_001341729.1](https://www.ncbi.nlm.nih.gov/nuccore/NP_001341729.1) | NA | Denmark |  |  |  |
|  | *GCK* | c.1183G>T | p.Glu395Ter | [NM_001354800.1](https://www.ncbi.nlm.nih.gov/nuccore/NM_001354800.1)  [NP_001341729.1](https://www.ncbi.nlm.nih.gov/nuccore/NP_001341729.1)  (15928245) | NA | Denmark | P |  |  |
|  | *GCK* | c.1210-1211delAT |  | NP_000153.1 |  | Denmark |  |  |  |
|  | *GCK* |  | p.Arg186X | NP_000153.1  (1360036) |  | France |  | 1994 | ([29](#_ENREF_29)) |
|  | *GCK* | c.626C>T | p.Ala209Val | NP_000153.1  (8168652) | 3.97687052104957E-06 | France |  |  |  |
|  | *GCK* |  | p.Gly261Glu | NP_000153.1  (8168652) |  | France |  |  |  |
|  | *GCK* |  | p.Arg36Trp | NP_000153.1  (8168652) |  | France |  |  |  |
|  | *GCK* | c.835_836del | p.Glu236Ala | NP_000153.1  [NM_001354800.1](https://www.ncbi.nlm.nih.gov/nuccore/NM_001354800.1)  (24804978) |  | Japan |  | 2004 | ([30](#_ENREF_30)) |
|  | *GCK* | c.864-2A>G |  | [NM_001354800.1](https://www.ncbi.nlm.nih.gov/nuccore/NM_001354800.1)  [NP_001341729.1](https://www.ncbi.nlm.nih.gov/nuccore/NP_001341729.1) |  | Japan |  |  |  |
|  | *GCK* | c.538A>C | p.Asn180His | [NM_001354800.1](https://www.ncbi.nlm.nih.gov/nuccore/NM_001354800.1)  [NP_001341729.1](https://www.ncbi.nlm.nih.gov/nuccore/NP_001341729.1)  (24804978) |  | Japan | VUS |  |  |
|  | *GCK* | c.635_637del | p.Ser212del | [NM_001354800.1](https://www.ncbi.nlm.nih.gov/nuccore/NM_001354800.1)  [NP_001341729.1](https://www.ncbi.nlm.nih.gov/nuccore/NP_001341729.1) |  | Japan |  |  |  |
|  | *GCK* | c.1055T>G | p.Leu352Arg | [NM_001354800.1](https://www.ncbi.nlm.nih.gov/nuccore/NM_001354800.1)  [NP_001341729.1](https://www.ncbi.nlm.nih.gov/nuccore/NP_001341729.1)  (24804978) | NA | Japan | VUS |  |  |
|  | *GCK* | c.707A>C | p.Glu236Ala | [NM_001354800.1](https://www.ncbi.nlm.nih.gov/nuccore/NM_001354800.1)  [NP_001341729.1](https://www.ncbi.nlm.nih.gov/nuccore/NP_001341729.1)  (24804978) | NA | Japan | VUS |  |  |
|  | *GCK* | c.1144- 1149dup | p.C382_S383dup | NP_000153.1 | NA | Japan |  |  |  |
|  | *GCK* | 32insC(33)intron3 |  | ? | NA | Germany |  | 2004 | ([31](#_ENREF_31)) |
|  | *GCK* | 39insC(40)intron3 |  | ? | NA | Germany |  |  |  |
|  | *GCK* | c.364C>A | p.Leu122Ile | NP_000153.1  (15216446) |  | Germany | LP |  |  |
|  | *GCK* | c.72G>R |  | NP_000153.1  (10447526) |  | Sweden |  | 1999 | ([32](#_ENREF_32)) |
|  | *GCK* | IVS3 + 1G > A |  | ? |  | Sweden |  |  |  |
|  | *GCK* | IVS3 ±2A > G |  | ? |  | Sweden |  |  |  |
|  | *GCK* | 133dupY125-D132 | p.Leu45ProfsTer7 | [NM_001354800.1](https://www.ncbi.nlm.nih.gov/nuccore/NM_001354800.1)  [NP_001341729.1](https://www.ncbi.nlm.nih.gov/nuccore/NP_001341729.1) |  | Sweden |  |  |  |
|  | *GCK* | c.1093G>A | p.Asp365Asn | [NM_001354800.1](https://www.ncbi.nlm.nih.gov/nuccore/NM_001354800.1)  [NP_001341729.1](https://www.ncbi.nlm.nih.gov/nuccore/NP_001341729.1)  (25174781) | NA | Brazil | VUS | 2014 | ([33](#_ENREF_33)) |
|  | *GCK* | c.242G>A | p.Gly81Asp | [NM_001354800.1](https://www.ncbi.nlm.nih.gov/nuccore/NM_001354800.1)  [NP_001341729.1](https://www.ncbi.nlm.nih.gov/nuccore/NP_001341729.1)  (20132997) | NA | Brazil | VUS |  |  |
|  | *GCK* | c.757G>C | p.Val253Leu | [NM_001354800.1](https://www.ncbi.nlm.nih.gov/nuccore/NM_001354800.1)  [NP_001341729.1](https://www.ncbi.nlm.nih.gov/nuccore/NP_001341729.1)  (25174781) | NA | Brazil | LP |  |  |
|  | *GCK* | c.571C>T | p.Arg191Trp | NP_000153.1  (10753050) | NA | Brazil | VUS | 2012 | ([34](#_ENREF_34)) |
|  | *GCK* | c.661G>A | p.Glu221Lys | NP_000153.1  (10694920) | NA | Brazil | LP |  |  |
|  | *GCK* | c.584T>C | p.Phe195Ser | NP_000153.1 | NA | China |  | 2018 | ([35](#_ENREF_35)) |
|  | *GCK* | c.632T>C | p.Met211Thr | NP_000153.1 | 3.97674400108167E-06 | China |  |  |  |
|  | *GCK* | c.665T>A | p.Val222Asp | NP_000153.1 | NA | China |  |  |  |
|  | *GCK* | c.707A>G | p.Glu236Gly | NP_000153.1 | NA | China |  |  |  |
|  | *GCK* | c.1373A>G | p.Lys458Arg | NP_000153.1 | NA | China |  |  |  |
|  | *GCK* | c.46-15_46-11del nsGGGAGGG |  | ? | NA | USA |  | 2010 | ([36](#_ENREF_36)) |
|  | *GCK* | c.45+1G>A |  | [NM_001354800.1](https://www.ncbi.nlm.nih.gov/nuccore/NM_001354800.1)  [NP_001341729.1](https://www.ncbi.nlm.nih.gov/nuccore/NP_001341729.1) | NA | Macedonia |  | 2017 | ([37](#_ENREF_37)) |
|  | *GCK* | c.895G>T | p.Gly299Cys | NP_000153.1  (1303265) | NA | UK |  | 1992 | ([38](#_ENREF_38)) |
|  | *GCK* | c.483G>C | p.Lys161Asn | [NM_001354800.1](https://www.ncbi.nlm.nih.gov/nuccore/NM_001354800.1)  [NP_001341729.1](https://www.ncbi.nlm.nih.gov/nuccore/NP_001341729.1)  (12955723) | NA | Italy | VUS | 2003 | ([39](#_ENREF_39)) |
|  | *GCK* | c.511T>C | p.Phe171Leu | [NM_001354800.1](https://www.ncbi.nlm.nih.gov/nuccore/NM_001354800.1)  [NP_001341729.1](https://www.ncbi.nlm.nih.gov/nuccore/NP_001341729.1) | NA | Italy | VUS |  |  |
|  | *GCK* | c.682A>G | p.Thr228Ala | [NM_001354800.1](https://www.ncbi.nlm.nih.gov/nuccore/NM_001354800.1)  [NP_001341729.1](https://www.ncbi.nlm.nih.gov/nuccore/NP_001341729.1)  (12955723) | NA | Italy | LP |  |  |
|  | *GCK* | c.683C>G | p.Thr228Arg, | [NM_001354800.1](https://www.ncbi.nlm.nih.gov/nuccore/NM_001354800.1)  [NP_001341729.1](https://www.ncbi.nlm.nih.gov/nuccore/NP_001341729.1)  (12955723) | NA | Italy | LP |  |  |
|  | *GCK* | c.772G>T | p.Gly258Cys | [NM_001354800.1](https://www.ncbi.nlm.nih.gov/nuccore/NM_001354800.1)  [NP_001341729.1](https://www.ncbi.nlm.nih.gov/nuccore/NP_001341729.1)  (12955723) | NA | Italy | VUS |  |  |
|  | *GCK* | c.1148C>A | p.Ser383Ter | [NM_001354800.1](https://www.ncbi.nlm.nih.gov/nuccore/NM_001354800.1)  [NP_001341729.1](https://www.ncbi.nlm.nih.gov/nuccore/NP_001341729.1)  (12955723) |  | Italy | P |  |  |
|  | *GCK* | c.1253+1G>T |  | [NM_001354800.1](https://www.ncbi.nlm.nih.gov/nuccore/NM_001354800.1)  [NP_001341729.1](https://www.ncbi.nlm.nih.gov/nuccore/NP_001341729.1) |  | Italy |  |  |  |
|  | *GCK* | c.1298_1309del12 | p.Ser433_Ile436del | NP_000153.1 |  | Italy |  |  |  |
|  | *GCK* | c.48_50delAGA, | p.Glu17del | [NM_001354800.1](https://www.ncbi.nlm.nih.gov/nuccore/NM_001354800.1)  [NP_001341729.1](https://www.ncbi.nlm.nih.gov/nuccore/NP_001341729.1) |  | Italy |  | 2017  ([40](#_ENREF_40)) |  |
|  | *GCK* | c.167A>G | p.Lys56Arg | [NM_001354800.1](https://www.ncbi.nlm.nih.gov/nuccore/NM_001354800.1)  [NP_001341729.1](https://www.ncbi.nlm.nih.gov/nuccore/NP_001341729.1) |  | Italy | VUS |  |  |
|  | *GCK* | c.208+1G>T |  | [NM_001354800.1](https://www.ncbi.nlm.nih.gov/nuccore/NM_001354800.1)  [NP_001341729.1](https://www.ncbi.nlm.nih.gov/nuccore/NP_001341729.1) |  | Italy |  |  |  |
|  | *GCK* | c.457C>T | p.Pro153Ser | [NM_001354800.1](https://www.ncbi.nlm.nih.gov/nuccore/NM_001354800.1)  [NP_001341729.1](https://www.ncbi.nlm.nih.gov/nuccore/NP_001341729.1) |  | Italy | LP |  |  |
|  | *GCK* | c.466C>A | p.His156Asn | [NM_001354800.1](https://www.ncbi.nlm.nih.gov/nuccore/NM_001354800.1) [NP_001341729.1](https://www.ncbi.nlm.nih.gov/nuccore/NP_001341729.1) |  | Italy | LP |  |  |
|  | *GCK* | c.475A>T | p.Ile159Phe | [NM_001354800.1](https://www.ncbi.nlm.nih.gov/nuccore/NM_001354800.1)  [NP_001341729.1](https://www.ncbi.nlm.nih.gov/nuccore/NP_001341729.1) |  | Italy | VUS |  |  |
|  | *GCK* | c.685G>T | p.Gly229Cys | [NM_001354800.1](https://www.ncbi.nlm.nih.gov/nuccore/NM_001354800.1) [NP_001341729.1](https://www.ncbi.nlm.nih.gov/nuccore/NP_001341729.1) |  | Italy | VUS |  |  |
|  | *GCK* | c.688T>G | p.Cys230Gly | [NM_001354800.1](https://www.ncbi.nlm.nih.gov/nuccore/NM_001354800.1)  [NP_001341729.1](https://www.ncbi.nlm.nih.gov/nuccore/NP_001341729.1) |  | Italy | LP |  |  |
|  | *GCK* | c.763A>C | p.Thr255Pro | [NM_001354800.1](https://www.ncbi.nlm.nih.gov/nuccore/NM_001354800.1)  [NP_001341729.1](https://www.ncbi.nlm.nih.gov/nuccore/NP_001341729.1) |  | Italy | VUS |  |  |
|  | *GCK* | c.775_777delGCC | p.Ala259del | NP_000153.1 | NA | Italy |  |  |  |
|  | *GCK* | c.859C>T | p. Gln287Ter | [NM_001354800.1](https://www.ncbi.nlm.nih.gov/nuccore/NM_001354800.1) [NP_001341729.1](https://www.ncbi.nlm.nih.gov/nuccore/NP_001341729.1) |  | Italy | P |  |  |
|  | *GCK* | c.925C>G | p.Leu309Val | [NM_001354800.1](https://www.ncbi.nlm.nih.gov/nuccore/NM_001354800.1)  [NP_001341729.1](https://www.ncbi.nlm.nih.gov/nuccore/NP_001341729.1) | NA | Italy | VUS |  |  |
|  | *GCK* | c.960_970del | p.Ala320del | NP_000153.1 | NA | Italy |  |  |  |
|  | *GCK* | c.1019G>A | p.Ser340Asn | [NM_001354800.1](https://www.ncbi.nlm.nih.gov/nuccore/NM_001354800.1)  [NP_001341729.1](https://www.ncbi.nlm.nih.gov/nuccore/NP_001341729.1) | NA | Italy | VUS |  |  |
|  | *GCK* | c.1180C>A | p.Arg394Ser | [NM_001354800.1](https://www.ncbi.nlm.nih.gov/nuccore/NM_001354800.1)  [NP_001341729.1](https://www.ncbi.nlm.nih.gov/nuccore/NP_001341729.1) |  | Italy | VUS |  |  |
|  | *GCK* | c.1182insA | p.Arg394ins | NP_000153.1 |  | Italy |  |  |  |
|  | *GCK* | c.1222G>A | p.Val408Met | [NM_001354800.1](https://www.ncbi.nlm.nih.gov/nuccore/NM_001354800.1) [NP_001341729.1](https://www.ncbi.nlm.nih.gov/nuccore/NP_001341729.1) |  | Italy | VUS |  |  |
|  | *GCK* | c.1228C>G | p.Gly410Arg | NP_000153.1 |  | Italy | VUS |  |  |
|  | *GCK* | c.1310C>T | p.Thr437Ile | [NM_001354800.1](https://www.ncbi.nlm.nih.gov/nuccore/NM_001354800.1)  [NP_001341729.1](https://www.ncbi.nlm.nih.gov/nuccore/NP_001341729.1) |  | Italy | VUS |  |  |
|  | *GCK* | c.1318G>A | p.Glu440Lys | [NM_001354800.1](https://www.ncbi.nlm.nih.gov/nuccore/NM_001354800.1)  [NP_001341729.1](https://www.ncbi.nlm.nih.gov/nuccore/NP_001341729.1) |  | Italy | VUS |  |  |
|  | *GCK* | c.1332_1333dupGC, | p.Gly444delins | NP_000153.1 |  | Italy |  |  |  |
|  | *GCK* | c.676G>A | p.Val226Met | NP_000153.1  (9049484) |  | Canada | LP | 2007 | ([41](#_ENREF_41)) |
|  | *GCK* | c.185T>C | p.Val62Ala | NP_000153.1  (9736233) |  | Norway | LP | 1998 | ([41](#_ENREF_41), [42](#_ENREF_42)) |
|  | *GCK* | c.509G > T | p.Gly170Val | NP_000153.1  (25372588) |  | Portugal | LP | 2014 | ([43](#_ENREF_43)) |
|  | *GCK* | c.980G>A | p.Arg327His | NP_000153.1  (18811724) |  | Thailand |  | 2009 | ([44](#_ENREF_44)) |
|  | *GCK* | c.208+1G>A |  | [NM_001354800.1](https://www.ncbi.nlm.nih.gov/nuccore/NM_001354800.1)  [NP_001341729.1](https://www.ncbi.nlm.nih.gov/nuccore/NP_001341729.1) |  | Czech Republic |  | 2010 | ([45](#_ENREF_45)) |
|  | *GCK* | c.242G>A | p.Gly81Asp | [NM_001354800.1](https://www.ncbi.nlm.nih.gov/nuccore/NM_001354800.1)  [NP_001341729.1](https://www.ncbi.nlm.nih.gov/nuccore/NP_001341729.1)  (20132997) |  | Czech Republic | VUS |  |  |
|  | *GCK* | c.370G>A, | p.Asp124Asn | [NM_001354800.1](https://www.ncbi.nlm.nih.gov/nuccore/NM_001354800.1)  [NP_001341729.1](https://www.ncbi.nlm.nih.gov/nuccore/NP_001341729.1)  (19790256) |  | Czech Republic | LP |  |  |
|  | *GCK* | c.540delT | p.Asn180fs | [NM_001354800.1](https://www.ncbi.nlm.nih.gov/nuccore/NM_001354800.1)  [NP_001341729.1](https://www.ncbi.nlm.nih.gov/nuccore/NP_001341729.1) |  | Czech Republic |  |  |  |
|  | *GCK* | c.579+1G>T |  | [NM_001354800.1](https://www.ncbi.nlm.nih.gov/nuccore/NM_001354800.1)  [NP_001341729.1](https://www.ncbi.nlm.nih.gov/nuccore/NP_001341729.1) | NA | Czech Republic |  |  |  |
|  | *GCK* | c.626C>A, | p.Thr209Lys | [NM_001354800.1](https://www.ncbi.nlm.nih.gov/nuccore/NM_001354800.1)  [NP_001341729.1](https://www.ncbi.nlm.nih.gov/nuccore/NP_001341729.1)  (20337973) |  | Czech Republic | VUS |  |  |
|  | *GCK* | c.677T>A, | p.Val226Glu | [NM_001354800.1](https://www.ncbi.nlm.nih.gov/nuccore/NM_001354800.1)  [NP_001341729.1](https://www.ncbi.nlm.nih.gov/nuccore/NP_001341729.1)  (20337973) |  | Czech Republic | LP |  |  |
|  | *GCK* | c.730delG, | p.Val244fs | [NM_001354800.1](https://www.ncbi.nlm.nih.gov/nuccore/NM_001354800.1)  [NP_001341729.1](https://www.ncbi.nlm.nih.gov/nuccore/NP_001341729.1) |  | Czech Republic |  |  |  |
|  | *GCK* | c.829 830delinsAG, | p.Val277Arg | [NM_001354800.1](https://www.ncbi.nlm.nih.gov/nuccore/NM_001354800.1)  [NP_001341729.1](https://www.ncbi.nlm.nih.gov/nuccore/NP_001341729.1) |  | Czech Republic |  |  |  |
|  | *GCK* | c.864-1G>C |  | [NM_001354800.1](https://www.ncbi.nlm.nih.gov/nuccore/NM_001354800.1)  [NP_001341729.1](https://www.ncbi.nlm.nih.gov/nuccore/NP_001341729.1)  (20337973) |  | Czech Republic |  |  |  |
|  | *GCK* | c.1153G>T | p.Gly385Trp | [NM_001354800.1](https://www.ncbi.nlm.nih.gov/nuccore/NM_001354800.1)  [NP_001341729.1](https://www.ncbi.nlm.nih.gov/nuccore/NP_001341729.1)  (20337973) |  | Czech Republic | LP |  |  |
|  | *GCK* | c.1233 1238delCinsACCCCA, | p.Val412fs | NP_000153.1 |  | Czech Republic |  |  |  |
|  | *GCK* | c.1334G>T | p.Glu442stop | [NM_001354800.1](https://www.ncbi.nlm.nih.gov/nuccore/NM_001354800.1)  [NP_001341729.1](https://www.ncbi.nlm.nih.gov/nuccore/NP_001341729.1)  (20337973) |  | Czech Republic | VUS |  |  |
|  | *GCK* |  | p. Leu77Arg | NP_000153.1  (27185633) |  | China |  | 2016 | ([46](#_ENREF_46)) |
|  | *GCK* | c.323A>G | p.Tyr108Cys | [NM_001354800.1](https://www.ncbi.nlm.nih.gov/nuccore/NM_001354800.1) [NP_001341729.1](https://www.ncbi.nlm.nih.gov/nuccore/NP_001341729.1)  (12050210) |  | Spain | VUS | 2002 | ([47](#_ENREF_47)) |
|  | *GCK* | c.563C>A  +564T>A, | 188A>E | [NM_001354800.1](https://www.ncbi.nlm.nih.gov/nuccore/NM_001354800.1)  [NP_001341729.1](https://www.ncbi.nlm.nih.gov/nuccore/NP_001341729.1) |  | Spain | LP |  |  |
|  | *GCK* | c.755G>A | p.Cys252Tyr | [NM_001354800.1](https://www.ncbi.nlm.nih.gov/nuccore/NM_001354800.1)  [NP_001341729.1](https://www.ncbi.nlm.nih.gov/nuccore/NP_001341729.1)  (12050210) |  | Spain | VUS |  |  |
|  | *GCK* | c.893T>A | p.Met298Lys | [NM_001354800.1](https://www.ncbi.nlm.nih.gov/nuccore/NM_001354800.1)  [NP_001341729.1](https://www.ncbi.nlm.nih.gov/nuccore/NP_001341729.1)  (12050210) |  | Spain | VUS |  |  |
|  | *GCK* | c.1106G>C | p.Arg369Pro | [NM_001354800.1](https://www.ncbi.nlm.nih.gov/nuccore/NM_001354800.1)  [NP_001341729.1](https://www.ncbi.nlm.nih.gov/nuccore/NP_001341729.1)  (12050210) |  | Spain | VUS |  |  |
|  | *GCK* | c.1148C>T | p.Ser383Leu | [NM_001354800.1](https://www.ncbi.nlm.nih.gov/nuccore/NM_001354800.1)  [NP_001341729.1](https://www.ncbi.nlm.nih.gov/nuccore/NP_001341729.1)  (11942313) |  | Spain | LP |  |  |
|  | *GCK* | c.1232C>T | p.Ser411Phe | [NM_001354800.1](https://www.ncbi.nlm.nih.gov/nuccore/NM_001354800.1)  [NP_001341729.1](https://www.ncbi.nlm.nih.gov/nuccore/NP_001341729.1)  (12050210) |  | Spain | VUS |  |  |
|  | *GCK* | c.-457C > T |  | [NM_001354800.1](https://www.ncbi.nlm.nih.gov/nuccore/NM_001354800.1)  [NP_001341729.1](https://www.ncbi.nlm.nih.gov/nuccore/NP_001341729.1) | 0.0000318 | Tunisia |  | 2018 | ([48](#_ENREF_48)) |
|  | *GCK* | c.227C>A | p.Ser76Tyr | NP_000153.1  (18399931) |  | Norway |  | \| 2008 \| \| --- \| \| | ([49](#_ENREF_49)) |
|  | *GCK* | c.692A>G | p.Asn231Ser | NP_000153.1  (18399931) | NA | Norway |  |  |  |
|  | *GCK* | c.110T>C | p.Met37Thr | [NM_001354800.1](https://www.ncbi.nlm.nih.gov/nuccore/NM_001354800.1)  [NP_001341729.1](https://www.ncbi.nlm.nih.gov/nuccore/NP_001341729.1) |  | Brazil | VUS | 2017 | ([50](#_ENREF_50)) |
|  | *GCK* | c.326delC | p.Ile110Serfs*6 | [NM_001354800.1](https://www.ncbi.nlm.nih.gov/nuccore/NM_001354800.1)  [NP_001341729.1](https://www.ncbi.nlm.nih.gov/nuccore/NP_001341729.1) |  | Brazil |  |  |  |
|  | *GCK* | c.418A>G | p.Lys140Glu | [NM_001354800.1](https://www.ncbi.nlm.nih.gov/nuccore/NM_001354800.1)  [NP_001341729.1](https://www.ncbi.nlm.nih.gov/nuccore/NP_001341729.1) |  | Brazil | VUS |  |  |
|  | *GCK* | c.505A>G | p.Lys169Glu | [NM_001354800.1](https://www.ncbi.nlm.nih.gov/nuccore/NM_001354800.1)  [NP_001341729.1](https://www.ncbi.nlm.nih.gov/nuccore/NP_001341729.1) | NA | Brazil | VUS |  |  |
|  | *GCK* | c.580-3C>A |  | [NM_001354800.1](https://www.ncbi.nlm.nih.gov/nuccore/NM_001354800.1)  [NP_001341729.1](https://www.ncbi.nlm.nih.gov/nuccore/NP_001341729.1) |  | Brazil |  |  |  |
|  | *GCK* | c.749G>A | p.Arg250His | [NM_001354800.1](https://www.ncbi.nlm.nih.gov/nuccore/NM_001354800.1)  [NP_001341729.1](https://www.ncbi.nlm.nih.gov/nuccore/NP_001341729.1) | NA | Brazil | VUS |  |  |
|  | *GCK* | c.767A>C | p.Glu256Ala | [NM_001354800.1](https://www.ncbi.nlm.nih.gov/nuccore/NM_001354800.1)  [NP_001341729.1](https://www.ncbi.nlm.nih.gov/nuccore/NP_001341729.1) | NA | Brazil | VUS |  |  |
|  | *GCK* | c.791G>A | p.Gly264Asp | [NM_001354800.1](https://www.ncbi.nlm.nih.gov/nuccore/NM_001354800.1)  [NP_001341729.1](https://www.ncbi.nlm.nih.gov/nuccore/NP_001341729.1) | NA | Brazil | VUS |  |  |
|  | *GCK* | c.1106_1111dup, | p.Cys371Serfs*33 | [NM_001354800.1](https://www.ncbi.nlm.nih.gov/nuccore/NM_001354800.1)  [NP_001341729.1](https://www.ncbi.nlm.nih.gov/nuccore/NP_001341729.1) | NA | Brazil |  |  |  |
|  | *GCK* | c.1135_1154del;1134_1135ins9, | p.His380GlyfsTer85 | [NM_001354800.1](https://www.ncbi.nlm.nih.gov/nuccore/NM_001354800.1)  [NP_001341729.1](https://www.ncbi.nlm.nih.gov/nuccore/NP_001341729.1) | NA | Brazil |  |  |  |
|  | *GCK* | c.1226delA | p.Asp409Valfs*22 | [NM_001354800.1](https://www.ncbi.nlm.nih.gov/nuccore/NM_001354800.1)  [NP_001341729.1](https://www.ncbi.nlm.nih.gov/nuccore/NP_001341729.1) | NA | Brazil |  |  |  |
|  | *GCK* | c.1247delA | p.His416Profs*15 | [NM_001354800.1](https://www.ncbi.nlm.nih.gov/nuccore/NM_001354800.1)  [NP_001341729.1](https://www.ncbi.nlm.nih.gov/nuccore/NP_001341729.1) | NA | Brazil |  |  |  |
|  | *GCK* | c.745G > T | p.G249C | [NM_001354800.1](https://www.ncbi.nlm.nih.gov/nuccore/NM_001354800.1)  [NP_001341729.1](https://www.ncbi.nlm.nih.gov/nuccore/NP_001341729.1)  (19790256) | NA | Turkey | VUS | 2016 | ([51](#_ENREF_51)) |
|  | *GCK* | c.91A > T | p.Lys31Ter | [NM_001354800.1](https://www.ncbi.nlm.nih.gov/nuccore/NM_001354800.1)  [NP_001341729.1](https://www.ncbi.nlm.nih.gov/nuccore/NP_001341729.1) | NA | Turkey | P |  |  |
|  | *GCK* | c.943C > T | p.Leu315Phe | [NM_001354800.1](https://www.ncbi.nlm.nih.gov/nuccore/NM_001354800.1)  [NP_001341729.1](https://www.ncbi.nlm.nih.gov/nuccore/NP_001341729.1)  (16965331) | NA | Turkey | VUS |  |  |
|  | *GCK* | c.112C > T | p.Gln38Ter | [NM_001354800.1](https://www.ncbi.nlm.nih.gov/nuccore/NM_001354800.1)  [NP_001341729.1](https://www.ncbi.nlm.nih.gov/nuccore/NP_001341729.1) | NA | Turkey | P |  |  |
|  | *GCK* | c.214G > A | p.Gly72Arg | [NM_001354800.1](https://www.ncbi.nlm.nih.gov/nuccore/NM_001354800.1)  [NP_001341729.1](https://www.ncbi.nlm.nih.gov/nuccore/NP_001341729.1)  (10447526) | NA | Turkey | LP |  |  |
|  | *GCK* | c.379T > C | p.Ser127Pro | [NM_001354800.1](https://www.ncbi.nlm.nih.gov/nuccore/NM_001354800.1) [NP_001341729.1](https://www.ncbi.nlm.nih.gov/nuccore/NP_001341729.1) | NA | Turkey | VUS |  |  |
|  | *GCK* | c. 1178T>C | p.Met393Thr | [NM_001354800.1](https://www.ncbi.nlm.nih.gov/nuccore/NM_001354800.1)  [NP_001341729.1](https://www.ncbi.nlm.nih.gov/nuccore/NP_001341729.1)  (19790256) | NA | Turkey | VUS |  |  |
|  | *GCK* | c.667G > A | [p.Gly223Ser](https://variantvalidator.org/service/validate/) | [NM_001354800.1](https://www.ncbi.nlm.nih.gov/nuccore/NM_001354800.1)  [NP_001341729.1](https://www.ncbi.nlm.nih.gov/nuccore/NP_001341729.1)  (11508276) |  | Turkey | LP |  |  |
|  | *GCK* | c.658T > C | p.Cys220Arg | [NM_001354800.1](https://www.ncbi.nlm.nih.gov/nuccore/NM_001354800.1)  [NP_001341729.1](https://www.ncbi.nlm.nih.gov/nuccore/NP_001341729.1)  (24097065) |  | Turkey | LP |  |  |
|  | *GCK* | c.616A > G | p.Thr206Ala | [NM_001354800.1](https://www.ncbi.nlm.nih.gov/nuccore/NM_001354800.1)  [NP_001341729.1](https://www.ncbi.nlm.nih.gov/nuccore/NP_001341729.1)  (24405491) | NA | India | LP | 2014 | ([52](#_ENREF_52)) |
|  | *GCK* |  | p.His156fs | NP_000153.1 | NA | Korea |  | 2019 | ([53](#_ENREF_53)) |
|  | *GCK* | c.490C>T | p.Leu164Phe | NP_000153.1  (18248649) |  | Korea |  |  |  |
|  | *GCK* | c.363+2T>C |  | [NM_001354800.1](https://www.ncbi.nlm.nih.gov/nuccore/NM_001354800.1)  [NP_001341729.1](https://www.ncbi.nlm.nih.gov/nuccore/NP_001341729.1) |  | Korea |  |  |  |
|  | *GCK* | c.1030G>T | p.Asp344Tyr | [NM_001354800.1](https://www.ncbi.nlm.nih.gov/nuccore/NM_001354800.1)  [NP_001341729.1](https://www.ncbi.nlm.nih.gov/nuccore/NP_001341729.1)  (27016322) |  | India | VUS | 2016 | ([54](#_ENREF_54)) |
|  | *GCK* | c.151G>T | p.Glu51Ter | [NM_001354800.1](https://www.ncbi.nlm.nih.gov/nuccore/NM_001354800.1)  [NP_001341729.1](https://www.ncbi.nlm.nih.gov/nuccore/NP_001341729.1) |  | Turkey | P | 2018 | ([55](#_ENREF_55)) |
|  | *GCK* | c.1396T>A | p.*466R | (19790256) |  | Turkey | VUS |  |  |
|  | *GCK* | c.595G>A | p.Val199Met | NP_000153.1 |  | Australia |  | 2019 | ([56](#_ENREF_56)) |
|  | *GCK* | c.206C> G | p.Ser69Stop | [NM_001354800.1](https://www.ncbi.nlm.nih.gov/nuccore/NM_001354800.1)  [NP_001341729.1](https://www.ncbi.nlm.nih.gov/nuccore/NP_001341729.1)  (23295287) |  | Brazil | P | 2012 | ([57](#_ENREF_57)) |
|  | *GCK* | c.431_432delTGinsCT L144P | p.Leu144Pro | [NM_001354800.1](https://www.ncbi.nlm.nih.gov/nuccore/NM_001354800.1)  [NP_001341729.1](https://www.ncbi.nlm.nih.gov/nuccore/NP_001341729.1) |  | Germany |  | 2005 | ([58](#_ENREF_58)) |
|  | *GCK* | c.580-1G>A |  | [NM_001354800.1](https://www.ncbi.nlm.nih.gov/nuccore/NM_001354800.1)  [NP_001341729.1](https://www.ncbi.nlm.nih.gov/nuccore/NP_001341729.1) |  | Germany |  |  |  |
|  | *GCK* | c.891C>A | p.Tyr297Ter | [NM_001354800.1](https://www.ncbi.nlm.nih.gov/nuccore/NM_001354800.1)  [NP_001341729.1](https://www.ncbi.nlm.nih.gov/nuccore/NP_001341729.1)  (15841481) |  | Germany | P |  |  |
|  | *GCK* | c.1195G>T | p.Glu399Ter | [NM_001354800.1](https://www.ncbi.nlm.nih.gov/nuccore/NM_001354800.1)  [NP_001341729.1](https://www.ncbi.nlm.nih.gov/nuccore/NP_001341729.1)  (15841481) |  | Germany | P |  |  |
|  | *GCK* | c.1377delG | K459fsX610 | NP_000153.1 | NA | Germany |  |  |  |
|  | *GCK* | c.73G>C | p.Ala25Pro | ? | NA | Germany | Uncertain |  |  |
|  | *GCK* | c.1155_1156insA | p.Leu386ThrfsTer86 | [NM_001354800.1](https://www.ncbi.nlm.nih.gov/nuccore/NM_001354800.1)  [NP_001341729.1](https://www.ncbi.nlm.nih.gov/nuccore/NP_001341729.1) |  | Germany |  |  |  |
|  | *GCK* | c.1323delG | p.Glu442ArgfsTer22 | [NM_001354800.1](https://www.ncbi.nlm.nih.gov/nuccore/NM_001354800.1)  [NP_001341729.1](https://www.ncbi.nlm.nih.gov/nuccore/NP_001341729.1) |  | Germany |  |  |  |
|  | *GCK* | c.157G>T | p.Ala53Ser | NP_000153.1 |  | France |  | 1997 | ([59](#_ENREF_59)) |
|  | *GCK* | c.239G>C | p.Gly80Ala | NP_000153.1  (9049484) |  | France |  |  |  |
|  | *GCK* | L122-1G >T |  | ? | NA | France |  |  |  |
|  | *GCK* | c.410A>G | p.His137Arg | NP_000153.1  (9049484) | NA | France |  |  |  |
|  | *GCK* | K161 + 1del10 |  | ? | NA | France |  |  |  |
|  | *GCK* | c.502A>C | p.Thr168Pro | NP_000153.1  (9049484) | NA | France |  |  |  |
|  | *GCK* |  | p.Arg186X | NP_000153.1 |  | France | P |  |  |
|  | *GCK* | c.629T>C | p.Met210Thr | NP_000153.1  (9049484) | NA | France |  |  |  |
|  | *GCK* | c.637T>C | p.Cys213Arg | NP_000153.1  (9049484) | NA | France |  |  |  |
|  | *GCK* | c.676G>A | p.Val226Met | NP_000153.1  (9049484) | NA | France | LP |  |  |
|  | *GCK* |  | p.Glu248X | NP_000153.1  (9049484) |  | France |  |  |  |
|  | *GCK* | c.781G>A | p.Gly261Arg | NP_000153.1  1502186 |  | France | LP |  |  |
|  | *GCK* | c.1007C>T | p.Ser336Leu | NP_000153.1  (9049484) |  | France |  |  |  |
|  | *GCK* | c.1079C>A | p.Ser360X | NP_000153.1  (9049484) |  | France |  |  |  |
|  | *GCK* | c.1099G>A | p.Val367Met | NP_000153.1  (9049484) |  | France |  |  |  |
|  | *GCK* | V401del1 |  | NP_000153.1 | NA | France |  |  |  |
|  | *GCK* | c.1257-20_1315del |  | [NM_001354800.1](https://www.ncbi.nlm.nih.gov/nuccore/NM_001354800.1)  [NP_001341729.1](https://www.ncbi.nlm.nih.gov/nuccore/NP_001341729.1) | NA | Korea |  | 2018 | ([60](#_ENREF_60)) |
|  | *GCK* | c.1084_1086del | p.Thr362del | [NM_001354800.1](https://www.ncbi.nlm.nih.gov/nuccore/NM_001354800.1)  [NP_001341729.1](https://www.ncbi.nlm.nih.gov/nuccore/NP_001341729.1) |  | China |  | 2019 | ([61](#_ENREF_61)) |
|  | *GCK* | c.1165delG | p.V389Sfs*13 9 | [NM_001354800.1](https://www.ncbi.nlm.nih.gov/nuccore/NM_001354800.1)  [NP_001341729.1](https://www.ncbi.nlm.nih.gov/nuccore/NP_001341729.1) |  | China |  |  |  |
|  | *GCK* | c.871A > T, | p.Lys291Ter | [NM_001354800.1](https://www.ncbi.nlm.nih.gov/nuccore/NM_001354800.1)  [NP_001341729.1](https://www.ncbi.nlm.nih.gov/nuccore/NP_001341729.1) |  | Germany |  | 2017 | ([62](#_ENREF_62)) |
|  | *GCK* | c.1340_1368del | p.Arg447LeufsTer15 | [NM_001354800.1](https://www.ncbi.nlm.nih.gov/nuccore/NM_001354800.1)  [NP_001341729.1](https://www.ncbi.nlm.nih.gov/nuccore/NP_001341729.1) |  | Germany |  |  |  |
|  | *GCK* | c.863+1G>T |  | ? | NA | Germany |  |  |  |
|  | *GCK* | c.484G>A, | p.Gly162Ser | [NM_001354800.1](https://www.ncbi.nlm.nih.gov/nuccore/NM_001354800.1)  [NP_001341729.1](https://www.ncbi.nlm.nih.gov/nuccore/NP_001341729.1)  (19790256) | NA | Germany | VUS |  |  |
|  | *GCK* | c.952G>A | p.Gly318Arg | [NM_001354800.1](https://www.ncbi.nlm.nih.gov/nuccore/NM_001354800.1)  [NP_001341729.1](https://www.ncbi.nlm.nih.gov/nuccore/NP_001341729.1)  (12627330) |  | Germany | LP |  |  |
|  | *GCK* | c.617C>T | p.Thr206Met | [NM_001354800.1](https://www.ncbi.nlm.nih.gov/nuccore/NM_001354800.1)  [NP_001341729.1](https://www.ncbi.nlm.nih.gov/nuccore/NP_001341729.1)  (11508276) |  | Germany | VUS |  |  |
|  | *GCK* | c.238G>A | p.Gly80Ser | [NM_001354800.1](https://www.ncbi.nlm.nih.gov/nuccore/NM_001354800.1)  [NP_001341729.1](https://www.ncbi.nlm.nih.gov/nuccore/NP_001341729.1)  (10694920) |  | Germany | LP |  |  |
|  | *GCK* | c.1349C > T | p.Ala450Val | [NM_001354800.1](https://www.ncbi.nlm.nih.gov/nuccore/NM_001354800.1)  [NP_001341729.1](https://www.ncbi.nlm.nih.gov/nuccore/NP_001341729.1)  (22035297) | 8.33270838020481E-06 | Germany | VUS |  |  |
|  | *GCK* | c.911T > C | p.Leu304Pro | [NM_001354800.1](https://www.ncbi.nlm.nih.gov/nuccore/NM_001354800.1)  [NP_001341729.1](https://www.ncbi.nlm.nih.gov/nuccore/NP_001341729.1)  (11942313) |  | Germany | LP |  |  |
|  | *GCK* | c.559G>T | p.Asp187Tyr | [NM_001354800.1](https://www.ncbi.nlm.nih.gov/nuccore/NM_001354800.1)  [NP_001341729.1](https://www.ncbi.nlm.nih.gov/nuccore/NP_001341729.1)  (17573900) |  | Germany | VUS |  |  |
|  | *GCK* | c.214G>A | p.Gly72Arg | [NM_001354800.1](https://www.ncbi.nlm.nih.gov/nuccore/NM_001354800.1)  [NP_001341729.1](https://www.ncbi.nlm.nih.gov/nuccore/NP_001341729.1)  (10447526) |  | Germany | LP |  |  |
|  | *GCK* | c.118G>A | p.Glu40Lys | [NM_001354800.1](https://www.ncbi.nlm.nih.gov/nuccore/NM_001354800.1)  [NP_001341729.1](https://www.ncbi.nlm.nih.gov/nuccore/NP_001341729.1) |  | Germany | LP |  |  |
|  | *GCK* | c.562G > A | p.Ala188Thr | [NM_001354800.1](https://www.ncbi.nlm.nih.gov/nuccore/NM_001354800.1)  [NP_001341729.1](https://www.ncbi.nlm.nih.gov/nuccore/NP_001341729.1)  (8314448) |  | Germany | VUS |  |  |
|  | *GCK* | c.640T>G | p.Tyr214Asp | [NM_001354800.1](https://www.ncbi.nlm.nih.gov/nuccore/NM_001354800.1)  [NP_001341729.1](https://www.ncbi.nlm.nih.gov/nuccore/NP_001341729.1)  (21348868) |  | Germany | VUS |  |  |
|  | *GCK* | c.131G > A | p.Gly44Asp | [NM_001354800.1](https://www.ncbi.nlm.nih.gov/nuccore/NM_001354800.1)  [NP_001341729.1](https://www.ncbi.nlm.nih.gov/nuccore/NP_001341729.1)  (12627330) | 3.97702868233085E-06 | Germany | LP |  |  |
|  | *GCK* | c.572G > A | p.Arg191Gln | [NM_001354800.1](https://www.ncbi.nlm.nih.gov/nuccore/NM_001354800.1)  [NP_001341729.1](https://www.ncbi.nlm.nih.gov/nuccore/NP_001341729.1)  (11508276) | NA | Germany | LP |  |  |
|  | *GCK* | c.787_801del | p.Ser263_Asp267del | [NM_001354800.1](https://www.ncbi.nlm.nih.gov/nuccore/NM_001354800.1)  [NP_001341729.1](https://www.ncbi.nlm.nih.gov/nuccore/NP_001341729.1) |  | Germany |  |  |  |
|  | *GCK* | c.544G>A | p.Val182Met | [NM_001354800.1](https://www.ncbi.nlm.nih.gov/nuccore/NM_001354800.1)  [NP_001341729.1](https://www.ncbi.nlm.nih.gov/nuccore/NP_001341729.1)  (8433729) |  | Germany | LP |  |  |
|  | *GCK* | c.706G>A | [p.Glu236Lys](https://variantvalidator.org/service/validate/) | [NM_001354800.1](https://www.ncbi.nlm.nih.gov/nuccore/NM_001354800.1)  [NP_001341729.1](https://www.ncbi.nlm.nih.gov/nuccore/NP_001341729.1)  (19790256) | NA | Germany | LP |  |  |
|  | *GCK* | c.394G>A | p.Asp132Asn | [NM_001354800.1](https://www.ncbi.nlm.nih.gov/nuccore/NM_001354800.1)  [NP_001341729.1](https://www.ncbi.nlm.nih.gov/nuccore/NP_001341729.1)  (18382660) | NA | Germany | VUS |  |  |
|  | *GCK* | c.757G>A, | p.Val253Ile | [NM_001354800.1](https://www.ncbi.nlm.nih.gov/nuccore/NM_001354800.1)  [NP_001341729.1](https://www.ncbi.nlm.nih.gov/nuccore/NP_001341729.1) |  | Germany | LP |  |  |
|  | *GCK* | c.31G > A | p.Ala11Thr | [NM_001354800.1](https://www.ncbi.nlm.nih.gov/nuccore/NM_001354800.1)  [NP_001341729.1](https://www.ncbi.nlm.nih.gov/nuccore/NP_001341729.1) | 0.0021 | Germany | likely benign |  |  |
|  | *GCK* | c.35A>G | p.Lys12Arg | [NM_001354800.1](https://www.ncbi.nlm.nih.gov/nuccore/NM_001354800.1)  [NP_001341729.1](https://www.ncbi.nlm.nih.gov/nuccore/NP_001341729.1) | 7.96996915621936E-06 | Germany | VUS |  |  |
|  | *GCK* |  | p.Arg43His | NP_000153.1  (11942313) |  | UK |  | 2012 | ([63](#_ENREF_63)) |
|  | *GCK* | c.203G>A | p.Gly68Asp | NP_000153.1  (19790256) |  | UK |  |  |  |
|  | *GCK* | c.208 + 3A>T |  | [NM_001354800.1](https://www.ncbi.nlm.nih.gov/nuccore/NM_001354800.1)  [NP_001341729.1](https://www.ncbi.nlm.nih.gov/nuccore/NP_001341729.1) | NA | Turkey |  | 2016 | ([64](#_ENREF_64)) |
|  | *GCK* | c.368T>C | p.Phe123Ser | [NM_001354800.1](https://www.ncbi.nlm.nih.gov/nuccore/NM_001354800.1)  [NP_001341729.1](https://www.ncbi.nlm.nih.gov/nuccore/NP_001341729.1) | NA | Turkey | VUS |  |  |
|  | *GCK* | c.173T>C | p.Leu58Pro | [NM_001354800.1](https://www.ncbi.nlm.nih.gov/nuccore/NM_001354800.1)  [NP_001341729.1](https://www.ncbi.nlm.nih.gov/nuccore/NP_001341729.1) |  | Turkey | VUS |  |  |
|  | *GCK* | c.1256T>G | p.Phe419Cys | [NM_001354800.1](https://www.ncbi.nlm.nih.gov/nuccore/NM_001354800.1)  [NP_001341729.1](https://www.ncbi.nlm.nih.gov/nuccore/NP_001341729.1) |  | Turkey |  |  |  |
|  | *GCK* | c.208+3A>T |  | [NM_001354800.1](https://www.ncbi.nlm.nih.gov/nuccore/NM_001354800.1)  [NP_001341729.1](https://www.ncbi.nlm.nih.gov/nuccore/NP_001341729.1) | NA | Turkey |  |  |  |
|  | *GCK* | c.452C>G | p.Ser151Cys | [NM_001354800.1](https://www.ncbi.nlm.nih.gov/nuccore/NM_001354800.1)  [NP_001341729.1](https://www.ncbi.nlm.nih.gov/nuccore/NP_001341729.1) | NA | Turkey |  |  |  |
|  | *GCK* | c.676G>A | p.Phe330Ser | NP_000153.1 | NA | Germany |  | 2011 | ([64](#_ENREF_64), [65](#_ENREF_65)) |
|  | *GCK* | c.120G>C | p.Glu40Asp | NP_000153.1  (22761713) | NA | Italy |  | 2012 | ([66](#_ENREF_66)) |
|  | *GCK* | c.460G>T | p.Val154Leu | NP_000153.1  (22761713) | NA | Italy |  |  |  |
|  | *GCK* | c.1339delC | p.Arg447Glyfs | NP_000153.1  (26587058) | NA | Italy |  |  |  |
|  | *GCK* | c.1373_1385del | p.Lys458_Cys461del | NP_000153.1 | NA | Italy |  |  |  |
|  | *GCK* |  | p.Glu395_Arg397del | NP_000153.1 | NA | Italy |  |  |  |
|  | *GCK* | c.580-2A>T |  | [NM_001354800.1](https://www.ncbi.nlm.nih.gov/nuccore/NM_001354800.1)  [NP_001341729.1](https://www.ncbi.nlm.nih.gov/nuccore/NP_001341729.1) | NA | Italy |  |  |  |
|  | *GCK* | c.431 T>C | p.Leu144Pro | [NM_001354800.1](https://www.ncbi.nlm.nih.gov/nuccore/NM_001354800.1)  [NP_001341729.1](https://www.ncbi.nlm.nih.gov/nuccore/NP_001341729.1) | NA | USA | VUS | 2016 | ([67](#_ENREF_67)) |
|  | *GCK* | c.460G>C | p.Val154Leu | [NM_001354800.1](https://www.ncbi.nlm.nih.gov/nuccore/NM_001354800.1)  [NP_001341729.1](https://www.ncbi.nlm.nih.gov/nuccore/NP_001341729.1)  (22761713) | NA | USA | VUS |  |  |
|  | *GCK* | c.605 T>C | p.Met202Thr | [NM_001354800.1](https://www.ncbi.nlm.nih.gov/nuccore/NM_001354800.1) [NP_001341729.1](https://www.ncbi.nlm.nih.gov/nuccore/NP_001341729.1)  (17937063) | NA | USA | LP |  |  |
|  | *GCK* | c.1160C>A | p.Ala387Glu | [NM_001354800.1](https://www.ncbi.nlm.nih.gov/nuccore/NM_001354800.1)  [NP_001341729.1](https://www.ncbi.nlm.nih.gov/nuccore/NP_001341729.1)  (23771925) | NA | USA | LP |  |  |
|  | *GCK* | c.1265G>C | p.Arg422Pro | [NM_001354800.1](https://www.ncbi.nlm.nih.gov/nuccore/NM_001354800.1)  [NP_001341729.1](https://www.ncbi.nlm.nih.gov/nuccore/NP_001341729.1) | NA | USA | VUS |  |  |
|  | *GCK* | c.554T > A | p.Leu185Gln | [NM_001354800.1](https://www.ncbi.nlm.nih.gov/nuccore/NM_001354800.1)  [NP_001341729.1](https://www.ncbi.nlm.nih.gov/nuccore/NP_001341729.1) | NA | Brazil | VUS | 2019 | ([68](#_ENREF_68)) |
|  | *GCK* | c.1334 G>C | p.Ser445Thr | [NM_001354800.1](https://www.ncbi.nlm.nih.gov/nuccore/NM_001354800.1)  [NP_001341729.1](https://www.ncbi.nlm.nih.gov/nuccore/NP_001341729.1) | NA | China | VUS | 2019 | ([69](#_ENREF_69)) |
|  | *GCK* | c.1289_1294delTGACGC | p.Leu430_Thr431del | [NM_001354800.1](https://www.ncbi.nlm.nih.gov/nuccore/NM_001354800.1)  [NP_001341729.1](https://www.ncbi.nlm.nih.gov/nuccore/NP_001341729.1) | NA | China |  |  |  |
|  | *GCK* | c.584 T>C | p.Phe195Ser | [NM_001354800.1](https://www.ncbi.nlm.nih.gov/nuccore/NM_001354800.1)  [NP_001341729.1](https://www.ncbi.nlm.nih.gov/nuccore/NP_001341729.1) | NA | China | VUS |  |  |
|  | *GCK* | c.30delC | p.Ala11ProfsTer6 | [NM_001354800.1](https://www.ncbi.nlm.nih.gov/nuccore/NM_001354800.1)  [NP_001341729.1](https://www.ncbi.nlm.nih.gov/nuccore/NP_001341729.1) | NA | China |  |  |  |
|  | *GCK* |  | p.Gly71Cys | ? | NA | UK |  | 2009 | ([70](#_ENREF_70)) |
|  | *GCK* | c.596T>C | p.Val199Ala | [NM_001354800.1](https://www.ncbi.nlm.nih.gov/nuccore/NM_001354800.1)  [NP_001341729.1](https://www.ncbi.nlm.nih.gov/nuccore/NP_001341729.1) | NA | Serbia |  | 2019 | ([71](#_ENREF_71)) |
|  | *GCK* | c.45G>A | p.Lys15= | [NM_001354800.1](https://www.ncbi.nlm.nih.gov/nuccore/NM_001354800.1)  [NP_001341729.1](https://www.ncbi.nlm.nih.gov/nuccore/NP_001341729.1)  (23771925) | NA | Italy |  | 2009 | ([72](#_ENREF_72)) |
|  | *GCK* | c.7G>A |  |  |  | Italy |  |  |  |
|  | *GCK* | c.451T>C | p.Ser151Pro | [NM_001354800.1](https://www.ncbi.nlm.nih.gov/nuccore/NM_001354800.1)  [NP_001341729.1](https://www.ncbi.nlm.nih.gov/nuccore/NP_001341729.1)  (19564454) | NA | Italy | VUS |  |  |
|  | *GCK* | c.483G>A | p.Lys161= | [NM_001354800.1](https://www.ncbi.nlm.nih.gov/nuccore/NM_001354800.1)  [NP_001341729.1](https://www.ncbi.nlm.nih.gov/nuccore/NP_001341729.1)  (12955723) | NA | Italy | VUS |  |  |
|  | *GCK* | c.613G>T | p.Asp205Tyr | [NM_001354800.1](https://www.ncbi.nlm.nih.gov/nuccore/NM_001354800.1)  [NP_001341729.1](https://www.ncbi.nlm.nih.gov/nuccore/NP_001341729.1)  (19564454) | NA | Italy | VUS |  |  |
|  | *GCK* | c.749G>C | p.Arg250Pro | [NM_001354800.1](https://www.ncbi.nlm.nih.gov/nuccore/NM_001354800.1)  [NP_001341729.1](https://www.ncbi.nlm.nih.gov/nuccore/NP_001341729.1)  (19564454) | NA | Italy | LP |  |  |
|  | *GCK* | c.794A>T | p.Glu265Val | [NM_001354800.1](https://www.ncbi.nlm.nih.gov/nuccore/NM_001354800.1)  [NP_001341729.1](https://www.ncbi.nlm.nih.gov/nuccore/NP_001341729.1)  (19564454) | NA | Italy | VUS |  |  |
|  | *GCK* | c.805T>C | p.Phe269Leu | [NM_001354800.1](https://www.ncbi.nlm.nih.gov/nuccore/NM_001354800.1)  [NP_001341729.1](https://www.ncbi.nlm.nih.gov/nuccore/NP_001341729.1)  (19564454) | NA | Italy | VUS |  |  |
|  | *GCK* | c.827T>C | p.Leu276Pro | [NM_001354800.1](https://www.ncbi.nlm.nih.gov/nuccore/NM_001354800.1) [NP_001341729.1](https://www.ncbi.nlm.nih.gov/nuccore/NP_001341729.1)  (19564454) | NA | Italy | VUS |  |  |
|  | *GCK* | c.865T>C | p.Tyr289His | [NM_001354800.1](https://www.ncbi.nlm.nih.gov/nuccore/NM_001354800.1) [NP_001341729.1](https://www.ncbi.nlm.nih.gov/nuccore/NP_001341729.1)  (19564454) | NA | Italy | VUS |  |  |
|  | *GCK* | c.976A>C | p.Thr326Pro | [NM_001354800.1](https://www.ncbi.nlm.nih.gov/nuccore/NM_001354800.1)  [NP_001341729.1](https://www.ncbi.nlm.nih.gov/nuccore/NP_001341729.1)  (19564454) | NA | Italy | VUS |  |  |
|  | *GCK* | c.991G>A | p.Glu331Lys | [NM_001354800.1](https://www.ncbi.nlm.nih.gov/nuccore/NM_001354800.1)  [NP_001341729.1](https://www.ncbi.nlm.nih.gov/nuccore/NP_001341729.1)  (19564454) | NA | Italy | VUS |  |  |
|  | *GCK* | c.1019+5G>A |  | [NM_001354800.1](https://www.ncbi.nlm.nih.gov/nuccore/NM_001354800.1)  [NP_001341729.1](https://www.ncbi.nlm.nih.gov/nuccore/NP_001341729.1) | NA | Italy |  |  |  |
|  | *GCK* | c.1313T>A | p.Phe438Tyr | [NM_001354800.1](https://www.ncbi.nlm.nih.gov/nuccore/NM_001354800.1)  [NP_001341729.1](https://www.ncbi.nlm.nih.gov/nuccore/NP_001341729.1)  (19564454) | NA | Italy | VUS |  |  |
|  | *GCK* | 33Val>Ala | p.Val33Ala | NP_000153.1  18271687 | NA | Czech Republic |  | 2008 | ([73](#_ENREF_73)) |
|  | *GCK gene* | 1343G >A, | p.Gly448Asp | [NM_001354800.1](https://www.ncbi.nlm.nih.gov/nuccore/NM_001354800.1)  [NP_001341729.1](https://www.ncbi.nlm.nih.gov/nuccore/NP_001341729.1) | NA | USA | VUS | 2018 | ([74](#_ENREF_74)) |
|  | *GCK* | 259A>T | p.Ala259Thr | NP_000153.1  (9662401) | NA | UK |  | 1998 | ([75](#_ENREF_75)) |
|  | *GCK* | c.1010delA | p.Gln337ArgfsTer16 | [NM_001354800.1](https://www.ncbi.nlm.nih.gov/nuccore/NM_001354800.1)  [NP_001341729.1](https://www.ncbi.nlm.nih.gov/nuccore/NP_001341729.1) | NA | Iran |  | 2013 | ([76](#_ENREF_76)) |
|  | *GCK* | c.44A>T | p.Lys15Met | [NM_001354800.1](https://www.ncbi.nlm.nih.gov/nuccore/NM_001354800.1)  [NP_001341729.1](https://www.ncbi.nlm.nih.gov/nuccore/NP_001341729.1)  (23771925) | NA | USA | VUS | 2013 | ([77](#_ENREF_77)) |
|  | *GCK* | c.1265G>T | p.Arg422Leu | [NM_001354800.1](https://www.ncbi.nlm.nih.gov/nuccore/NM_001354800.1)  [NP_001341729.1](https://www.ncbi.nlm.nih.gov/nuccore/NP_001341729.1)  (23771925) | NA | USA |  |  |  |
|  | *GCK* |  | p.Gly299Arg | NP_000153.1  (1303265) | NA | USA | LP | 2015 | ([78](#_ENREF_78)) |
|  | *GCK* |  | p.Val182Met | NP_000153.1  (8433729) |  | USA | LP |  |  |
|  | *GCK* | 185Arg>Ter | p.Arg185Ter | ? |  | USA |  |  |  |
|  | *GCK* | 340Arg>Lys | p.Arg340Lys | ? |  | USA |  |  |  |
|  | *GCK* | c.181T>G | p.Tyr61Asp | [NM_001354800.1](https://www.ncbi.nlm.nih.gov/nuccore/NM_001354800.1)  [NP_001341729.1](https://www.ncbi.nlm.nih.gov/nuccore/NP_001341729.1) |  | Brazil | VUS | 2019 | ([79](#_ENREF_79)) |
|  | *GCK* |  | p.Val62Ala | NP_000153.1  (9736233) |  | Norway |  | 2006 | ([80](#_ENREF_80)) |
|  | *GCK* |  | p.Gly72Arg | NP_000153.1  (10447526) |  | Norway |  |  |  |
|  | *GCK* |  | p.Lys146Arg | NP_000153.1  (16731834) |  | Norway |  |  |  |
|  | *GCK* |  | p.Ala208Thr | NP_000153.1  (16731834) |  | Norway |  |  |  |
|  | *GCK* |  | p.Met210Lys | NP_000153.1  (11372010) |  | Norway |  |  |  |
|  | *GCK* |  | p.Tyr215X | NP_000153.1  (10753050) |  | Norway |  |  |  |
|  | *GCK* |  | p.Ser263Pro | NP_000153.1  (12442280) |  | Norway |  |  |  |
|  | *GCK* |  | p.Glu339Gly | NP_000153.1  (16731834) |  | Norway |  |  |  |
|  | *GCK* |  | p.Arg377Cys | NP_000153.1  (16731834) |  | Norway |  |  |  |
|  | *GCK* |  | p.Ser453Leu | NP_000153.1  (14517956) |  | Norway | LP |  |  |
|  | *GCK* | IVS5 1G>C |  | ? |  | Norway |  |  |  |
|  | *GCK* | 203V>A | p.Val203Ala | NP_000153.1  (8433729) |  | Switzerland |  | 2005 | ([81](#_ENREF_81)) |
|  | *GCK* | c.244 A>G | p.Thr82Ala | [NM_001354800.1](https://www.ncbi.nlm.nih.gov/nuccore/NM_001354800.1)  [NP_001341729.1](https://www.ncbi.nlm.nih.gov/nuccore/NP_001341729.1)  (20587714) |  | Canada | VUS | 2010 | ([82](#_ENREF_82)) |
|  | *GCK* | c.83delA | p.Glu28GlyfsTer3 | [NM_001354800.1](https://www.ncbi.nlm.nih.gov/nuccore/NM_001354800.1)  [NP_001341729.1](https://www.ncbi.nlm.nih.gov/nuccore/NP_001341729.1) |  | Canada |  |  |  |
|  | *GCK* | c.1340G>A | p.Arg447Gln | [NM_001354800.1](https://www.ncbi.nlm.nih.gov/nuccore/NM_001354800.1)  [NP_001341729.1](https://www.ncbi.nlm.nih.gov/nuccore/NP_001341729.1)  (14517956) |  | UK | LP | 2016 | ([83](#_ENREF_83)) |
|  | *GCK* | c.659G>A, | p.Cys220Tyr | [NM_001354800.1](https://www.ncbi.nlm.nih.gov/nuccore/NM_001354800.1)  [NP_001341729.1](https://www.ncbi.nlm.nih.gov/nuccore/NP_001341729.1)  (22341229) |  | USA | LP | 2012 | ([84](#_ENREF_84)) |
|  | *GCK* |  | p.Glu70Asp | NP_000153.1  (18382660) |  | Italy |  | 2008 | ([85](#_ENREF_85)) |
|  | *GCK* |  | p.Phe123Leu | NP_000153.1  (18382660) |  | Italy |  |  |  |
|  | *GCK* |  | p.Asp132Asn | NP_000153.1 |  | Italy |  |  |  |
|  | *GCK* |  | p.His137Asp | NP_000153.1  (18382660) |  | Italy |  |  |  |
|  | *GCK* |  | p.Gly162Asp | NP_000153.1  (18382660) |  | Italy |  |  |  |
|  | *GCK* |  | p.Thr168Ala | NP_000153.1  (18382660) |  | Italy |  |  |  |
|  | *GCK* |  | p.Arg392Ser | NP_000153.1  (18382660) |  | Italy |  |  |  |
|  | *GCK* |  | p.Glu290X | NP_000153.1  (18382660) |  | Italy |  |  |  |
|  | *GCK* |  | p.Gln106_Met107delinsLeu | NP_000153.1 |  | Italy |  |  |  |
|  | *GCK* |  | p.Ile110N | NP_000153.1  22493702 |  | Slovakia |  | 2012 | ([86](#_ENREF_86)) |
|  | *GCK* |  | p.Val200Ala | NP_000153.1  (22493702) |  | Slovakia |  |  |  |
|  | *GCK* |  | p.Asn204Asp | NP_000153.1  (22493702) |  | Slovakia |  |  |  |
|  | *GCK* |  | p.Gly258Arg | NP_000153.1  (22493702) |  | Slovakia |  |  |  |
|  | *GCK* |  | p.Phe419Ser | NP_000153.1  (22493702) |  | Slovakia |  |  |  |
|  | *GCK* | c.580-A>C |  | [NM_001354800.1](https://www.ncbi.nlm.nih.gov/nuccore/NM_001354800.1)  [NP_001341729.1](https://www.ncbi.nlm.nih.gov/nuccore/NP_001341729.1) |  | Slovakia |  |  |  |
|  | *GCK* | c.1113–1114delGC |  | ? |  | Slovakia |  |  |  |
|  | *GCK* | c.507G>C | p.Lys169Asn | [NM_001354800.1](https://www.ncbi.nlm.nih.gov/nuccore/NM_001354800.1)  [NP_001341729.1](https://www.ncbi.nlm.nih.gov/nuccore/NP_001341729.1)  (11508276) | NA | China | VUS | 2019 | ([87](#_ENREF_87)) |
|  | *GCK* | c.645C>A | p.Tyr215Ter | [NM_001354800.1](https://www.ncbi.nlm.nih.gov/nuccore/NM_001354800.1)  [NP_001341729.1](https://www.ncbi.nlm.nih.gov/nuccore/NP_001341729.1)  (10753050) | NA | China | P |  |  |
|  | *GCK* | c.771G>A | p.Trp257Ter | [NM_001354800.1](https://www.ncbi.nlm.nih.gov/nuccore/NM_001354800.1)  [NP_001341729.1](https://www.ncbi.nlm.nih.gov/nuccore/NP_001341729.1) | NA | China | P |  |  |
|  | *GCK* | c.698G>A | p.Cys233Tyr | [NM_001354800.1](https://www.ncbi.nlm.nih.gov/nuccore/NM_001354800.1)  [NP_001341729.1](https://www.ncbi.nlm.nih.gov/nuccore/NP_001341729.1)  (24355479) | NA | NewZealand | Uncertain signficance | 2013 | ([88](#_ENREF_88)) |
|  | *GCK* | c.483+2T>A |  | [NM_001354800.1](https://www.ncbi.nlm.nih.gov/nuccore/NM_001354800.1)  [NP_001341729.1](https://www.ncbi.nlm.nih.gov/nuccore/NP_001341729.1) |  | China |  | 2018 | ([89](#_ENREF_89)) |
|  | *GCK* |  | p.Ser151del | NP_000153.1 |  | China |  |  |  |
|  | *GCK* |  | p.Met57GlyfsX29 | NP_000153.1 |  | China |  |  |  |
|  | *GCK* |  | p.Val374_Ala377del | NP_000153.1 |  | China |  |  |  |
|  | *HNF1a* |  | p.Val133Met | NP_001293108.2  (10754480) | NA | Spain |  | 2000 | ([3](#_ENREF_3)) |
|  | *HNF1a* |  | p.Arg159Gln | NP_001293108.2  (9097962) | NA | Spain |  |  |  |
|  | *HNF1a* |  | p.Val259Asp | NP_001293108.2  (10754480) | NA | Spain |  |  |  |
|  | *HNF1a* | c.1-326del |  | ? | NA | UK |  | 2019 | ([90](#_ENREF_90)) |
|  | *HNF1a* | c.404delA | p.Asp135ValfsTer20 | [NM_001306179.1](https://www.ncbi.nlm.nih.gov/nuccore/NM_001306179.1)  [NP_001293108.1](https://www.ncbi.nlm.nih.gov/nuccore/NP_001293108.1) | NA | UK |  |  |  |
|  | *HNF1a* | c.685C>T* | p.Arg229Ter | NP_001293108.2  (9032114) | 7.96355875513649E-06 | UK | P |  |  |
|  | *HNF1a* | c.779C>T | p.Thr260Met | NP_001293108.2  [NM_001306179.1](https://www.ncbi.nlm.nih.gov/nuccore/NM_001306179.1)  (9166684) | 4.01174639343999E-06 | UK | LP |  |  |
|  | *HNF1a* | c.872delC | p.Pro291GlnfsTer51 | [NM_001306179.1](https://www.ncbi.nlm.nih.gov/nuccore/NM_001306179.1) [[NP_001293108.1](https://www.ncbi.nlm.nih.gov/nuccore/NP_001293108.1)](https://www.ncbi.nlm.nih.gov/nuccore/NP_001293108.1)  (23771925) | NA | UK |  |  |  |
|  | *HNF1a* | c.872dup | p.Gly292ArgfsTer25 | NP_001293108.2  [NM_001306179.1](https://www.ncbi.nlm.nih.gov/nuccore/NM_001306179.1) | NA | UK |  |  |  |
|  | *HNF1a* | c.1129delC | p.Leu377fs | NP_001293108.2 [NM_001306179.1](https://www.ncbi.nlm.nih.gov/nuccore/NM_001306179.1) | NA | UK |  |  |  |
|  | *HNF1a* | c.1136_1137delCT† | p.Pro379ArgfsTer39 | [NM_001306179.1](https://www.ncbi.nlm.nih.gov/nuccore/NM_001306179.1)  [NP_001293108.1](https://www.ncbi.nlm.nih.gov/nuccore/NP_001293108.1) | NA | UK |  |  |  |
|  | *HNF1a* | c.1136C>G | p.Pro379Arg | NP_001293108.2  [NM_001306179.1](https://www.ncbi.nlm.nih.gov/nuccore/NM_001306179.1)  (15657605) | 3.99817683136489E-06 | UK | VUS |  |  |
|  | *HNF1a* | c.723C>A | p.Cys241Ter | NP_001293108.2  [NM_001306179.1](https://www.ncbi.nlm.nih.gov/nuccore/NM_001306179.1)  (26226118) | NA | Turkey | P | 2015 | ([5](#_ENREF_5)) |
|  | *HNF1a* | c.707G>A | p.Cys236tyr | NP_001293108.2 | NA | Canada | VUS | 2016 | ([7](#_ENREF_7)) |
|  | *HNF1a* | c.137_138insG | p.K46KfsX13 | NP_001293108.2 | NA | Canada |  |  |  |
|  | *HNF1a* | c.243_244 insAG | p.T82RfsX73 | NP_001293108.2 | NA | Canada |  |  |  |
|  | *HNF1a* | c.1108-3delTAG |  |  | NA | Canada |  |  |  |
|  | *HNF1a* |  | p.Glu48Asp | NP_001293108.2  (21168233) | NA | Argentina |  | 2011 | ([91](#_ENREF_91)) |
|  | *HNF1a* | g.914fsinsGA |  |  |  | Argentina |  |  |  |
|  | *HNF1a* | g.2707C>G | p.Arg203Gly | NP_001293108.2  (21168233) | NA | Argentina |  |  |  |
|  | *HNF1a* | g.3430delC |  | ? |  | Argentina |  |  |  |
|  | *HNF1a* |  | p.Gln463X | ? |  | Argentina |  |  |  |
|  | *HNF1a* | g.4962+3insT |  | ? |  | Argentina |  |  |  |
|  | *HNF1a* | g.1777-21c>t – |  | ? |  | Argentina |  |  |  |
|  | *HNF1a* | g.3513+23g>t |  | ? |  | Argentina |  |  |  |
|  | *HNF1a* | g.3482C>G | p.Pro308Pro | NP_001293108.2 |  | Argentina |  |  |  |
|  | *HNF1a* | g.4962+68a>g |  | ? |  | Argentina |  |  |  |
|  | *HNF1a* | c.802T>C | p.Phe268Leu | NP_001293108.2  [NM_001306179.1](https://www.ncbi.nlm.nih.gov/nuccore/NM_001306179.1)  (20132997) | NA | Czech Republic | VUS | 2010 | ([10](#_ENREF_10)) |
|  | *HNF1a* | c.871C>T | p.Pro291Ser | NP_001293108.2  [NM_001306179.1](https://www.ncbi.nlm.nih.gov/nuccore/NM_001306179.1)  (20132997) | 0.00023 | Czech Republic | Likely benign |  |  |
|  | *HNF1a* | c.92G>A | p.Gly31Asp | NP_001293108.2 [NM_001306179.1](https://www.ncbi.nlm.nih.gov/nuccore/NM_001306179.1)  (9754819) | 0.000749 | Netherland | LP | 2009 | ([92](#_ENREF_92)) |
|  | *HNF1A* | c.1501G>T | p.Ala501Ser | [NM_001306179.1](https://www.ncbi.nlm.nih.gov/nuccore/NM_001306179.1) [NP_001293108.1](https://www.ncbi.nlm.nih.gov/nuccore/NP_001293108.1)  (25041077) | NA | India | VUS | 2015 | ([93](#_ENREF_93)) |
|  | *HNf1a* | c. | p.Arg54X | NP_001293108.2  (12547858) | NA | China |  | 2015 | ([94](#_ENREF_94)) |
|  | *HNf1a* | c.92G>A | p.Gly31Asp | NP_001293108.2  (9754819) | 0.00074 | France |  | 1998 | ([95](#_ENREF_95)) |
|  | *HNf1a* | c.481G>A | p.Ala161Thr | NP_001293108.2  (9754819) | 0.0000954 | France |  |  |  |
|  | *HNf1a* |  | p.Arg200trp | NP_001293108.2  (9754819) | NA | France | VUS |  |  |
|  | *HNf1a* |  | p.Arg271Trp | NP_001293108.2  (9754819) | NA | France |  |  |  |
|  | *HNf1a* | IVS5nt+2T>A |  | ? | NA | France |  |  |  |
|  | *HNF1a* | P175delll |  | ? | NA | Israel |  | 2007 | ([15](#_ENREF_15)) |
|  | *HNF1a* | c.17G>A | p.Ser6Asn | NP_001293108.2  [NM_001306179.1](https://www.ncbi.nlm.nih.gov/nuccore/NM_001306179.1)  (18513305) | NA | Norway | VUS | 2008 | ([96](#_ENREF_96)) |
|  | *HNF1A* |  | p.Asn402Tyr | NP_001293108.2 | NA | Greece |  | 2020 | ([16](#_ENREF_16)) |
|  | *HNF1a* |  | p.Ile414Gly | NP_001293108.2 | NA | UK |  | 2000 | ([97](#_ENREF_97)) |
|  | *HNF1a* | c.1425insC | p.P475PfsX74 | NP_001293108.2 |  | Turkey |  | 2017 | ([98](#_ENREF_98)) |
|  | *HNF1a* | c.90_108delGGGTGAGCCGGGGCCCTACinsT | p.Gly31_Tyr36del | [NM_001306179.1](https://www.ncbi.nlm.nih.gov/nuccore/NM_001306179.1)  [NP_001293108.1](https://www.ncbi.nlm.nih.gov/nuccore/NP_001293108.1) |  | Turkey |  |  |  |
|  | *HNF1a* | c.-187C>A |  | [NM_001306179.1](https://www.ncbi.nlm.nih.gov/nuccore/NM_001306179.1)  [NP_001293108.1](https://www.ncbi.nlm.nih.gov/nuccore/NP_001293108.1) |  | Turkey | VUS |  |  |
|  | *HNF1a* | c.707G>A | p.Cys236Tyr | NP_001293108.2 [NM_001306179.1](https://www.ncbi.nlm.nih.gov/nuccore/NM_001306179.1) | NA | Ukraine |  | 2017 | ([99](#_ENREF_99)) |
|  | *HNF1a* | c.1621C>T | p.Gln541* | NP_001293108.2  [NM_001306179.1](https://www.ncbi.nlm.nih.gov/nuccore/NM_001306179.1) | NA | Ukraine | P |  |  |
|  | *HNF1a* | c.326+4A>G |  | [NM_001306179.1](https://www.ncbi.nlm.nih.gov/nuccore/NM_001306179.1) [NP_001293108.1](https://www.ncbi.nlm.nih.gov/nuccore/NP_001293108.1) |  | Ukraine |  |  |  |
|  | *HNF1a* |  | p.Pro224Ser | NP_001293108.2  (15031772) | NA | Germany |  | 2004 | ([100](#_ENREF_100)) |
|  | *HNF1a* |  | p.Gln9X | NP_001293108.2  (22432108) | NA | Japan |  | 2014 | ([101](#_ENREF_101)) |
|  | *HNF1a* |  | p.Leu12His | NP_001293108.2  (9287053) | NA | Japan |  |  |  |
|  | *HNF1a* |  | p.Ala15Asp | NP_001293108.2  (24905847) |  | Japan |  |  |  |
|  | *HNF1a* |  | p.Arg54X | NP_001293108.2  (12547858) |  | Japan | VUS |  |  |
|  | *HNF1a* |  | p.Arg131Trp | NP_001293108.2  (9166684) |  | Japan | VUS |  |  |
|  | *HNF1a* |  | p.His143Asn | NP_001293108.2  (24905847) |  | Japan |  |  |  |
|  | *HNF1a* |  | p.Lys158Asn | NP_001293108.2  (10078571) | NA | Japan |  |  |  |
|  | *HNF1a* |  | p.Arg159Gln | NP_001293108.2  (9097962) |  | Japan | LP |  |  |
|  | *HNF1a* | c.607C>T | p.Arg203Cys | NP_001293108.2  (10078571) | 0.00000397 | Japan | VUS |  |  |
|  | *HNF1a* | c.685C>T | p.Arg229X | NP_001293108.2  (9032114) | 7.96355875513649E-06 | Japan |  |  |  |
|  | *HNF1a* |  | p.Val233Leu | NP_001293108.2  (12488961) | NA | Japan |  |  |  |
|  | *HNF1a* | c.716C>T | p.Ala239Val | NP_001293108.2  (12488961) | 0.000172 | Japan | VUS |  |  |
|  | *HNF1a* |  | p.Arg271Gly | NP_001293108.2  (12050210) | NA | Japan |  |  |  |
|  | *HNF1a* |  | p.Arg271Trp | NP_001293108.2  (9754819) | NA | Japan | LP |  |  |
|  | *HNF1a* |  | p.Met283Arg | NP_001293108.2  (24905847) | NA | Japan |  |  |  |
|  | *HNF1a* |  | P291fsinsC | ? | NA | Japan |  |  |  |
|  | *HNF1a* | c.1340C>T | p.Pro447Leu | NP_001293108.2  (8945470) | 0.0000120 | Japan | LP |  |  |
|  | *HNF1a* |  | p.Pro499Leu | NP_001293108.2  (24905847) | NA | Japan |  |  |  |
|  | *HNF1a* | c.686G>A | p.Arg229Gln | NP_001293108.2  [NM_001306179.1](https://www.ncbi.nlm.nih.gov/nuccore/NM_001306179.1)  (9032114) | NA | Norway | LP | 2013 | ([22](#_ENREF_22)) |
|  | *HNF1a* | c.1136_1137delCT | p.Pro379fs | NP_001293108.2  [NM_001306179.1](https://www.ncbi.nlm.nih.gov/nuccore/NM_001306179.1) | NA | Norway |  |  |  |
|  | *HNF1a* | c.872dupC | p.Gly292fs | NP_001293108.2  [NM_001306179.1](https://www.ncbi.nlm.nih.gov/nuccore/NM_001306179.1) | NA | Norway |  |  |  |
|  | *HNF1a* | c.686G>A | p.Arg229Gln | NP_001293108.2  [NM_001306179.1](https://www.ncbi.nlm.nih.gov/nuccore/NM_001306179.1)  (9032114) | NA | Norway | LP |  |  |
|  | *HNF1a* | c.391C>T | p.Arg131Trp | NP_001293108.2 [NM_001306179.1](https://www.ncbi.nlm.nih.gov/nuccore/NM_001306179.1)  **(8945470)** | NA | Norway | VUS |  |  |
|  | *HNF1a* | c.686G>A | p.Arg229Gln | NP_001293108.2 [NM_001306179.1](https://www.ncbi.nlm.nih.gov/nuccore/NM_001306179.1)  **(23348805)** | NA | Norway | LP |  |  |
|  | *HNF1a* | c.1745A>G | p.His582Arg | ?  **(23348805)** | 0.0000540 | Norway | LP |  |  |
|  | *HNF1a* | c.872dupC | p.Gly292fs | NP_001293108.2v  [NM_001306179.1](https://www.ncbi.nlm.nih.gov/nuccore/NM_001306179.1) | NA | Norway |  |  |  |
|  | *HNF1a* | c.956-2A>G |  | [NM_001306179.1](https://www.ncbi.nlm.nih.gov/nuccore/NM_001306179.1) [NP_001293108.1](https://www.ncbi.nlm.nih.gov/nuccore/NP_001293108.1) | NA | Norway |  |  |  |
|  | *HNF1a* | c.1351A>G | p.Ser451Gly | NP_001293108.2  [NM_001306179.1](https://www.ncbi.nlm.nih.gov/nuccore/NM_001306179.1)  **23624530** | NA | Norway | VUS |  |  |
|  | *HNF1a* | c.666_668delGAA | p.Lys222del | NP_001293108.2  [NM_001306179.1](https://www.ncbi.nlm.nih.gov/nuccore/NM_001306179.1) | NA | Norway |  |  |  |
|  | *HNF1a* | c.593delA | p.Lys198fs | NP_001293108.2  [NM_001306179.1](https://www.ncbi.nlm.nih.gov/nuccore/NM_001306179.1) | NA | Japan |  | 2013 | ([102](#_ENREF_102)) |
|  | *HNF1a* | IVS5-1delTAG |  | ? | NA | Canada |  | 2004 | ([23](#_ENREF_23)) |
|  | *HNF1a* | E275fsdelGAAG |  | NP_001293108.2 | NA | Canada |  |  |  |
|  | *HNF1a* |  | p.Phe268Ser | NP_001293108.2  **(15305805)** | NA | Canada |  |  |  |
|  | *HNF1a* |  | p.Lys44fsdelC | NP_001293108.2 | NA | Canada |  |  |  |
|  | *HNF1a* | c.768delC | p.Asn257Tfs*85 | [NM_001306179.1](https://www.ncbi.nlm.nih.gov/nuccore/NM_001306179.1)  NP_001293108.2 | NA | USA |  | 2015 | ([25](#_ENREF_25)) |
|  | *HNF1a* |  | p.Tyr218Cys | NP_001293108.2  **(10872540)** | NA | China | VUS | 2000 | ([103](#_ENREF_103)) |
|  | *HNF1a* |  | P393fsdelC | ? | NA | South Korea |  | 2006 | ([27](#_ENREF_27)) |
|  | *HNF1a* | c.608G>A | p.Arg203His | NP_001293108.2  **(10588527)** | 7.95804518578056E-06 | Norway | LP | 2017 | ([104](#_ENREF_104)) |
|  | *HNF1a* | c.1061C>T | p.Thr354Met | NP_001293108.2  **(11058894)** | 0.00006365 | Norway | VUS |  |  |
|  | *HNF1a* | c.1640_1641del | p.Thr547ArgfsTer | ? |  | Norway |  |  |  |
|  | *HNF1a* | c.1739C>T | p.Pro580Leu | [NM_001306179.1](https://www.ncbi.nlm.nih.gov/nuccore/NM_001306179.1)  [NP_001293108.1](https://www.ncbi.nlm.nih.gov/nuccore/NP_001293108.1) | 0.0000166 | Norway | VUS |  |  |
|  | *HNF1a* | c.539C > T | p.Ala180Val | NP_001293108.2  [NM_001306179.1](https://www.ncbi.nlm.nih.gov/nuccore/NM_001306179.1)  **(24097065)** | NA | Norway | VUS | 2017 | ([105](#_ENREF_105)) |
|  | *HNF1a* |  | p.Gln495X | NP_001293108.2  **(21224407)** | NA | germany |  | 2013 | ([106](#_ENREF_106)) |
|  | *HNF1a* |  | p.Leu107Ile | NP_001293108.2  **(10447526)** |  | Sweden |  | 1999 | ([32](#_ENREF_32)) |
|  | *HNF1a* |  | p.Ser315fsinsA | NP_001293108.2 |  | Sweden |  |  |  |
|  | *HNF1a* |  | p.Gly375fsdelG | NP_001293108.2 |  | Sweden |  |  |  |
|  | *HNF1a* |  | p.Gly47Glu | NP_001293108.2  **(12574234)** |  | Norway |  | 2003 | ([107](#_ENREF_107)) |
|  | *HNF1a* | T196fsdelCCAA |  | NP_001293108.2 |  | Norway |  |  |  |
|  | *HNF1a* | IVS3–1G>A |  | ? |  | Norway |  |  |  |
|  | *HNF1a* | c.766T>A | p.Ser256Thr | NP_001293108.2  **(12574234)** | 0.0000200964 | Norway |  |  |  |
|  | *HNF1a* |  | p.Ala276Asp | NP_001293108.2  **(12574234)** |  | Norway | LP |  |  |
|  | *HNF1a* |  | p.S445fsdelAG | NP_001293108.2 |  | Norway |  |  |  |
|  | *HNF1a* | c.1564A>G | p.Met522Val | NP_001293108.2  **(12574234)** | 3.97933927050752E-06 | Norway |  |  |  |
|  | *HNF1a* | c.1592G>C | p.Ser531Thr | NP_001293108.2  **(12574234)** | 0.00001990 | Norway |  |  |  |
|  | *HNF1a* | c.-154_-160TGGGGGT |  | ? | NA | Spain |  | 2013 | ([108](#_ENREF_108)) |
|  | *HNF1a* | c.368T > C | p.Leu123Pro | NP_001293108.2  **(18433912)** | NA | Serbia | VUS | 2008 | ([109](#_ENREF_109)) |
|  | *HNF1a* | c.392G>A | p.Arg131Gln | NP_001293108.2  **(8945470)** | 0.000003977882 | Japan | LP | 2016 | ([110](#_ENREF_110)) |
|  | *HNF1a* |  | p.Arg203Ser | NP_001293108.2  **(23348805)** | NA | Japan |  |  |  |
|  | *HNF1a* | c.226G>A | p.Asp76Asn | NP_001293108.2  [NM_001306179.1](https://www.ncbi.nlm.nih.gov/nuccore/NM_001306179.1) | NA | Italy | VUS | 2017 | ([40](#_ENREF_40)) |
|  | *HNF1a* | c.1182insA | p.Pro394ins | NP_001293108.2 | NA | Italy |  |  |  |
|  | *HNF1a* | c.1053delG | p.Ser352fs | NP_001293108.2 [NM_001306179.1](https://www.ncbi.nlm.nih.gov/nuccore/NM_001306179.1) | NA | Ireland |  | 2011 | ([111](#_ENREF_111)) |
|  | *HNF1a* | c.1276 1277insAGGT | p.Phe426X | NP_001293108.2 | NA | Ireland |  |  |  |
|  | *HNF1a* |  | p.Arg229Gln | NP_001293108.2  **(9032114)** | NA | germany |  | 1997 | ([112](#_ENREF_112)) |
|  | *HNF1a* |  | p.Arg229X | NP_001293108.2  **(9032114)** | NA | germany | LP |  |  |
|  | *HNF1a* | c.241C>G |  | NP_001293108.2  **(9032114)** | NA | germany |  |  |  |
|  | *HNF1a* |  | p.Arg272His | NP_001293108.2  **(9032114)** | NA | germany | LP |  |  |
|  | *HNF1a* |  | P291fsinsC | NP_001293108.2 | NA | germany |  |  |  |
|  | *HNF1a* |  | p.Gln401fsdelC | NP_001293108.2 | NA | germany |  |  |  |
|  | *HNF1a* | c.1424C>T | p.Pro475Leu | NP_001293108.2 | 0.000052021 | Thailand |  | 2009 | ([44](#_ENREF_44)) |
|  | *HNF1a* |  | p.Gly554fsX556 | ? | NA | Thailand |  |  |  |
|  | *HNF1a* | c.811C>G | p.Arg271Gly | NP_001293108.2  **(18811724)** | NA | Spain | LP | 2002 | ([47](#_ENREF_47)) |
|  | *HNF1a* |  | p.His153N | [NM_001306179.1](https://www.ncbi.nlm.nih.gov/nuccore/NM_001306179.1)  [NP_001293108.1](https://www.ncbi.nlm.nih.gov/nuccore/NP_001293108.1)  [p.(Gly51=)](https://variantvalidator.org/service/validate/) | NA | Japan |  | 2004 | ([113](#_ENREF_113)) |
|  | *HNF1a* | c.1558C>T | p.Gln520Ter | NP_001293108.2 [NM_001306179.1](https://www.ncbi.nlm.nih.gov/nuccore/NM_001306179.1) |  | Brazil | P | 2017 | ([50](#_ENREF_50)) |
|  | *HNF1a* | c.1722_1740dup |  | ? |  | Brazil |  |  |  |
|  | *HNF1a* | c.1192C > T | p.Q398* | NP_001293108.2 [NM_001306179.1](https://www.ncbi.nlm.nih.gov/nuccore/NM_001306179.1)  **(21224407)** |  | Turkey | P | 2016 | ([51](#_ENREF_51)) |
|  | *HNF1a* |  | p.Arg272His | NP_001293108.2  **(9032114)** |  | Japan | LP | 1997 | ([114](#_ENREF_114)) |
|  | *HNF1a* | P291fsinsC |  | NP_001293108.2 |  | Japan |  |  |  |
|  | *HNF1a* | c.1747C>G | p.Arg583Gly | ?  **(9313763)** | 4.16347466942011E-06 | Japan |  |  |  |
|  | *HNF1a* | c.415C>G | p.Leu139Val | NP_001293108.2 [NM_001306179.1](https://www.ncbi.nlm.nih.gov/nuccore/NM_001306179.1) | NA | Korea | VUS | 2016 | ([115](#_ENREF_115)) |
|  | *HNF1a* | P291fsinsC |  | NP_001293108.2 | NA | Tunisia |  | 2016 | ([116](#_ENREF_116)) |
|  | *HNF1a* | c.1340 C>T | p.Pro447Leu | NP_001293108.2 [NM_001306179.1](https://www.ncbi.nlm.nih.gov/nuccore/NM_001306179.1)  **(8945470)** | 0.0000120 | USA | LP | 2016 | ([117](#_ENREF_117)) |
|  | *HNF1a* | 619E>K | p.Glu619Lys | NP_001293108.2  **(9626139)** | NA | USA |  | 1998 | ([118](#_ENREF_118)) |
|  | *HNF1a* |  | p.Arg537Thr | ?  **(9626139)** |  | USA |  |  |  |
|  | *HNF1a* | c.712A>G | p.Arg238Gly | NP_001293108.2 [NM_001306179.1](https://www.ncbi.nlm.nih.gov/nuccore/NM_001306179.1) |  | Turkey | VUS | 2018 | ([55](#_ENREF_55)) |
|  | *HNF1a* | c.1831_1838delCCG GCCCA | p.H613Qfs*64 | ? |  | Turkey |  |  |  |
|  | *HNF1a* | c.608G>A | p.Arg203His | NP_001293108.2  **(10588527)** | 7.95804518578056E-06 | Japan |  | 1998 | ([119](#_ENREF_119)) |
|  | *HNF1a* | c.481G>C | p.Ala161Pro | [NM_001306179.1](https://www.ncbi.nlm.nih.gov/nuccore/NM_001306179.1)  NP_001293108.2  **(23517481)** |  | Greece | VUS | 2013 | ([120](#_ENREF_120)) |
|  | *HNF1a* | c.493T>C | p.Trp165Arg* | NP_001293108.2 [NM_001306179.1](https://www.ncbi.nlm.nih.gov/nuccore/NM_001306179.1)  **(23517481)** |  | Greece | VUS |  |  |
|  | *HNF1a* | c.682-684delGAG | p.Glu228del | NP_001293108.2 |  | Greece |  |  |  |
|  | *HNF1a* | c.1177delT | p.Ser393fs | NP_001293108.2  [NM_001306179.1](https://www.ncbi.nlm.nih.gov/nuccore/NM_001306179.1) |  | Greece |  |  |  |
|  | *HNF1a* | c.1201C>T | p.Gln401X | NP_001293108.2  **(23517481)** |  | Greece | P |  |  |
|  | *HNF1a* | c.1331-1332 delAG | p.Ser445fs | NP_001293108.2 | NA | Greece |  |  |  |
|  | *HNF1a* |  | p.Pro379fsdelCT | NP_001293108.2 | NA | UK |  | 1997 | ([112](#_ENREF_112)) |
|  | *HNF1a* |  | p.A443fsdelCA | NP_001293108.2 | NA | UK |  |  |  |
|  | *HNF1a* |  | p.Pro129T | NP_001293108.2  **(9075818)** | NA | UK |  |  |  |
|  | *HNF1a* |  | p.Arg131Trp | NP_001293108.2  **(9166684)** |  | UK |  |  |  |
|  | *HNF1a* | c.475C>T | p.Arg159Trp | NP_001293108.2  **(9754819)** | 3.97807286238254E-06 | UK | LP |  |  |
|  | *HNF1a* |  | p.Pro519Leu | NP_001293108.2  **(9075818)** | NA | UK |  |  |  |
|  | *HNF1a* |  | p.Thr620Ile | ?  **(9075818)** | NA | UK |  |  |  |
|  | *HNF1a* |  | p.Ile128Asn | NP_001293108.2  **(9075819)** |  | USA |  | 1997 | ([121](#_ENREF_121)) |
|  | *HNF1a* |  | p.His143Tyr | NP_001293108.2  **(9075819)** |  | USA |  |  |  |
|  | *HNF1a* |  | p.Pro447Lys | NP_001293108.2  **(8945470)** |  | USA |  |  |  |
|  | *HNF1a* |  | p.Pro379fsdelT | NP_001293108.2 |  | USA |  |  |  |
|  | *HNF1a* |  | p.Ala559fsinsA | ? |  | USA |  |  |  |
|  | *HNF1a* | c.-538G>C | p.Ala180Pro | [NM_001306179.1](https://www.ncbi.nlm.nih.gov/nuccore/NM_001306179.1)  [NP_001293108.1](https://www.ncbi.nlm.nih.gov/nuccore/NP_001293108.1) |  | India | VUS | 2009 | ([122](#_ENREF_122)) |
|  | *HNF1a* | c.340C>T | p.Arg114Cys | NP_001293108.2  [NM_001306179.1](https://www.ncbi.nlm.nih.gov/nuccore/NM_001306179.1)  **(19336507)** | 0.0000119 | India | VUS |  |  |
|  | *HNF1a* | c.402C>T | p.Val134Val | NP_001293108.2 [NM_001306179.1](https://www.ncbi.nlm.nih.gov/nuccore/NM_001306179.1)  **(19336507)** | 0.0000119 | India | Likely benign |  |  |
|  | *HNF1a* | c.511C>G | p.Arg171Gly | NP_001293108.2 [NM_001306179.1](https://www.ncbi.nlm.nih.gov/nuccore/NM_001306179.1)  **(19336507)** |  | India | VUS |  |  |
|  | *HNF1a* | c.703G>C | p.Glu235Gln | NP_001293108.2  [NM_001306179.1](https://www.ncbi.nlm.nih.gov/nuccore/NM_001306179.1)  **(19336507)** | 3.98787685436273E-06 | India | VUS |  |  |
|  | *HNF1a* | c.733G>A | p.Gly245Arg | NP_001293108.2 [NM_001306179.1](https://www.ncbi.nlm.nih.gov/nuccore/NM_001306179.1)  **(19336507)** | NA | India | VUS |  |  |
|  | *HNF1a* | c.788G>A | p.Arg263His | NP_001293108.2  [NM_001306179.1](https://www.ncbi.nlm.nih.gov/nuccore/NM_001306179.1)  **(16917892)** | NA | India | LP |  |  |
|  | *HNF1a* | c.864_865insG | p.Pro289AlafsTer28 | [NM_001306179.1](https://www.ncbi.nlm.nih.gov/nuccore/NM_001306179.1)  [NP_001293108.1](https://www.ncbi.nlm.nih.gov/nuccore/NP_001293108.1) | NA | China |  | 2019 | ([123](#_ENREF_123)) |
|  | *HNF1a* | c.245C>T | p.Thr82Met | NP_001293108.2  [NM_001306179.1](https://www.ncbi.nlm.nih.gov/nuccore/NM_001306179.1)  **(16834925)** | 0.00004836 | China | VUS | 2006 | ([124](#_ENREF_124)) |
|  | *HNF1a* | c.390G>T | p.Gln130His | NP_001293108.2  [NM_001306179.1](https://www.ncbi.nlm.nih.gov/nuccore/NM_001306179.1)  **(16834925)** | NA | China | VUS |  |  |
|  | *HNF1a* |  | p.Pro353fsdelACGGGCCTGGAGC | ? | NA | China |  |  |  |
|  | *HNF1a* | c.442C>T | p.Leu148Phe | NP_001293108.2 | NA | Russia | VUS | 2019 | ([125](#_ENREF_125)) |
|  | *HNF1a* |  | p.Ser6Arg | NP_001293108.2 | NA | Russia |  | 2018 | ([126](#_ENREF_126)) |
|  | *HNF1a* | c.994delG | p.Glu332fs | NP_001293108.2 | NA | Germany |  | 2017 | ([62](#_ENREF_62)) |
|  | *HNF1a* | c.955+1G>T |  | [NM_001306179.1](https://www.ncbi.nlm.nih.gov/nuccore/NM_001306179.1)  [NP_001293108.1](https://www.ncbi.nlm.nih.gov/nuccore/NP_001293108.1) | NA | Germany |  |  |  |
|  | *HNF1a* | c.1730_1733dupACCT | p.Gln579fs | ? | NA | Germany |  |  |  |
|  | *HNF1a* | c.1A>C | p.Met1Leu | [NM_001306179.1](https://www.ncbi.nlm.nih.gov/nuccore/NM_001306179.1)  NP_001293108.2  **(18003757)** | NA | France | LP | 2008 | ([127](#_ENREF_127)) |
|  | *HNF1a* | c.22C>A | p.Leu8Met | NP_001293108.2  [NM_001306179.1](https://www.ncbi.nlm.nih.gov/nuccore/NM_001306179.1)  **(18003757)** | NA | France | VUS |  |  |
|  | *HNF1a* | c.41C>T | p.Ala14Val | NP_001293108.2 [NM_001306179.1](https://www.ncbi.nlm.nih.gov/nuccore/NM_001306179.1)  **(18003757)** | NA | France | VUS |  |  |
|  | *HNF1a* | c.49C>G | p.Leu17Val | NP_001293108.2  [NM_001306179.1](https://www.ncbi.nlm.nih.gov/nuccore/NM_001306179.1)  **(18003757)** | NA | France | VUS |  |  |
|  | *HNF1a* | c.50T>A | p.Leu17Gln | NP_001293108.2  [NM_001306179.1](https://www.ncbi.nlm.nih.gov/nuccore/NM_001306179.1) | NA | France | VUS |  |  |
|  | *HNF1a* | c.59G>C | p.Gly20Ala | NP_001293108.2  [NM_001306179.1](https://www.ncbi.nlm.nih.gov/nuccore/NM_001306179.1)  **(18003757)** | NA | France | LP |  |  |
|  | *HNF1a* | c.77T>C | p.Leu26Pro | NP_001293108.2  [NM_001306179.1](https://www.ncbi.nlm.nih.gov/nuccore/NM_001306179.1)  **(18003757)** | NA | France | VUS |  |  |
|  | *HNF1a* | c.80T>C | p.Ile27Thr | NP_001293108.2 [NM_001306179.1](https://www.ncbi.nlm.nih.gov/nuccore/NM_001306179.1)  **(18003757)** | NA | France | VUS |  |  |
|  | *HNF1a* | c.80T>G | p.Ile27Ser | NP_001293108.2  [NM_001306179.1](https://www.ncbi.nlm.nih.gov/nuccore/NM_001306179.1)  **(18003757)** | NA | France | VUS |  |  |
|  | *HNF1a* | c.82C>T | p.Gln28X | NP_001293108.2 [NM_001306179.1](https://www.ncbi.nlm.nih.gov/nuccore/NM_001306179.1) | NA | France | VUS |  |  |
|  | *HNF1a* | c.98C>T | p.Pro33Leu | NP_001293108.2  [NM_001306179.1](https://www.ncbi.nlm.nih.gov/nuccore/NM_001306179.1)  **(18003757)** |  | France | VUS |  |  |
|  | *HNF1a* | c.202C>T | p.Arg68Trp | NP_001293108.2 [NM_001306179.1](https://www.ncbi.nlm.nih.gov/nuccore/NM_001306179.1)  **(18003757)** | 4.32271674101739E-06 | France | VUS |  |  |
|  | *HNF1a* | c.206delG | p.Gly69fs | NP_001293108.2  [NM_001306179.1](https://www.ncbi.nlm.nih.gov/nuccore/NM_001306179.1) | NA | France |  |  |  |
|  | *HNF1a* | c.217G>T | p.Glu73Ter | NP_001293108.2  [NM_001306179.1](https://www.ncbi.nlm.nih.gov/nuccore/NM_001306179.1)  **(18003757)** | NA | France | VUS |  |  |
|  | *HNF1a* | c.225C>A | p.Asp75Glu | NP_001293108.2 [NM_001306179.1](https://www.ncbi.nlm.nih.gov/nuccore/NM_001306179.1)  **(18003757)** | 4.25311115080681E-06 | France | VUS |  |  |
|  | *HNF1a* | c.259A>T | p.Lys87X | NP_001293108.2 [NM_001306179.1](https://www.ncbi.nlm.nih.gov/nuccore/NM_001306179.1)  **(18003757)** | NA | France | VUS |  |  |
|  | *HNF1a* | c.282_283insT | p.Glu95X | NP_001293108.2  [NM_001306179.1](https://www.ncbi.nlm.nih.gov/nuccore/NM_001306179.1) | NA | France |  |  |  |
|  | *HNF1a* | c.319C>G | p.Leu107Val | NP_001293108.2 [NM_001306179.1](https://www.ncbi.nlm.nih.gov/nuccore/NM_001306179.1)  **(18003757)** | NA | France | VUS |  |  |
|  | *HNF1a* | c.326delA | p.Gln109fs | NP_001293108.2  [NM_001306179.1](https://www.ncbi.nlm.nih.gov/nuccore/NM_001306179.1) | NA | France |  |  |  |
|  | *HNF1a* | c.346G>A | p.Ala116Thr | NP_001293108.2 [NM_001306179.1](https://www.ncbi.nlm.nih.gov/nuccore/NM_001306179.1)  **(18003757)** | NA | France | VUS |  |  |
|  | *HNF1a* | c.368T>G | p.Leu123Arg | NP_001293108.2 [NM_001306179.1](https://www.ncbi.nlm.nih.gov/nuccore/NM_001306179.1)  **(18003757)** | NA | France | VUS |  |  |
|  | *HNF1a* | c.396G>C | p.Glu132Asp | NP_001293108.2 [NM_001306179.1](https://www.ncbi.nlm.nih.gov/nuccore/NM_001306179.1)  **(18003757)** | NA | France | VUS |  |  |
|  | *HNF1a* | c.397G>T | p.Val133Leu | NP_001293108.2 [NM_001306179.1](https://www.ncbi.nlm.nih.gov/nuccore/NM_001306179.1)  **(18003757)** | NA | France | VUS |  |  |
|  | *HNF1a* | c.403G>A | p.Asp135Asn | NP_001293108.2 [NM_001306179.1](https://www.ncbi.nlm.nih.gov/nuccore/NM_001306179.1)  **(18003757)** | NA | France | VUS |  |  |
|  | *HNF1a* | c.410C>G | p.Thr137Ser | NP_001293108.2  [NM_001306179.1](https://www.ncbi.nlm.nih.gov/nuccore/NM_001306179.1)  **(18003757)** | NA | France | VUS |  |  |
|  | *HNF1a* | c.412G>A | p.Gly138Ser | NP_001293108.2  [NM_001306179.1](https://www.ncbi.nlm.nih.gov/nuccore/NM_001306179.1)  **(18003757)** | NA | France | VUS |  |  |
|  | *HNF1a* | c.427delC | p.His143fs | NP_001293108.2  [NM_001306179.1](https://www.ncbi.nlm.nih.gov/nuccore/NM_001306179.1) | NA | France |  |  |  |
|  | *HNF1a* | c.436_438dup | p.Gln146dup | NP_001293108.2 [NM_001306179.1](https://www.ncbi.nlm.nih.gov/nuccore/NM_001306179.1) | NA | France |  |  |  |
|  | *HNF1a* | c.436C>T | p.Gln146X | NP_001293108.2 [NM_001306179.1](https://www.ncbi.nlm.nih.gov/nuccore/NM_001306179.1)  **(18003757)** | NA | France | P |  |  |
|  | *HNF1a* | c.442C>A | p.Leu148Ile | NP_001293108.2 [NM_001306179.1](https://www.ncbi.nlm.nih.gov/nuccore/NM_001306179.1)  **(18003757)** | NA | France | VUS |  |  |
|  | *HNF1a* | c.447C>G | p.Asn149Lys | NP_001293108.2  [NM_001306179.1](https://www.ncbi.nlm.nih.gov/nuccore/NM_001306179.1)  **(18003757)** | NA | France | VUS |  |  |
|  | *HNF1a* | c.460A>G | p.Met154Val | NP_001293108.2  [NM_001306179.1](https://www.ncbi.nlm.nih.gov/nuccore/NM_001306179.1)  **(18003757)** | NA | France | VUS |  |  |
|  | *HNF1a* | c.461T>C | p.Met154Thr | NP_001293108.2 [NM_001306179.1](https://www.ncbi.nlm.nih.gov/nuccore/NM_001306179.1)  **(18003757)** | NA | France | VUS |  |  |
|  | *HNF1a* | c.461T>G | p.Met154Arg | NP_001293108.2 [NM_001306179.1](https://www.ncbi.nlm.nih.gov/nuccore/NM_001306179.1)  **(18003757)** | NA | France | VUS |  |  |
|  | *HNF1a* | c.517G>A | p.Val173Met | NP_001293108.2 [NM_001306179.1](https://www.ncbi.nlm.nih.gov/nuccore/NM_001306179.1)  **(18003757)** | NA | France | VUS |  |  |
|  | *HNF1a* | c.521C>T | p.Ala174Val | NP_001293108.2  [NM_001306179.1](https://www.ncbi.nlm.nih.gov/nuccore/NM_001306179.1)  **(18003757)** | 0.000195 | France | VUS |  |  |
|  | *HNF1a* | c.523C>T | p.Gln175X | NP_001293108.2  [NM_001306179.1](https://www.ncbi.nlm.nih.gov/nuccore/NM_001306179.1)  **(18003757)** |  | France | P |  |  |
|  | *HNF1a* | c.526+1G>C |  | ? |  | France |  |  |  |
|  | *HNF1a* | c.526+5G>A |  | ? |  | France |  |  |  |
|  | *HNF1a* | c.586A>G | p.Thr196Ala | NP_001293108.2  [NM_001306179.1](https://www.ncbi.nlm.nih.gov/nuccore/NM_001306179.1)  **(18003757)** | 0.000265 | France | VUS |  |  |
|  | *HNF1a* | c.614delA | p.Lys205fs | NP_001293108.2 [NM_001306179.1](https://www.ncbi.nlm.nih.gov/nuccore/NM_001306179.1) | NA | France |  |  |  |
|  | *HNF1a* | c.620_621insG | p.Gly207fs | NP_001293108.2 | NA | France |  |  |  |
|  | *HNF1a* | c.650C>G | p.Ala217Gly | NP_001293108.2 [NM_001306179.1](https://www.ncbi.nlm.nih.gov/nuccore/NM_001306179.1)  **(18003757)** | NA | France | VUS |  |  |
|  | *HNF1a* | c.676A>G | p.Lys226Glu | NP_001293108.2 [NM_001306179.1](https://www.ncbi.nlm.nih.gov/nuccore/NM_001306179.1)  **(18003757)** | NA | France | VUS |  |  |
|  | *HNF1a* | c.682_683insG | p.Glu228fs | NP_001293108.2  [NM_001306179.1](https://www.ncbi.nlm.nih.gov/nuccore/NM_001306179.1) | NA | France |  |  |  |
|  | *HNF1a* | c.682G>A | p.Glu228Lys | NP_001293108.2  [NM_001306179.1](https://www.ncbi.nlm.nih.gov/nuccore/NM_001306179.1)  **(18003757)** | NA | France | VUS |  |  |
|  | *HNF1a* | c.696_697insA | p.Val233fs | NP_001293108.2  [NM_001306179.1](https://www.ncbi.nlm.nih.gov/nuccore/NM_001306179.1) | NA | France |  |  |  |
|  | *HNF1a* | c.704_705insA | p.Cys236fs | NP_001293108.2 [NM_001306179.1](https://www.ncbi.nlm.nih.gov/nuccore/NM_001306179.1) | NA | France |  |  |  |
|  | *HNF1a* | c.711_713_6del | p.Arg238fs | NP_001293108.2 | NA | France |  |  |  |
|  | *HNF1a* | c.713+1G>C |  | ? | NA | France |  |  |  |
|  | *HNF1a* | c.715G>A | p.Ala239Thr | NP_001293108.2  [NM_001306179.1](https://www.ncbi.nlm.nih.gov/nuccore/NM_001306179.1)  **(18003757)** | NA | France | VUS |  |  |
|  | *HNF1a* | c.722G>A | p.Cys241Tyr | NP_001293108.2 [NM_001306179.1](https://www.ncbi.nlm.nih.gov/nuccore/NM_001306179.1)  **(18003757)** | NA | France | VUS |  |  |
|  | *HNF1a* | c.732A>T | p.Arg244Ser | NP_001293108.2 [NM_001306179.1](https://www.ncbi.nlm.nih.gov/nuccore/NM_001306179.1)  **(18003757)** | NA | France | VUS |  |  |
|  | *HNF1a* | c.732_733delAG | p.Ser247fs | NP_001293108.2 [NM_001306179.1](https://www.ncbi.nlm.nih.gov/nuccore/NM_001306179.1) | NA | France |  |  |  |
|  | *HNF1a* | c.737T>G | p.Val246Gly | NP_001293108.2 [NM_001306179.1](https://www.ncbi.nlm.nih.gov/nuccore/NM_001306179.1)  **(18003757)** | NA | France | VUS |  |  |
|  | *HNF1a* | c.746_747insC | p.Gln250fs | NP_001293108.2 [NM_001306179.1](https://www.ncbi.nlm.nih.gov/nuccore/NM_001306179.1) | NA | France |  |  |  |
|  | *HNF1a* | c.763G>A | p.Gly255Ser | NP_001293108.2 [NM_001306179.1](https://www.ncbi.nlm.nih.gov/nuccore/NM_001306179.1)  **(18003757)** | NA | France | VUS |  |  |
|  | *HNF1a* | c.785_786insT | p.Arg263fs | NP_001293108.2 [NM_001306179.1](https://www.ncbi.nlm.nih.gov/nuccore/NM_001306179.1) | NA | France |  |  |  |
|  | *HNF1a* | c.790G>T | p.Val264Phe | NP_001293108.2 [NM_001306179.1](https://www.ncbi.nlm.nih.gov/nuccore/NM_001306179.1)  **(18003757)** | NA | France | LP |  |  |
|  | *HNF1a* | c.798C>G | p.Asn266Lys | NP_001293108.2 [NM_001306179.1](https://www.ncbi.nlm.nih.gov/nuccore/NM_001306179.1)  **(18003757)** | NA | France | VUS |  |  |
|  | *HNF1a* | c.814C>A | p.Arg272Ser | NP_001293108.2  [NM_001306179.1](https://www.ncbi.nlm.nih.gov/nuccore/NM_001306179.1)  **(18003757)** | NA | France | LP |  |  |
|  | *HNF1a* | c.827C>G | p.Ala276Gly | NP_001293108.2  [NM_001306179.1](https://www.ncbi.nlm.nih.gov/nuccore/NM_001306179.1)  **(18003757)** | 7.14546013190519E-06 | France | LP |  |  |
|  | *HNF1a* | c.842T>C | p.Leu281Pro | NP_001293108.2 [NM_001306179.1](https://www.ncbi.nlm.nih.gov/nuccore/NM_001306179.1)  **(18003757)** |  | France | VUS |  |  |
|  | *HNF1a* | c.865C>T | p.Pro289Ser | NP_001293108.2 [NM_001306179.1](https://www.ncbi.nlm.nih.gov/nuccore/NM_001306179.1)  **(18003757)** |  | France | VUS |  |  |
|  | *HNF1a* | c.871C>A | p.Pro291Thr | NP_001293108.2  [NM_001306179.1](https://www.ncbi.nlm.nih.gov/nuccore/NM_001306179.1)  **(18003757)** | 0.00001251 | France | VUS |  |  |
|  | *HNF1a* | c.919delC | p.Leu307fs | NP_001293108.2  [NM_001306179.1](https://www.ncbi.nlm.nih.gov/nuccore/NM_001306179.1) |  | France |  |  |  |
|  | *HNF1a* | c.923C>T | p.Pro308Leu | NP_001293108.2  [NM_001306179.1](https://www.ncbi.nlm.nih.gov/nuccore/NM_001306179.1)  **18003757** | 0.0000281 | France | VUS |  |  |
|  | *HNF1a* | c.955+2T>C |  | ? |  | France |  |  |  |
|  | *HNF1a* | c.959_962dupTGCG | p.Tyr322fs | NP_001293108.2 [NM_001306179.1](https://www.ncbi.nlm.nih.gov/nuccore/NM_001306179.1) |  | France |  |  |  |
|  | *HNF1a* | c.965A>G | p.Tyr322Cys | NP_001293108.2 [NM_001306179.1](https://www.ncbi.nlm.nih.gov/nuccore/NM_001306179.1)  **18003757** | 0.000145 | France | VUS |  |  |
|  | *HNF1a* | c.966T>G | p.Tyr322X | NP_001293108.2 [NM_001306179.1](https://www.ncbi.nlm.nih.gov/nuccore/NM_001306179.1)  **18003757** | NA | France | LP |  |  |
|  | *HNF1a* | c.970C>T | p.Gln324X | NP_001293108.2  [NM_001306179.1](https://www.ncbi.nlm.nih.gov/nuccore/NM_001306179.1)  **18003757** | NA | France | P |  |  |
|  | *HNF1a* | c.984T>G | p.Ser328Arg | NP_001293108.2 [NM_001306179.1](https://www.ncbi.nlm.nih.gov/nuccore/NM_001306179.1)  **18003757** | NA | France | VUS |  |  |
|  | *HNF1a* | c.1017delT | p.Leu341X | NP_001293108.2  [NM_001306179.1](https://www.ncbi.nlm.nih.gov/nuccore/NM_001306179.1) | NA | France |  |  |  |
|  | *HNF1a* | c.1059_1060insC | p.Thr354fs | NP_001293108.2 [NM_001306179.1](https://www.ncbi.nlm.nih.gov/nuccore/NM_001306179.1) | NA | France |  |  |  |
|  | *HNF1a* | c.1080_1081dupCA | p.Ser361fs | NP_001293108.2 [NM_001306179.1](https://www.ncbi.nlm.nih.gov/nuccore/NM_001306179.1) | NA | France |  |  |  |
|  | *HNF1a* | c.1118C>G | p.Ala373Gly | NP_001293108.2  [NM_001306179.1](https://www.ncbi.nlm.nih.gov/nuccore/NM_001306179.1)  **18003757** | NA | France | VUS |  |  |
|  | *HNF1a* | c.1135C>A | p.Pro379Thr | NP_001293108.2 [NM_001306179.1](https://www.ncbi.nlm.nih.gov/nuccore/NM_001306179.1)  **18003757** | 0.0000248 | France | LP |  |  |
|  | *HNF1a* | c.1135C>G | p.Pro379Ala | NP_001293108.2 [NM_001306179.1](https://www.ncbi.nlm.nih.gov/nuccore/NM_001306179.1)  **18003757** | 0.000184769 | France | LP |  |  |
|  | *HNF1a* | c.1135C>T | p.Pro379Ser | NP_001293108.2 [NM_001306179.1](https://www.ncbi.nlm.nih.gov/nuccore/NM_001306179.1)  **18003757** | 0.00003598 | France | LP |  |  |
|  | *HNF1a* | c.1136delC | p.Pro379fs | NP_001293108.2 [NM_001306179.1](https://www.ncbi.nlm.nih.gov/nuccore/NM_001306179.1) |  | France |  |  |  |
|  | *HNF1a* | c.1137_1138insT | p.Val380fs | NP_001293108.2 [NM_001306179.1](https://www.ncbi.nlm.nih.gov/nuccore/NM_001306179.1) |  | France |  |  |  |
|  | *HNF1a* | c.1165T>G | p.Leu389Val | NP_001293108.2 [NM_001306179.1](https://www.ncbi.nlm.nih.gov/nuccore/NM_001306179.1)  **18003757** | 0.000602 | France | Likely benign |  |  |
|  | *HNF1a* | c.1195C>T | p.Gln399X | NP_001293108.2  [NM_001306179.1](https://www.ncbi.nlm.nih.gov/nuccore/NM_001306179.1)  **18003757** | NA | France | P |  |  |
|  | *HNF1a* | c.1226C>A | p.Pro409His | NP_001293108.2 [NM_001306179.1](https://www.ncbi.nlm.nih.gov/nuccore/NM_001306179.1)  **18003757** | 7.96197361401944E-06 | France | VUS |  |  |
|  | *HNF1a* | c.1271C>T | p.Pro424Leu | NP_001293108.2  [NM_001306179.1](https://www.ncbi.nlm.nih.gov/nuccore/NM_001306179.1)  **18003757** |  | France | VUS |  |  |
|  | *HNF1a* | c.1502+2A>G |  | [NM_001306179.1](https://www.ncbi.nlm.nih.gov/nuccore/NM_001306179.1)  [NP_001293108.1](https://www.ncbi.nlm.nih.gov/nuccore/NP_001293108.1) |  | France |  |  |  |
|  | *HNF1a* | c.1502+2A>T |  | [NM_001306179.1](https://www.ncbi.nlm.nih.gov/nuccore/NM_001306179.1) |  | France |  |  |  |
|  | *HNF1a* | c.1369_1383dup | p.Thr457_Pro461dup | NP_001293108.2  [NP_001293108.1](https://www.ncbi.nlm.nih.gov/nuccore/NP_001293108.1) |  | France |  |  |  |
|  | *HNF1a* | c.1387C>T | p.Gln463X | NP_001293108.2  [NM_001306179.1](https://www.ncbi.nlm.nih.gov/nuccore/NM_001306179.1)  **18003757** | NA | France | P |  |  |
|  | *HNF1a* | c.1394C>T | p.Ser465Phe | NP_001293108.2 [NM_001306179.1](https://www.ncbi.nlm.nih.gov/nuccore/NM_001306179.1)  **18003757** |  | France | VUS |  |  |
|  | *HNF1a* | c.1400C>T | p.Pro467Leu | NP_001293108.2  [NM_001306179.1](https://www.ncbi.nlm.nih.gov/nuccore/NM_001306179.1)  **18003757** | 0.00001601 | France | VUS |  |  |
|  | *HNF1a* | c.1421_1422insA | p.Pro475fs | NP_001293108.2  [NM_001306179.1](https://www.ncbi.nlm.nih.gov/nuccore/NM_001306179.1) |  | France |  |  |  |
|  | *HNF1a* | c.1444_1445delAG | p.Ser482fs | NP_001293108.2  [NM_001306179.1](https://www.ncbi.nlm.nih.gov/nuccore/NM_001306179.1) |  | France |  |  |  |
|  | *HNF1a* | c.1465T>G | p.Phe489Val | NP_001293108.2  [NM_001306179.1](https://www.ncbi.nlm.nih.gov/nuccore/NM_001306179.1)  **18003757** |  | France | VUS |  |  |
|  | *HNF1a* | c.1495C>T | p.Pro499Ser | NP_001293108.2 [NM_001306179.1](https://www.ncbi.nlm.nih.gov/nuccore/NM_001306179.1)  **18003757** |  | France | VUS |  |  |
|  | *HNF1a* | c.1498C>A | p.His500Asn | NP_001293108.2  **18003757** | 0.0000636 | France | VUS |  |  |
|  | *HNF1a* | c.1509C>A | p.Tyr503X | NP_001293108.2 [NM_001306179.1](https://www.ncbi.nlm.nih.gov/nuccore/NM_001306179.1)  **18003757** |  | France | P |  |  |
|  | *HNF1a* | c.1513C>A | p.His505Asn | NP_001293108.2 [NM_001306179.1](https://www.ncbi.nlm.nih.gov/nuccore/NM_001306179.1)  **18003757** | 0.000079696 | France | VUS |  |  |
|  | *HNF1a* | c.1522G>A | p.Glu508lys | NP_001293108.2  [NM_001306179.1](https://www.ncbi.nlm.nih.gov/nuccore/NM_001306179.1)  **18003757** | 0.0004427 | France | VUS |  |  |
|  | *HNF1a* | c.1537A>T | p.Thr513Ser | NP_001293108.2  [NM_001306179.1](https://www.ncbi.nlm.nih.gov/nuccore/NM_001306179.1)  **18003757** | 7.96463701166819E-06 | France | VUS |  |  |
|  | *HNF1a* | c.1544C>A | p.Thr515Lys | NP_001293108.2 [NM_001306179.1](https://www.ncbi.nlm.nih.gov/nuccore/NM_001306179.1)  **18003757** | 0.00003582 | France | VUS |  |  |
|  | *HNF1a* | c.1573A>T | p.Thr525Ser | NP_001293108.2  [NM_001306179.1](https://www.ncbi.nlm.nih.gov/nuccore/NM_001306179.1)  **18003757** | 0.0000389 | France | VUS |  |  |
|  | *HNF1a* | c.1574C>T | p.Thr525Ile | NP_001293108.2 [NM_001306179.1](https://www.ncbi.nlm.nih.gov/nuccore/NM_001306179.1)  **18003757** | NA | France | VUS |  |  |
|  | *HNF1a* | c.1576G>A | p.Asp526Asn | NP_001293108.2  [NM_001306179.1](https://www.ncbi.nlm.nih.gov/nuccore/NM_001306179.1)  **18003757** | 0.000019 | France | LP |  |  |
|  | *HNF1a* | c.1576G>T | p.Asp526Tyr | NP_001293108.2  [NM_001306179.1](https://www.ncbi.nlm.nih.gov/nuccore/NM_001306179.1)  **18003757** | NA | France | LP |  |  |
|  | *HNF1a* | c.1587_1588insA | p.Asn529fs | NP_001293108.2  [NM_001306179.1](https://www.ncbi.nlm.nih.gov/nuccore/NM_001306179.1) | NA | France |  |  |  |
|  | *HNF1a* | c.1611_1614delGCCC | p.Pro538fs | NP_001293108.2  [NM_001306179.1](https://www.ncbi.nlm.nih.gov/nuccore/NM_001306179.1) | NA | France |  |  |  |
|  | *HNF1a* | c.1623+2T>C |  | ? |  | France |  |  |  |
|  | *HNF1a* | c.1637A>G | p.Asp546Gly | ?  **18003757** |  | France | VUS |  |  |
|  | *HNF1a* | c.1663C>T | p.Leu555Phe | ? | 8.05892687329755E-06 | France | VUS |  |  |
|  | *HNF1a* | c.1670_1685dup | p.His563GlnfsTer2 | [NM_001306179.1](https://www.ncbi.nlm.nih.gov/nuccore/NM_001306179.1) [NP_001293108.1](https://www.ncbi.nlm.nih.gov/nuccore/NP_001293108.1) | NA | France |  |  |  |
|  | *HNF1a* | c.1673_1674insC | p.Ala559fs | [NM_001306179.1](https://www.ncbi.nlm.nih.gov/nuccore/NM_001306179.1) [NP_001293108.1](https://www.ncbi.nlm.nih.gov/nuccore/NP_001293108.1) | NA | France |  |  |  |
|  | *HNF1a* | c.1762C>T | p.Pro588Ser | ?  **18003757** | 4.18154600118755E-06 | France | VUS |  |  |
|  | *HNF1a* | c.1840_1841delAA | p.Asn614fs | ? |  | France |  |  |  |
|  | *HNF1a* | c.1853_1854delTC | p.Ile618fs | ? |  | France |  |  |  |
|  | *HNF1a* | c.1864_1890dup | p.Ile622_Ser630dup | [NM_001306179.1](https://www.ncbi.nlm.nih.gov/nuccore/NM_001306179.1) [NP_001293108.1](https://www.ncbi.nlm.nih.gov/nuccore/NP_001293108.1) |  | France |  |  |  |
|  | *HNF1a* |  | p.Ala15Ala | NP_001293108.2 |  | Brazil |  | 2012 | ([128](#_ENREF_128)) |
|  | *HNF1a* |  | p.Gln141Gln | NP_001293108.2 |  | Brazil |  |  |  |
|  | *HNF1a* | c.599G>A | p.Arg200Gln | NP_001293108.2  **9472859** | 3.97873762612598E-06 | USA | LP | 2016 | ([67](#_ENREF_67)) |
|  | *HNF1a* | c.1136C>A | p.Pro379His | NP_001293108.2 [NM_001306179.1](https://www.ncbi.nlm.nih.gov/nuccore/NM_001306179.1)  **15883474** | 0.00005197 | USA | VUS |  |  |
|  | *HNF1a* |  | p.Glu235Gly | NP_001293108.2  **21170474** |  | Spain |  | 2011 | ([129](#_ENREF_129)) |
|  | *HNF1a* | c-57-64delCACGCGGT |  | ? |  | Spain |  |  |  |
|  | *HNF1a* | c.117_119delAAG | p.Lys39del | ? |  | Russia |  | 2019 | ([130](#_ENREF_130)) |
|  | *HNF1a* | c.1346_1347delCG | p.Ala449fs | ? |  | Russia |  |  |  |
|  | *HNF1a* | c.868G>C | p.Glu290Gln | ? |  | Russia | VUS |  |  |
|  | *HNF1a* | c.1253G>C | p.Ser418Thr | ? |  | Russia |  |  |  |
|  | *HNF1a* | c.199G>T | p.Glu67* | ? |  | Russia |  |  |  |
|  | *HNF1a* | c.485T>G | p.Leu162Arg | NP_001293108.2  [NM_001306179.1](https://www.ncbi.nlm.nih.gov/nuccore/NM_001306179.1) |  | Russia | LP |  |  |
|  | *HNF1a* | c.526+1G>A |  | [NM_001306179.1](https://www.ncbi.nlm.nih.gov/nuccore/NM_001306179.1)  [NP_001293108.1](https://www.ncbi.nlm.nih.gov/nuccore/NP_001293108.1) |  | Saudi Arabia |  | 2011 | ([131](#_ENREF_131)) |
|  | *HNF1a* | c.1761C > G | p.Pro588Ala | ? |  | Germany | Likely benign | 2016 | ([132](#_ENREF_132)) |
|  | *HNF1a* | c.1765_1766delinsGCCCGfs86* | p.His589AlafsTer67 | [NM_001306179.1](https://www.ncbi.nlm.nih.gov/nuccore/NM_001306179.1) [NP_001293108.1](https://www.ncbi.nlm.nih.gov/nuccore/NP_001293108.1) |  | Germany |  |  |  |
|  | *HNf1a* |  | p.Arg363Cys | ? |  | Italy |  | 2009 | ([72](#_ENREF_72)) |
|  | *HNF1a* |  | p.Cys49Gly | ? |  | Iran |  | 2018 | ([133](#_ENREF_133)) |
|  | *HNF1a* |  | p.Trp113X | NP_001293108.2  **11692182** |  | Brazil |  | 2011 | ([134](#_ENREF_134)) |
|  | *HNF1a* |  | p.Gly292fs | NP_001293108.2 |  | Malta |  | 2019 | ([135](#_ENREF_135)) |
|  | *HNF1a* |  | p.Ser3Cys | NP_001293108.2 |  | Croatia |  | 2018 | ([136](#_ENREF_136)) |
|  | *HNF1a* |  | p.Gly151Ser | NP_001293108.2 |  | Croatia |  |  |  |
|  | *HNF1a* | c.155G>C | p.Gly52Ala | NP_001293108.2 [NM_001306179.1](https://www.ncbi.nlm.nih.gov/nuccore/NM_001306179.1)  **23771925** | 0.00027 | USA | VUS | 2013 | ([77](#_ENREF_77)) |
|  | *HNF1a* | c.725T>C | p.Ile242Thr | NP_001293108.2 [NM_001306179.1](https://www.ncbi.nlm.nih.gov/nuccore/NM_001306179.1)  **23771925** | NA | USA | VUS |  |  |
|  | *HNF1a* | c.800G>C | p.trp267Ser | NP_001293108.2 [NM_001306179.1](https://www.ncbi.nlm.nih.gov/nuccore/NM_001306179.1)  **23771925** | NA | USA | VUS |  |  |
|  | *HNF1a* | c.872C>A | p.Pro291Q | NP_001293108.2  [NM_001306179.1](https://www.ncbi.nlm.nih.gov/nuccore/NM_001306179.1)  **23771925** | 0.0000209191 | USA | LP |  |  |
|  | *HNF1a* | c.1124G>T | p.Gly375Val | NP_001293108.2  [NM_001306179.1](https://www.ncbi.nlm.nih.gov/nuccore/NM_001306179.1)  **23771925** | 4.00641025641025E-06 | USA | VUS |  |  |
|  | *HNF1a* | c.1504C>G | p.Lys502Val | NP_001293108.2 [NM_001306179.1](https://www.ncbi.nlm.nih.gov/nuccore/NM_001306179.1)  **23771925** | NA | USA | VUS |  |  |
|  | *HNF1a* | c.676delAAG | p.lys226del | NP_001293108.2 | NA | USA |  |  |  |
|  | *HNF1a* | c.607C>T | p.Arg203Cys | NP_001293108.2  **10078571** | 0.0000039789 | Thailand |  | 2019 | ([137](#_ENREF_137)) |
|  | *HNF1a* | IVS1+1G>A |  | ? | NA | USA |  | 2015 | ([78](#_ENREF_78)) |
|  | *HNF1a* | c.734G>T | p.Gly245Val | NP_001293108.2 NM_001306179.1  (19336507) |  | UK | LP | 2018 | ([138](#_ENREF_138)) |
|  | *HNF1a* | c.398T>A | p.Val133Glu | NP_001293108.2  [NM_001306179.1](https://www.ncbi.nlm.nih.gov/nuccore/NM_001306179.1)  (18003757) |  | Brazil | VUS | 2019 | ([79](#_ENREF_79)) |
|  | *HNF1a* | c.1296_1297insC | p.Thr433Hisfs*116 | NP_001293108.2 [NM_001306179.1](https://www.ncbi.nlm.nih.gov/nuccore/NM_001306179.1) |  | Brazil |  |  |  |
|  | *HNF1a* | P291fsinsC c.872dup | p.Gly292ArgfsTer25 | NP_001293108.2  [NM_001306179.1](https://www.ncbi.nlm.nih.gov/nuccore/NM_001306179.1) |  | USA |  | 2013 | ([139](#_ENREF_139)) |
|  | *HNF1a* | c.1349dup | p.Asn450LysfsTer106 | [NM_001306179.1](https://www.ncbi.nlm.nih.gov/nuccore/NM_001306179.1) [NP_001293108.1](https://www.ncbi.nlm.nih.gov/nuccore/NP_001293108.1) |  | UK |  | 2017 | ([140](#_ENREF_140)) |
|  | *HNF1a* | c.391C>T | p.Arg131Trp | [NM_001306179.1](https://www.ncbi.nlm.nih.gov/nuccore/NM_001306179.1)  [NP_001293108.1](https://www.ncbi.nlm.nih.gov/nuccore/NP_001293108.1)  **9166684** |  | UK | VUS |  |  |
|  | *HNF1a* | c.495G>C | p.Trp165Cys | [NM_001306179.1](https://www.ncbi.nlm.nih.gov/nuccore/NM_001306179.1) [NP_001293108.1](https://www.ncbi.nlm.nih.gov/nuccore/NP_001293108.1) |  | UK | VUS |  |  |
|  | *HNF1a* | c.28A>C | p.Thr10Pro | [NM_001306179.1](https://www.ncbi.nlm.nih.gov/nuccore/NM_001306179.1)  [NP_001293108.1](https://www.ncbi.nlm.nih.gov/nuccore/NP_001293108.1)  (23348805) |  | UK | VUS |  |  |
|  | *HNF1a* | c.814C>T, | p.Arg272Cys | [NM_001306179.1](https://www.ncbi.nlm.nih.gov/nuccore/NM_001306179.1)  [NP_001293108.1](https://www.ncbi.nlm.nih.gov/nuccore/NP_001293108.1)  **23348805** |  | UK | LP |  |  |
|  | *HNF1a* | c.-258A>G |  | ? |  | UK |  |  |  |
|  | *HNF1a* | c.779 C>T | p.Thr260Met | NP_001293108.2 [NM_001306179.1](https://www.ncbi.nlm.nih.gov/nuccore/NM_001306179.1)  **9166684** | 4.01174639343999E-06 | China | LP | 2017 | ([141](#_ENREF_141)) |
|  | *HNF1a* |  | p.Val264fs | NP_001293108.2 |  | Spain |  | 2019 | ([142](#_ENREF_142)) |
|  | *HNF1a* | c.864delGinsCC | p.Gly292ArgfsX25 | NP_001293108.2  [NM_001306179.1](https://www.ncbi.nlm.nih.gov/nuccore/NM_001306179.1) |  | NewZealand |  | 2013 | ([88](#_ENREF_88)) |
|  | *HNF1a* |  | p.Gln243Glu | NP_001293108.2  (15657605) |  | China |  | 2005 | ([143](#_ENREF_143)) |
|  | *HNF1a* | c.932C>A | p.Ala311Asp | NP_001293108.2 | 9.48964679534627E-06 | China |  |  |  |
|  | *HNF1a* | c.1136C>G | p.Pro379Arg | NP_001293108.2 | 3.99817683136489E-06 | China |  |  |  |
|  | *HNF1a* |  | p.Pro488fsdelC | NP_001293108.2 | NA | China |  |  |  |
|  | *HNF1a* | c.758G>A | p.Gly253Glu | NP_001293108.2  [NM_001306179.1](https://www.ncbi.nlm.nih.gov/nuccore/NM_001306179.1)  (23348805) | NA | Iran | VUS | 2019 | ([144](#_ENREF_144)) |
|  | *HNF4a* | c.G25A | p.Asp9Asn | NP_000448.3 | 7.07108562377581E-06 | Canada | VUS | 2016 | ([7](#_ENREF_7)) |
|  | *HNF4a* | Phe75fsdelT |  | ? | NA | Denmark |  | 1999 | ([145](#_ENREF_145)) |
|  | *HNF4a* | c.-1009G>C |  | ? | NA | India | LP | 2011 | ([146](#_ENREF_146)) |
|  | *HNF4a* | c.-129T>C |  | ? | NA | India | Likely benign |  |  |
|  | *HNF4a* | c.-79C>T |  | ? | NA | India | VUS |  |  |
|  | *HNF4a* | c.640C>T | p.His214Tyr | ? | NA | Netherland | LP | 2009 | ([92](#_ENREF_92)) |
|  | *HNF4a* |  | p.Glu276Gln | ? | NA | UK |  | 1997 | ([147](#_ENREF_147)) |
|  | *HNF4a* | c.811G>A | p.Asp271Asn | [NM_001287182.1](https://www.ncbi.nlm.nih.gov/nuccore/NM_001287182.1)  [NP_001274111.1](https://www.ncbi.nlm.nih.gov/nuccore/NP_001274111.1) | NA | India | LP | 2015 | ([93](#_ENREF_93)) |
|  | *HNF4a* | c.58A>C |  |  | NA | Italy | VUS | 1997 | ([148](#_ENREF_148)) |
|  | *HNF4a* |  | p.Ala193Val | NP_001274113.1 | NA | Israel |  | 2007 | ([15](#_ENREF_15)) |
|  | *HNF4A* |  | p.Glu285Lys | NP_000448.3 | NA | Greece |  | 2020 | ([16](#_ENREF_16)) |
|  | *HNF4a* | c.572delT | p.Leu191fs | NP_001245284.1 | NA | Ukraine |  | 2017 | ([99](#_ENREF_99)) |
|  | *HNF4a* |  | p.Arg127trp | NP_001245284.1 | NA | Japan |  | 1997 | ([149](#_ENREF_149)) |
|  | *HNF4a* | c.146A>G | p.His49Arg | NP_001025175.1 | NA | USA | LP | 2015 | ([25](#_ENREF_25)) |
|  | *HNF4a* | c.353G>A | p.Arg118Gln | NP_001025175.1  [NM_001287182.1](https://www.ncbi.nlm.nih.gov/nuccore/NM_001287182.1)  (23348805) | NA | USA | VUS |  |  |
|  | *HNF4a* | c.318_331del | p.Val108Gfs*11 | NP_001025175.1 | NA | USA |  |  |  |
|  | *HNF4a* | c.878_881dup | p.Gln294Hfs*15 | NP_001025175.1 | NA | USA |  |  |  |
|  | *HNF4a* | c.556>557 GATinsGfs | p.Asp177fsinsG | ? | NA | Denmark |  | 2005 | ([28](#_ENREF_28)) |
|  | *HNF4a* | c.929GA CGG3CAG | p.Arg301Gln | NP_001274112.1 | NA | Denmark | LP |  |  |
|  | *HNF4a* | c.734G>C | p.Arg245Pro | NP_001025175.1 | NA | UK | LP | 2019 | ([150](#_ENREF_150)) |
|  | *HNF4a* | c.426+1G>A |  | ? | NA | Italy |  | 2017 | ([40](#_ENREF_40)) |
|  | *HNF4a* | c.37A>T | p.Lys13* | ? |  | USA |  | 2019 | ([151](#_ENREF_151)) |
|  | *HNF4a* | c.295del | p.Trp99Glyfs*3 | ? |  | USA |  |  |  |
|  | *HNF4a* | c.317_333del | p.Gln106Argfs*9 | NP_001274112.1 | NA | USA |  |  |  |
|  | *HNF4a* | c.344dup | p.Met115Ilefs*6 | [NM_001287182.1](https://www.ncbi.nlm.nih.gov/nuccore/NM_001287182.1)  [NP_001274111.1](https://www.ncbi.nlm.nih.gov/nuccore/NP_001274111.1) |  | USA |  |  |  |
|  | *HNF4a* | c.848del Asn283Thrfs*11 | p.Ile283ThrfsTer10 | [NM_001287182.1](https://www.ncbi.nlm.nih.gov/nuccore/NM_001287182.1)  [NP_001274111.1](https://www.ncbi.nlm.nih.gov/nuccore/NP_001274111.1) |  | USA |  |  |  |
|  | *HNF4a* | c.1082dupC | p.T362Hisfs*97 | ? |  | USA |  |  |  |
|  | *HNF4a* | c.1113C>A | p.Cys371* | ? |  | USA | LP |  |  |
|  | *HNF4a* | c.2T>C | p.Met1 | NP_000448.3  [NM_001287182.1](https://www.ncbi.nlm.nih.gov/nuccore/NM_001287182.1)  (21683639) | 3.97778803163137E-06 | Ireland |  | 2011 | ([111](#_ENREF_111)) |
|  | *HNF4a* | c.868C>T | p.Arg290Cys | NP_001025175.1  (21683639) | NA | Ireland | LP |  |  |
|  | *HNF4a* | c.842-2delA |  | ? | NA | Spain |  | 2002 | ([47](#_ENREF_47)) |
|  | *HNF4a* |  | p.Met1Ile | NP_000448.3  (21683639) | NA | Korea |  | 2019 | ([53](#_ENREF_53)) |
|  | *HNF4a* | c.956T>C, | p.Leu319Pro | NP_000448.3  [NM_001287182.1](https://www.ncbi.nlm.nih.gov/nuccore/NM_001287182.1)  (15830177) | NA | Korea | LP | 2016 | ([115](#_ENREF_115)) |
|  | *HNF4a* | c.344G>A, | p.Cys115tyr | NP_000448.3  [NM_001287182.1](https://www.ncbi.nlm.nih.gov/nuccore/NM_001287182.1) | NA | Turkey | LP | 2018 | ([55](#_ENREF_55)) |
|  | *HNF4a* | c.149C>T, | p.Ala50Val | NP_000448.3  [NM_001287182.1](https://www.ncbi.nlm.nih.gov/nuccore/NM_001287182.1) | 0.0000212 | Turkey | VUS |  |  |
|  | *HNF4a* | c.736+2T>A |  | ? | NA | Turkey |  |  |  |
|  | *HNF4a* | c.736G>A | p.Gly246Ser | NP_000448.3 [NM_001287182.1](https://www.ncbi.nlm.nih.gov/nuccore/NM_001287182.1) | NA | Turkey | LP |  |  |
|  | *HNF4a* | c.534_536delGAA | p.lys179del | NP_849181.1 | NA | Turkey |  |  |  |
|  | *HNF4a* |  | p.Gln142His | NP_001025175.1 | NA | Japan |  | 2018 | ([152](#_ENREF_152)) |
|  | *HNF4a* |  | p.Glu256Ala | NP_001025175.1 | NA | Japan |  |  |  |
|  | *HNF4a* |  | p.Arg331His | NP_000448.3 | 4.02197607728629E-06 | China |  | 2019 | ([123](#_ENREF_123)) |
|  | *HNF4A* | c.317G>A | p.Met116Ile | ? | NA | UK | LP | 2014 | ([153](#_ENREF_153)) |
|  | *HNF4a* | c.575_582+10del |  | ? | NA | USA |  | 2016 | ([67](#_ENREF_67)) |
|  | *HNF4a* | c.932G>A | p.Arg311His | NP_001025175.1  (10768098) | NA | USA | LP |  |  |
|  | *HNF4a* | c.7A>G | p.Ser3Gly | NP_001025175.1 | 4.04226593259117E-06 | Brazil | VUS | 2019 | ([68](#_ENREF_68)) |
|  | *HNF4a* | c.145C>T | p.His49Tyr | NP_001025175.1 | NA | Brazil | LP |  |  |
|  | *HNF4a* |  | p.Arg80Gln |  | NA | Italy |  | 2010 | ([154](#_ENREF_154)) |
|  | *HNF4a* |  | p.Val393Ile |  | NA | France |  | 1998 | ([155](#_ENREF_155)) |
|  | *HNF4a* |  | p. Arg244Gln |  | NA | Japan |  | 2002 | ([156](#_ENREF_156)) |
|  | *HNF4a* | c.266G>A | p.Arg89Gln | NP_000448.3 | NA | Norway | LP | 2012 | ([157](#_ENREF_157)) |
|  | *HNF4a* | c.200G>A | p.Arg67Gln | NP_001025175.1  (20705777) | NA | USA | LP | 2019 | ([158](#_ENREF_158)) |
|  | *HNF4a* |  | p.Arg114Gln | NP_001245284.1 | NA | UK |  | 2016 | ([159](#_ENREF_159)) |
|  | *HNF4a* |  | p.Arg154 | ? | NA | germany |  | 1997 | ([160](#_ENREF_160)) |
|  | *HNF4a* | c.932G>A | p.Arg311His | NP_001025175.1  (10768098) | NA | Italy | LP | 2014 | ([161](#_ENREF_161)) |
|  | *HNF4a* | c.503G>A | p.Arg168Gln | ?  (23771925) | NA | USA | LP | 2013 | ([77](#_ENREF_77)) |
|  | *HNF4a* | c.779T>C | p.Lys260Pro | ?  (23771925) | NA | USA | LP |  |  |
|  | *HNF4a* | c.1183G>A | p.Val395Ile | NP_001245284.1  23771925 | NA | USA | VUS |  |  |
|  | *HNF4a* |  | p.Gly326arg |  | NA | Norway |  | 2006 | ([162](#_ENREF_162)) |
|  | *HNF4a* |  | p.Thr339Ile |  | NA | Norway |  |  |  |
|  | *HNF4a* |  | p.Trp340X |  | NA | Norway |  |  |  |
|  | *HNF4a* |  | p.Arg127Trp | NP_001245284.1 | NA | USA |  | 2013 | ([139](#_ENREF_139)) |
|  | *HNF4a* | c.340C>T | p.Arg114Trp | NP_001025175.1  (9313765) | NA | UK | VUS | 2016 | ([83](#_ENREF_83)) |
|  | *HNF4a* | [c.12G>A](https://variantvalidator.org/service/validate/) | p.Trp4Ter | [NM_001287182.1](https://www.ncbi.nlm.nih.gov/nuccore/NM_001287182.1)  [NP_001274111.1](https://www.ncbi.nlm.nih.gov/nuccore/NP_001274111.1) | NA | UK | P |  |  |
|  | *HNF4a* | c.1064-5_1070del |  |  | NA | UK |  | 2017 | ([140](#_ENREF_140)) |
|  | *HNF4a* | c.-12G>A | p.Trp4Ter | [NM_001287182.1](https://www.ncbi.nlm.nih.gov/nuccore/NM_001287182.1)  [NP_001274111.1](https://www.ncbi.nlm.nih.gov/nuccore/NP_001274111.1) | NA | UK | P |  |  |
|  | *HNF4a* |  | p.Val255Met |  | NA | Iran |  | 2009 | ([163](#_ENREF_163)) |
|  | *HNF1B* | c.274C>T | p.Leu92Phe | [NM_000458.3](https://www.ncbi.nlm.nih.gov/nuccore/NM_000458.3)  [NP_000449.1](https://www.ncbi.nlm.nih.gov/nuccore/NP_000449.1)  (25041077) | NA | India | VUS | 2015 | ([93](#_ENREF_93)) |
|  | *HNF1b* |  | p.Arg112Pro | NP_000449.1  (15068978) | NA | France |  | 2004 | ([164](#_ENREF_164)) |
|  | *HNF1b* |  | p.Gln136Glu | NP_000449.1  (15068978) | NA | France |  |  |  |
|  | *HNF1b* |  | p.lys164Gln | NP_000449.1  (15068978) | NA | France |  |  |  |
|  | *HNF1b* |  | p.Arg165His | NP_000449.1  (15068978) | NA | France |  |  |  |
|  | *HNF1b* |  | p.Arg181X | NP_000449.1  (15068978) | NA | France |  |  |  |
|  | *HNF1b* |  | p.Arg182X | NA | NA | France |  |  |  |
|  | *HNF1b* | IVS21G3T |  |  | NA | France |  |  |  |
|  | *HNF1b* |  | p.Arg295His | NP_000449.1  (15068978) | NA | France |  |  |  |
|  | *HNF1b* | 443 C>T | p.Cys443Thr | NP_000449.1 | NA | Germany |  | 2008 | ([165](#_ENREF_165)) |
|  | *HNF1b* |  | p.Ser148Leu | NP_000449.1  (15930087) | NA | Japan |  | 2014 | ([101](#_ENREF_101)) |
|  | *HNF1b* | 354Q>X | p.Gln354X | NP_000449.1  (24905847) | NA | Japan |  |  |  |
|  | *HNF1b* | c.393A>T | p.Gln131His | NP_000449.1 [NM_000458.3](https://www.ncbi.nlm.nih.gov/nuccore/NM_000458.3) [NP_000449.1](https://www.ncbi.nlm.nih.gov/nuccore/NP_000449.1) | NA | Norway | VUS | 2017 | ([104](#_ENREF_104)) |
|  | *HNF1b* | c.867C>G | p.Asn289Lys | NP_000449.1 [NM_000458.3](https://www.ncbi.nlm.nih.gov/nuccore/NM_000458.3) | NA | Norway | VUS |  |  |
|  | *HNF1b* | c.883C>T | p.Arg295Cys | NP_000449.1  [NM_000458.3](https://www.ncbi.nlm.nih.gov/nuccore/NM_000458.3)  (16249435) | NA | Norway | VUS |  |  |
|  | *HNF1b* | c.1085C>T | p.Ser362Phe | [NM_000458.3](https://www.ncbi.nlm.nih.gov/nuccore/NM_000458.3)  NP_000449.1 | NA | Norway | VUS |  |  |
|  | *HNF1b* | c.124G>A | p.Gly42Arg | NA | NA | Norway |  |  |  |
|  | *HNF1b* |  | p.A263fsinsGG | NP_000449.1 | NA | Japan |  | 2016 | ([166](#_ENREF_166)) |
|  | *HNF1b* | c.727C>T | p.Gln243X | NP_000449.1 [NM_000458.3](https://www.ncbi.nlm.nih.gov/nuccore/NM_000458.3) | NA | USA | P | 2013 | ([167](#_ENREF_167)) |
|  | *HNF1b* |  | p.Leu168Pro | NP_000449.1 | NA | Japan |  | 2018 | ([152](#_ENREF_152)) |
|  | *HNF1b* | 160M>V | p.Met160Val | NP_000449.1  (22034641) | NA | Australia |  | 2014 | ([168](#_ENREF_168)) |
|  | *HNF1b* | c.1007A > G | p.His336Arg | NP_000449.1  [NM_000458.3](https://www.ncbi.nlm.nih.gov/nuccore/NM_000458.3)  (16971658) | NA | China | VUS | 2017 | ([169](#_ENREF_169)) |
|  | *HNF1b* | 239G>E | p.Gly239Glu | NP_000449.1 | NA | China |  | 2017 | ([170](#_ENREF_170)) |
|  | *HNF1b* | c.1005dupC | p.His336fs | NP_000449.1  [NM_000458.3](https://www.ncbi.nlm.nih.gov/nuccore/NM_000458.3)  (16971658) | NA | germany |  | 2017 | ([62](#_ENREF_62)) |
|  | *HNF1b* | c.143delT | p.Leu48fs | NP_000449.1 [NM_000458.3](https://www.ncbi.nlm.nih.gov/nuccore/NM_000458.3) | NA | France |  | 2005 | ([171](#_ENREF_171)) |
|  | *HNF1b* | c.226G>T | p.Gly76Cys | NP_000449.1  [NM_000458.3](https://www.ncbi.nlm.nih.gov/nuccore/NM_000458.3)  (16249435) | 0.000549984 | France | LP |  |  |
|  | *HNF1b* | c.406C>T | p.Gln136X | NP_000449.1 [NM_000458.3](https://www.ncbi.nlm.nih.gov/nuccore/NM_000458.3)  (16249435) | NA | France | P |  |  |
|  | *HNF1b* | c.704G>A | p.Arg235Gln | NP_000449.1  [NM_000458.3](https://www.ncbi.nlm.nih.gov/nuccore/NM_000458.3)  (16249435) | NA | France | VUS |  |  |
|  | *HNF1b* | c.826G>A | p.Arg276Gly | NP_000449.1  (16249435) | NA | France | P |  |  |
|  | *HNF1b* | c.854G>A | p.Gly285Asp | NP_000449.1  [NM_000458.3](https://www.ncbi.nlm.nih.gov/nuccore/NM_000458.3)  (16249435) | NA | France | VUS |  |  |
|  | *HNF1b* | c.883CT | p.Arg295Cys | NP_000449.1  [NM_000458.3](https://www.ncbi.nlm.nih.gov/nuccore/NM_000458.3)  (16249435) | NA | France | VUS |  |  |
|  | *HNF1b* | c.1108G>A | p.Gly370Ser | NP_000449.1  (16249435) | 0.000180 | France | LP |  |  |
|  | *HNF1b* | c.1046–294_1206 704del | p.Gly349_Met402del | NP_000449.1  XP_011523462.1 | NA | France |  |  |  |
|  | *HNF1b* | c.1+1671del | p.Met1_Trp557del | NP_000449.1 | NA | France |  |  |  |
|  | *HNF1b* | c207_211delCGCCA | p.His69GlnfsTer17 | [NM_000458.3](https://www.ncbi.nlm.nih.gov/nuccore/NM_000458.3)  [NP_000449.1](https://www.ncbi.nlm.nih.gov/nuccore/NP_000449.1) | NA | Spain |  | 2017 | ([172](#_ENREF_172)) |
|  | *HNF1b* |  | p.Arg276X | NP_000449.1  (12161522) | NA | Japan |  | 2007 | ([173](#_ENREF_173)) |
|  | *HNF1b* | c.578T>C | p.Met193Thr | [NM_000458.3](https://www.ncbi.nlm.nih.gov/nuccore/NM_000458.3)  [NP_000449.1](https://www.ncbi.nlm.nih.gov/nuccore/NP_000449.1) | 3.97845270017584E-06 | Japan | VUS | 2012 | ([174](#_ENREF_174)) |
|  | *HNf1b* | c.434T>A | p.Leu145Gln | NP_000449.1  [NM_000458.3](https://www.ncbi.nlm.nih.gov/nuccore/NM_000458.3) | NA | japan | VUS | 2018 | ([175](#_ENREF_175)) |
|  | *HNF1b* |  | p.Pro159Leu | NP_000449.1  (20378641) | NA | Korea |  | 2014 | ([176](#_ENREF_176)) |
|  | *HNF1b* | c.1580G>A | p.Arg527Gln | NP_000449.1  [NM_000458.3](https://www.ncbi.nlm.nih.gov/nuccore/NM_000458.3) | 0.00001590 | Malta | VUS | 2018 | ([177](#_ENREF_177)) |
|  | *HNF1b* | c.1-*151+del |  |  | NA | UK |  | 2016 | ([83](#_ENREF_83)) |
|  | *HNF1b* | c.1-_1674+del |  |  | NA | UK |  | 2017 | ([140](#_ENREF_140)) |
|  | *HNF1b* | c.77C>T |  |  | NA | China |  | 2016 | ([178](#_ENREF_178)) |
|  | *INS* | c.89T>G | p.Leu30Arg | NP_000198.1  [NM_001291897.1](https://www.ncbi.nlm.nih.gov/nuccore/NM_001291897.1) |  | Canada | LP | 2016 | ([7](#_ENREF_7)) |
|  | *INS* | c.125T>C | pVal42Ala | NP_000198.1  [NM_001291897.1](https://www.ncbi.nlm.nih.gov/nuccore/NM_001291897.1) |  | Italy | LP | 2016 | ([179](#_ENREF_179)) |
|  | *INS* | c.163C>T | p.Arg55Cys | NP_000198.1 [NM_001291897.1](https://www.ncbi.nlm.nih.gov/nuccore/NM_001291897.1)  (18192540) |  | Norway | LP | 2013 | ([22](#_ENREF_22)) |
|  | *INS* | c.–331delC |  | ? |  | USA |  | 2015 | ([25](#_ENREF_25)) |
|  | *INS* | c.265CNT | p.Arg89Cys | [NM_001291897.1](https://www.ncbi.nlm.nih.gov/nuccore/NM_001291897.1)  [NP_001278826.1](https://www.ncbi.nlm.nih.gov/nuccore/NP_001278826.1)  (17855560) |  | USA | LP |  |  |
|  | *INS* | c.4G>A | p.Ala2Thr | NP_000198.1 | 0.0000324408495 | china |  | 2017 | ([180](#_ENREF_180)) |
|  | *INS* | c.277G>A | p.Glu93Lys | NP_000198.1  [NM_001291897.1](https://www.ncbi.nlm.nih.gov/nuccore/NM_001291897.1) | NA | Australia |  | 2018 | ([181](#_ENREF_181)) |
|  | *INS* | c.233delA | p.Gln78fs | NP_000198.1 [NM_001291897.1](https://www.ncbi.nlm.nih.gov/nuccore/NM_001291897.1) | NA | Czech Republic |  | 2015 | ([182](#_ENREF_182)) |
|  | *INS* |  | p.Gly32Ser | NP_000198.1  (17855560) | NA | Japan |  | 2016 | ([110](#_ENREF_110)) |
|  | *INS* |  | p.Gly75Cys | NP_000198.1 | NA | Japan |  | 2018 | ([152](#_ENREF_152)) |
|  | *INS* |  | p.Cys96Phe | NP_000198.1 | NA | Japan |  |  |  |
|  | *INS* | c.212dupG | p.Gly73fs | NP_000198.1 [NM_001291897.1](https://www.ncbi.nlm.nih.gov/nuccore/NM_001291897.1) | NA | China |  | 2019 | ([183](#_ENREF_183)) |
|  | *INS* | c.298T>G | p.Cys100Gly | NP_000198.1 [NM_001291897.1](https://www.ncbi.nlm.nih.gov/nuccore/NM_001291897.1) | NA | China | LP | 2019 | ([61](#_ENREF_61)) |
|  | *INS* | c.17G>A | p.Arg6His | NP_000198.1 [NM_001291897.1](https://www.ncbi.nlm.nih.gov/nuccore/NM_001291897.1)  (20007936) | 0.0000863850 | Denmark | VUS | 2010 | ([184](#_ENREF_184)) |
|  | *INS* | c.65delC | p.Ala23GlnfsTer4 | NP_000198.1 [NM_001291897.1](https://www.ncbi.nlm.nih.gov/nuccore/NM_001291897.1) | NA | Brazil |  | 2019 | ([68](#_ENREF_68)) |
|  | *INS* | c.163C>T | p.Arg55Cys | NP_000198.1  [NM_001291897.1](https://www.ncbi.nlm.nih.gov/nuccore/NM_001291897.1)  (18192540) | NA | Norway | LP | 2008 | ([185](#_ENREF_185)) |
|  | *ABCC8* | c.3202T>A | p.Phe1068Ile | NP_001274103.1 |  | UK | VUS | 2019 | ([186](#_ENREF_186)) |
|  | *ABCC8* | c.2376delC | p.Phe793Serfs*71 | NP_000343.2  [NM_000352.4](https://www.ncbi.nlm.nih.gov/nuccore/NM_000352.4) |  | Tunisia |  | 2019 | ([187](#_ENREF_187)) |
|  | *ABCC8* | c.4608+4A>G |  | [NM_000352.4](https://www.ncbi.nlm.nih.gov/nuccore/NM_000352.4) [NP_000343.2](https://www.ncbi.nlm.nih.gov/nuccore/NP_000343.2) |  | Tunisia |  |  |  |
|  | *ABCC8* |  | p.Met1514Thr | NP_001274103.1 |  | Greece |  | 2020 | ([16](#_ENREF_16)) |
|  | *ABCC8* |  | p.Ser1386Phe | NP_001274103.1  (10204114) |  | Greece |  |  |  |
|  | *ABCC8* | [c.1277A>G](https://variantvalidator.org/service/validate/) | p.Asn426Ser | NP_001274103.1  [NM_000352.4](https://www.ncbi.nlm.nih.gov/nuccore/NM_000352.4) | 3.97649098529493E-06 | USA | VUS | 2015 | ([25](#_ENREF_25)) |
|  | *ABCC8* | [c.3545G>A](https://variantvalidator.org/service/validate/) | p.Arg1182Gln | [NM_000352.4](https://www.ncbi.nlm.nih.gov/nuccore/NM_000352.4)  [NP_000343.2](https://www.ncbi.nlm.nih.gov/nuccore/NP_000343.2)  (16885549) |  |  | LP |  |  |
|  | *ABCC8* | [c.1608T>G](https://variantvalidator.org/service/validate/) | p.Phe536Leu | NP_001274103.1 [NM_000352.4](https://www.ncbi.nlm.nih.gov/nuccore/NM_000352.4)  (25555642) |  | USA | VUS |  |  |
|  | *ABCC8* | [c.2422C>A](https://variantvalidator.org/service/validate/), | p.Gln808Lys | NP_000343.2  [NM_000352.4](https://www.ncbi.nlm.nih.gov/nuccore/NM_000352.4) | 0.00005302 | USA | VUS |  |  |
|  | *ABCC8* | c.2506C>T  c.4138_4140delinsCA | p.Arg836Ter p.Thr1380Glnfs*80 | [NM_000352.4](https://www.ncbi.nlm.nih.gov/nuccore/NM_000352.4)  NP_000343.2 |  | USA | P |  |  |
|  | *ABCC8* | [c.4138_4140delinsCA](https://variantvalidator.org/service/validate/) | p.Thr1380GlnfsTer80 | NM_000352.4  [NP_000343.2](https://www.ncbi.nlm.nih.gov/nuccore/NP_000343.2) |  |  |  |  |  |
|  | *ABCC8* | c.4543A>G | p.Thr1515Ala | NP_000343.2 [NM_000352.4](https://www.ncbi.nlm.nih.gov/nuccore/NM_000352.4)  (25555642) |  | USA | VUS |  |  |
|  | *ABCC8* | c.4169C>T | p.Ala1390Val | NP_000343.2 [NM_000352.4](https://www.ncbi.nlm.nih.gov/nuccore/NM_000352.4)  (21378087) |  | Australia | VUS | 2018 | ([188](#_ENREF_188)) |
|  | *ABCC8* | c.307C>T | p.His103Tyr | NP_001274103.1  [NM_000352.4](https://www.ncbi.nlm.nih.gov/nuccore/NM_000352.4) | 0.0000358 | Korea | VUS | 2016 | ([115](#_ENREF_115)) |
|  | *ABCC8* | c.221G>A | p.Arg74Gln | NP_001274103.1  [NM_000352.4](https://www.ncbi.nlm.nih.gov/nuccore/NM_000352.4)  (9618169) | 0.0000079 | Korea | VUS |  |  |
|  | *ABCC8* | c.1880A>G | p.His627Arg | NP_001274103.1  [NM_000352.4](https://www.ncbi.nlm.nih.gov/nuccore/NM_000352.4) | 0.0000176 | Russia | VUS | 2019 | ([125](#_ENREF_125)) |
|  | *ABCC8* | c.4096G>A | p.Ala1366Thr | NP_000343.2 [NM_000352.4](https://www.ncbi.nlm.nih.gov/nuccore/NM_000352.4)  (22662265) |  | Norway | VUS | 2012 | ([157](#_ENREF_157)) |
|  | *ABCC8* | c.4058G>A | p.Arg1353His | NP_001274103.1 |  | Greece | LP | 2019 | ([189](#_ENREF_189)) |
|  | *ABCC8* | c.1819G>A | p.Val607Met | NP_001274103.1 [NM_000352.4](https://www.ncbi.nlm.nih.gov/nuccore/NM_000352.4)  (18025408) | 8.00371372316755E-06 | Japan | VUS | 2018 | ([190](#_ENREF_190)) |
|  | *PDX1* | c.164G>A | p.Gly55Asp | NP_000200.1  (26226118) | 0.00013 | Turkey |  | 2015 | ([5](#_ENREF_5)) |
|  | *PDX1* | c.713_714ins | p.L238 ins P | NP_000200.1 |  | Canada |  | 2016 | ([7](#_ENREF_7)) |
|  | *PDX1* | c.A571C | p.lys191Gln | NP_000200.1 [NM_000209.3](https://www.ncbi.nlm.nih.gov/nuccore/NM_000209.3) |  | Canada | LP |  |  |
|  | *PDX1* | c.529G>A | p.Val177Met | [NM_000209.3](https://www.ncbi.nlm.nih.gov/nuccore/NM_000209.3) [NP_000200.1](https://www.ncbi.nlm.nih.gov/nuccore/NP_000200.1)  (25041077) |  | India | VUS | 2015 | ([93](#_ENREF_93)) |
|  | *PDX1* | c.670G>A, | p.Glu224Lys | [NM_000209.3](https://www.ncbi.nlm.nih.gov/nuccore/NM_000209.3) [NP_000200.1](https://www.ncbi.nlm.nih.gov/nuccore/NP_000200.1)  (14764823) | 0.001139 | India | VUS |  |  |
|  | *PDX1* | c.188delC | p.Pro63ArgfsTer60 | NP_000200.1  [NM_000209.3](https://www.ncbi.nlm.nih.gov/nuccore/NM_000209.3) | 0.000014461525 | Brazil |  | 2018 | ([191](#_ENREF_191)) |
|  | *PDX1* | c.664G>A | p.Glu222Lys | NP_000200.1  [NM_000209.3](https://www.ncbi.nlm.nih.gov/nuccore/NM_000209.3) | 0.0000183 | Brazil | VUS | 2019 | ([68](#_ENREF_68)) |
|  | *PDX1* | c.463C>A | p.Arg155Ser | NP_000200.1  [NM_000209.3](https://www.ncbi.nlm.nih.gov/nuccore/NM_000209.3) | 0.000074581 | China | VUS | 2019 | ([192](#_ENREF_192)) |
|  | *PDX1* | c.670 G>A | p.Glu224Lys | NP_000200.1  [NM_000209.3](https://www.ncbi.nlm.nih.gov/nuccore/NM_000209.3)  (14764823) | 0.001139 | India | VUS | 2017 | ([193](#_ENREF_193)) |
|  | *PDX1* | c.694G>A | p.Gly232Ser | NP_000200.1 | 0.0000461 | USA |  | 2015 | ([194](#_ENREF_194)) |
|  | *NEUROD1* | c.175 G>C | p.Glu59Gln | [NM_002500.4](https://www.ncbi.nlm.nih.gov/nuccore/NM_002500.4)  [NP_002491.2](https://www.ncbi.nlm.nih.gov/nuccore/NP_002491.2)  (25041077) | 0.0000434 | India | VUS | 2015 | ([93](#_ENREF_93)) |
|  | *NEUROD1* | c.-162G>A |  | [NM_002500.4](https://www.ncbi.nlm.nih.gov/nuccore/NM_002500.4) [NP_002491.2](https://www.ncbi.nlm.nih.gov/nuccore/NP_002491.2) | NA | India |  |  |  |
|  | *NEUROD1* | c.616_617insC, | p.His206ProfsTer38 | NP_002491.2  [NM_002500.4](https://www.ncbi.nlm.nih.gov/nuccore/NM_002500.4) | NA | Japan |  | 2018 | ([195](#_ENREF_195)) |
|  | *NEUROD1* | c.734delC | p.Pro245ArgfsTer17 | NP_002491.2 [NM_002500.4](https://www.ncbi.nlm.nih.gov/nuccore/NM_002500.4) | NA | Japan |  |  |  |
|  | *NEUROD1* |  | p.Leu157Arg | NP_002491.2 | NA | Japan |  |  |  |
|  | *NEUROD1* | c.616delC | p.His206ThrfsTer56 | NP_002491.2  [NM_002500.4](https://www.ncbi.nlm.nih.gov/nuccore/NM_002500.4) | NA | Japan |  |  |  |
|  | *NEUROD1* | c.308G > C | p.Arg103Pro | NP_002491.2 [NM_002500.4](https://www.ncbi.nlm.nih.gov/nuccore/NM_002500.4)  (26773576) | NA | Poland |  | 2016 | ([196](#_ENREF_196)) |
|  | *NEUROD1* | c.1972G>A |  | ? | NA | Thailand |  | 2009 | ([44](#_ENREF_44)) |
|  | *NEUROD1* |  | p.Ala322Asn | NP_002491.2 | NA | Thailand |  |  |  |
|  | *NEUROD1* | c.451T>C | p.Trp151Arg | NP_002491.2  [NM_002500.4](https://www.ncbi.nlm.nih.gov/nuccore/NM_002500.4) | 0.0000565 | China | VUS | 2019 | ([61](#_ENREF_61)) |
|  | *NEUROD1* |  | p.Phe256Leufs*2 | NP_002491.2 | NA | Brazil |  | 2019 | ([197](#_ENREF_197)) |
|  | *NEUROD1* | c.693C>G | p.Tyr231Ter | NP_002491.2  [NM_002500.4](https://www.ncbi.nlm.nih.gov/nuccore/NM_002500.4) | NA | Brazil | VUS | 2019 | ([68](#_ENREF_68)) |
|  | *NEUROD1* | c.616dup | p.His206ProfsTer38 | [NM_002500.4](https://www.ncbi.nlm.nih.gov/nuccore/NM_002500.4) [NP_002491.2](https://www.ncbi.nlm.nih.gov/nuccore/NP_002491.2) | NA | UK |  | 2017 | ([140](#_ENREF_140)) |
|  | *PAX4* | IVS7-1G>A |  | ? | NA | Thailand |  | 2016 | ([198](#_ENREF_198)) |
|  | *PAX4* | c.92G>T | p.Arg31Leu | [NM_006193.2](https://www.ncbi.nlm.nih.gov/nuccore/NM_006193.2) [NP_006184.2](https://www.ncbi.nlm.nih.gov/nuccore/NP_006184.2)  (25041077) | 0.00041 | Canada | VUS | 2016 | ([7](#_ENREF_7)) |
|  | *PAX4* | c.G290A | p.Arg97His | [NM_006193.2](https://www.ncbi.nlm.nih.gov/nuccore/NM_006193.2)  [NP_006184.2](https://www.ncbi.nlm.nih.gov/nuccore/NP_006184.2) | 0.0000318 | Canada | VUS |  |  |
|  | *PAX4* | c.92G>T | p.Arg31Leu | [NM_006193.2](https://www.ncbi.nlm.nih.gov/nuccore/NM_006193.2)  [NP_006184.2](https://www.ncbi.nlm.nih.gov/nuccore/NP_006184.2)  (25041077) | 0.00041 | India | VUS | 2015 | ([93](#_ENREF_93)) |
|  | *PAX 4* | c.490C>T | p.Arg164Trp | ?  (17426099) | 0.0000495 | Thailand |  | 2007 | ([199](#_ENREF_199)) |
|  | *PAX4* | c.574C > A | p.Arg192Ser | [NM_006193.2](https://www.ncbi.nlm.nih.gov/nuccore/NM_006193.2) [NP_006184.2](https://www.ncbi.nlm.nih.gov/nuccore/NP_006184.2) | 0.0027682 | China | Likely benign | 2019 | ([61](#_ENREF_61)) |
|  | *PAX4* | c.92G>A | p.Arg31Gln | [NM_006193.2](https://www.ncbi.nlm.nih.gov/nuccore/NM_006193.2) [NP_006184.2](https://www.ncbi.nlm.nih.gov/nuccore/NP_006184.2) | 0.003477 | China | Likely benign |  |  |
|  | *PAX4* | c.377A>G | p.Asp126Gly | [NM_006193.2](https://www.ncbi.nlm.nih.gov/nuccore/NM_006193.2)  [NP_006184.2](https://www.ncbi.nlm.nih.gov/nuccore/NP_006184.2) | NA | Russia | VUS | 2019 | ([125](#_ENREF_125)) |
|  | *PAX4* | c.55C>T | p.Arg19Trp | [NM_006193.2](https://www.ncbi.nlm.nih.gov/nuccore/NM_006193.2)  [NP_006184.2](https://www.ncbi.nlm.nih.gov/nuccore/NP_006184.2) | 7.95646223863021E-06 | Russia | VUS |  |  |
|  | *PAX4* | c.374-412 del 39 |  |  | NA | Japan |  | 2011 | ([200](#_ENREF_200)) |
|  | *PAX4* | c.593 C>T | p.Ala198Val | [NM_006193.2](https://www.ncbi.nlm.nih.gov/nuccore/NM_006193.2)  [NP_006184.2](https://www.ncbi.nlm.nih.gov/nuccore/NP_006184.2) | NA | Italy | VUS | 2018 | ([201](#_ENREF_201)) |
|  | *BLK* | [c.809C>T](https://variantvalidator.org/service/validate/) | p.Thr270Met | NP_001706.2 [NM_001715.2](https://www.ncbi.nlm.nih.gov/nuccore/NM_001715.2) | 0.0001060 | Canada | VUS | 2016 | ([7](#_ENREF_7)) |
|  | *BLK* | c.57G>A |  |  | NA | USA |  | 2009 | ([202](#_ENREF_202)) |
|  | *BLK* | c.211G>A | p.Ala71Thr | NP_001706.2  (19667185) | 0.011601 | USA |  |  |  |
|  | *BLK* | Chr8:11,459,364 T>G |  |  | NA | USA |  |  |  |
|  | *BLK* | Chr8:11,459,531 G>T |  |  | NA | USA |  |  |  |
|  | *BLK* | Chr8:11,468,050 C>T |  |  | NA | USA |  |  |  |
|  | *BLK* | c.338T>G | p.Val113Gly | NP_001706.2  NM_001715.2 | NA | Italy | VUS | 2018 | ([201](#_ENREF_201)) |
|  | *KCNJ11* | c.175G>A | p.Val59Met | NP_000516.3  [NM_000525.3](https://www.ncbi.nlm.nih.gov/nuccore/NM_000525.3)  (15115830) |  | Norway | VUS | 2013 | ([22](#_ENREF_22)) |
|  | *KCNJ11* | c.616C>T | p.Arg206Cys | NP_000516.3  [NM_000525.3](https://www.ncbi.nlm.nih.gov/nuccore/NM_000525.3)  (25555642) | 0.0000120 | USA | VUS | 2015 | ([25](#_ENREF_25)) |
|  | *KCNJ11* | c.691G>C | p.Val231Leu | NP_000516.3 [NM_000525.3](https://www.ncbi.nlm.nih.gov/nuccore/NM_000525.3)  (27118464) |  | USA | VUS |  |  |
|  | *KCNJ11* | c.970G>A | p.Gly324Arg | [NP_000516.3](https://www.ncbi.nlm.nih.gov/nuccore/NP_000516.3) [NM_000525.3](https://www.ncbi.nlm.nih.gov/nuccore/NM_000525.3)  (27118464) | 0.0000106 | USA | VUS |  |  |
|  | *KCNJ11* | c.85delC | p.Arg29AlafsTer101 | NP_000516.3 [NM_000525.3](https://www.ncbi.nlm.nih.gov/nuccore/NM_000525.3) | NA | Australia |  | 2019 | ([203](#_ENREF_203)) |
|  | *KLF11* |  | p.His418Gln | NP_003588.1 | NA | Japan |  | 2019 | ([204](#_ENREF_204)) |
|  | *KLF11* | c.604G>A | p.Glu202Lys | NP_003588.1  [NM_003597.4](https://www.ncbi.nlm.nih.gov/nuccore/NM_003597.4) | NA | China | VUS | 2019 | ([61](#_ENREF_61)) |
|  | *KLF11* | c.686T>G | p.Val229Gly | NP_003588.1 [NM_003597.4](https://www.ncbi.nlm.nih.gov/nuccore/NM_003597.4) | NA | China | VUS |  |  |
|  | *KLF11* | c.14A>G | p.Asp5Gly | NP_003588.1  [NM_003597.4](https://www.ncbi.nlm.nih.gov/nuccore/NM_003597.4) | 0.0000360 | China | VUS |  |  |
|  | *KLF11* | c.316A>C | p.Ile106Leu | NP_003588.1 [NM_003597.4](https://www.ncbi.nlm.nih.gov/nuccore/NM_003597.4) | 3.99176100528509E-06 | China | VUS |  |  |
|  | *APPL1* | c.1655T>A | p.Leu552Ter | NP_036228.1  [NM_012096.2](https://www.ncbi.nlm.nih.gov/nuccore/NM_012096.2)  (26073777) | NA | Italy | P | 2015 | ([205](#_ENREF_205)) |
|  | *APPL1* | c.280G>A | p.Asp94Asn | NP_036228.1 [NM_012096.2](https://www.ncbi.nlm.nih.gov/nuccore/NM_012096.2)  (15115830) | 4.03222554656817E-06 | Italy | VUS |  |  |
|  | *CEL* | c.1785delC | [p.Val596CysfsTer111](https://variantvalidator.org/service/validate/) | [NM_001807.4](https://www.ncbi.nlm.nih.gov/nuccore/NM_001807.4)  [NP_001798.2](https://www.ncbi.nlm.nih.gov/nuccore/NP_001798.2) | NA | Norway | VUS | 2013 | ([22](#_ENREF_22)) |
|  | *HNF1a* | [c.34C>T](https://variantvalidator.org/service/validate/) | p.Leu12Phe | NP_001293108.2  [NM_000545.6](https://www.ncbi.nlm.nih.gov/nuccore/NM_000545.6)  [NP_000536.5](https://www.ncbi.nlm.nih.gov/nuccore/NP_000536.5)  (15928245) | NA | Denmark | VUS | 2005 | ([28](#_ENREF_28)) |
|  | *HNF1a* | [c.370C>T](https://variantvalidator.org/service/validate/) | p.Gln124Ter | NP_001293108.2  [NP_000536.5](https://www.ncbi.nlm.nih.gov/nuccore/NP_000536.5) [NM_000545.6](https://www.ncbi.nlm.nih.gov/nuccore/NM_000545.6)  (15928245) | NA | Denmark | P |  |  |
|  | *HNF1a* | [c.686G>C](https://variantvalidator.org/service/validate/), | p.Arg229Pro | NP_001293108.2  [NP_000536.5](https://www.ncbi.nlm.nih.gov/nuccore/NP_000536.5) [NM_000545.6](https://www.ncbi.nlm.nih.gov/nuccore/NM_000545.6)  (11058894) | NA | Denmark | LP |  |  |
|  | *HNF1a* | [c.700G>T](https://variantvalidator.org/service/validate/) | p.Glu234Ter | NP_001293108.2 [NM_000545.6](https://www.ncbi.nlm.nih.gov/nuccore/NM_000545.6)  (11058894) |  | Denmark | P |  |  |
|  | *HNF1a* | c. 1636-1639delGACA GACACTdelGACAfs | p.Asp546Thr547fsdelGACA | NP_000536.6 | NA | Denmark |  |  |  |
|  | *HNF1a* | c. 1767-1768insA GTG-insAfs | p.Val590fsinsA | NP_000536.6 | NA | Denmark |  |  |  |
|  | *INS* | c.187+241G>A |  | [NM_001291897.1](https://www.ncbi.nlm.nih.gov/nuccore/NM_001291897.1)  [NP_001278826.1](https://www.ncbi.nlm.nih.gov/nuccore/NP_001278826.1) | NA | USA |  | 2015 | ([206](#_ENREF_206)) |
|  | *HNF1b* | c.983delC | p.Pro328Leufs*48 | [NM_000458.3](https://www.ncbi.nlm.nih.gov/nuccore/NM_000458.3)  [NP_000449.1](https://www.ncbi.nlm.nih.gov/nuccore/NP_000449.1) | NA | Brazil |  | 2019 | ([207](#_ENREF_207)) |
|  | *GCK* | c.92T>C | p.Leu31Pro | [NM_033507.1](https://www.ncbi.nlm.nih.gov/nuccore/NM_033507.1)  [NP_277042.1](https://www.ncbi.nlm.nih.gov/nuccore/NP_277042.1) | NA | Korea | LP | 2017 | ([208](#_ENREF_208)) |
|  | *GCK* | c.1151C>T | p.Ser383Pro | NP_000153.1 | NA | Korea | LP |  |  |
|  | *GCK* |  | p.Gly178Ala | NP_000153.1 | NA | Brazil |  | 2017 | ([209](#_ENREF_209)) |
|  | *HNF1a* | c.815G>A | p.Arg272His | [NM_000545.6](https://www.ncbi.nlm.nih.gov/nuccore/NM_000545.6)  [NP_000536.5](https://www.ncbi.nlm.nih.gov/nuccore/NP_000536.5) | NA | Netherland | VUS | 2019 | ([210](#_ENREF_210)) |
|  | *HNF1b* | c.276C>T | p.Arg276X | [NM_001165923.3](https://www.ncbi.nlm.nih.gov/nuccore/NM_001165923.3)  [NP_001159395.1](https://www.ncbi.nlm.nih.gov/nuccore/NP_001159395.1)  (12161522) |  | Japan | Likely benign | 2007 | ([173](#_ENREF_173)) |
|  | *HNF1a* | Gly288fs*863_864insC | p.Pro289AlafsTer28 | [NM_000545.6](https://www.ncbi.nlm.nih.gov/nuccore/NM_000545.6)  [NP_000536.5](https://www.ncbi.nlm.nih.gov/nuccore/NP_000536.5) |  | Denmark |  | 2017 | ([211](#_ENREF_211)) |
|  | *HNf4a* | 992G>A | p.Arg331His | [NM_178849.2](https://www.ncbi.nlm.nih.gov/nuccore/NM_178849.2)  [NP_849180.1](https://www.ncbi.nlm.nih.gov/nuccore/NP_849180.1) | 4.02197607728629E-06 | Denmark | VUS |  |  |
|  | *HNf4a* | c.256C>T | p.Gln86X | [NM_001287182.1](https://www.ncbi.nlm.nih.gov/nuccore/NM_001287182.1)  [NP_001274111.1](https://www.ncbi.nlm.nih.gov/nuccore/NP_001274111.1) | NA | USA | P | 2018 | ([212](#_ENREF_212)) |
|  | *HNF4a* | c.987 1003del17ins9 | p.Leu330fs | NP_001245284.1 | NA | UK |  | 2008 | ([213](#_ENREF_213)) |
|  | *HNF4a* | c.264–21A>G |  |  |  | UK |  |  |  |
|  | *HNF4a* | c.48C>G | p.Tyr16X | [NM_001287182.1](https://www.ncbi.nlm.nih.gov/nuccore/NM_001287182.1)  [NP_001274111.1](https://www.ncbi.nlm.nih.gov/nuccore/NP_001274111.1)  (17407387) |  | UK | P |  |  |
|  | *HNf1a* |  | p.Pro611Leu | XP_024304936.1 |  | India |  | 2018 | ([214](#_ENREF_214)) |
|  | *HNf1a* |  | p.Val648Ile | XP_024304936.1 |  | India |  |  |  |
|  | *HNf1a* | c.1061C>T | p.Thr354Met | XP_024304936.1  (11058894) | 0.0000636 | India |  |  |  |
|  | *HNf1a* |  | p.Trp113* | XP_024304936.1  (11692182) | NA | India |  |  |  |
|  | *ABCC8* |  | p.lys1023Gln | NP_001274103.1 |  | India |  |  |  |
|  | *ABCC8* | c.2912A>T | p.Glu971Val | NP_001274103.1 | 0.00000397 | India |  |  |  |
|  | *ABCC8* |  | p.Ala1473Thr | NP_001274103.1 |  | India |  |  |  |
|  | *ABCC8* |  | p.Gly1009Ser | NP_001274103.1 |  | India |  |  |  |
|  | *ABCC8* |  | p.Asn781Ser | NP_001274103.1 |  | India |  |  |  |
|  | *BLK* |  | p.Arg446Gly | NP_001706.2 |  | India |  |  |  |
|  | *CEL* | c.294C>G | p.tyr98* | ? | 4.00936587869262E-06 | India |  |  |  |
|  | *GCK* |  | p.Thr207Ala | NP_277042.1 | NA | India |  |  |  |
|  | *HNF1b* | c.1306A>G | p.Thr436Ala | NP_000449.1 | 3.97630124458229E-06 | India |  |  |  |
|  | *KCNJ11* | c.1084G>A | p.Ala362Thr | NP_000516.3 | 0.000023898 | India |  |  |  |
|  | *KLF11* | c.1394G>A | p.Arg465His | NP_003588 | 0.00001193 | India |  |  |  |
|  | *PDX1* |  | p.lys147Arg | NP_000200.1 |  | India |  |  |  |
|  | *ABCC8* |  | p.Ala1457Thr | NP_000343.2  (12364426) |  | Russia |  | 2016 | ([215](#_ENREF_215)) |
|  | *HNF1b* | c.374T>C | p.Ile125Thr | [NM_001165923.3](https://www.ncbi.nlm.nih.gov/nuccore/NM_001165923.3)  [NP_001159395.1](https://www.ncbi.nlm.nih.gov/nuccore/NP_001159395.1)  (22432796) |  | France | VUS | 2012 | ([216](#_ENREF_216)) |
|  | *HNF1b* | c.544+1_544+4del |  | [NM_001165923.3](https://www.ncbi.nlm.nih.gov/nuccore/NM_001165923.3)  [NP_001159395.1](https://www.ncbi.nlm.nih.gov/nuccore/NP_001159395.1) |  | France |  |  |  |
|  | *HNF1b* | c.698G>A | p.Arg233His | [NM_000458.3](https://www.ncbi.nlm.nih.gov/nuccore/NM_000458.3)  [NP_000449.1](https://www.ncbi.nlm.nih.gov/nuccore/NP_000449.1)  (22432796) |  | France | VUS |  |  |
|  | *HNF4a* | c.268C>T | p.Gln268Ser |  |  | USA |  | 1996 | ([217](#_ENREF_217)) |
|  | *GCK* |  | p.Val406Ala | NP_000153.1 |  | Poland |  | 2014 | ([218](#_ENREF_218)) |
|  | *GCK* |  | p.Arg447Gln | NP_000153.1 |  | Poland |  |  |  |
|  | *GCK* |  | p.Arg43Cys | NP_000153.1  (17573900) |  | Poland |  |  |  |
|  | *GCK* |  | p.Arg337Cys | ? |  | Poland |  |  |  |
|  | *GCK* |  | p.Gly318Arg | NP_000153.1 |  | Poland |  |  |  |
|  | *GCK* |  | p.Phe150fs | NP_000153.1 |  | Poland |  |  |  |
|  | *GCK* |  | p.Arg186X | NP_000153.1 |  | Poland |  |  |  |
|  | *GCK* |  | p.Gly318Arg | NP_000153.1  (12627330) | NA | Poland |  |  |  |
|  | *ABCC8* | c.1763C>T | p.Thr588Ile | NP_000343.2 | NA | Finland | VUS | 2016 | ([219](#_ENREF_219)) |
|  | *GCK* | c.635_637delCCT | p.Ser212 | NP_000153.1 | NA | Finland |  |  |  |
|  | *GCK* | c.364-8T>G |  | [NM_033508.1](https://www.ncbi.nlm.nih.gov/nuccore/NM_033508.1)  [NP_277043.1](https://www.ncbi.nlm.nih.gov/nuccore/NP_277043.1) | NA | Iran |  | 2019 | ([220](#_ENREF_220)) |
|  | *GCK* |  | p.Arg308lys | NP_000153.1 | NA | India |  | 2014 | ([221](#_ENREF_221)) |
|  | *GCK* | c.1278_1286dup9, | p.Arg427_Leu429dup | [NM_033508.2](https://www.ncbi.nlm.nih.gov/nuccore/NM_033508.2)  [NP_277043.1](https://www.ncbi.nlm.nih.gov/nuccore/NP_277043.1) | NA | Japan |  | 2012 | ([222](#_ENREF_222)) |
|  | *GCK* | c.1142T>G | p.Met381Arg | [NM_000162.4](https://www.ncbi.nlm.nih.gov/nuccore/NM_000162.4)  [NP_000153.1](https://www.ncbi.nlm.nih.gov/nuccore/NP_000153.1) | NA | Japan | LP |  |  |
|  | *GCK* | c.175C>T | p.Pro59Ser | [NM_000162.4](https://www.ncbi.nlm.nih.gov/nuccore/NM_000162.4)  [NP_000153.1](https://www.ncbi.nlm.nih.gov/nuccore/NP_000153.1)  (22060211) | NA | Japan | LP |  |  |
|  | *GCK* | c.182A>G | p.Tyr61Cys | [NM_000162.4](https://www.ncbi.nlm.nih.gov/nuccore/NM_000162.4)  [NP_000153.1](https://www.ncbi.nlm.nih.gov/nuccore/NP_000153.1)  (22060211) | NA | Japan | VUS |  |  |
|  | *GCK* | c.576G>T | p.Gly193Trp | [NM_033508.2](https://www.ncbi.nlm.nih.gov/nuccore/NM_033508.2)  [NP_277043.1](https://www.ncbi.nlm.nih.gov/nuccore/NP_277043.1)  (24804978) | NA | Japan | VUS |  |  |
|  | *GCK* | c.500G>A | p.Trp167Ter | [NM_000162.4](https://www.ncbi.nlm.nih.gov/nuccore/NM_000162.4)  [NP_000153.1](https://www.ncbi.nlm.nih.gov/nuccore/NP_000153.1)  (24804978) | NA | Japan | P |  |  |
|  | *HNF1a* | c.1043T>C | p.Leu348Pro | [NM_000545.6](https://www.ncbi.nlm.nih.gov/nuccore/NM_000545.6)  [NP_000536.5](https://www.ncbi.nlm.nih.gov/nuccore/NP_000536.5)  (24804978) | NA | Japan | VUS |  |  |
|  | *HNF1a* | c.1054delT | [p.Ser352ProfsTer12](https://variantvalidator.org/service/validate/) | [NM_000545.6](https://www.ncbi.nlm.nih.gov/nuccore/NM_000545.6)  [NP_000536.5](https://www.ncbi.nlm.nih.gov/nuccore/NP_000536.5) | NA | Japan |  |  |  |
|  | *HNF1b* | c.395A>C | p.His132Pro | [NM_001165923.3](https://www.ncbi.nlm.nih.gov/nuccore/NM_001165923.3)  [NP_001159395.1](https://www.ncbi.nlm.nih.gov/nuccore/NP_001159395.1)  (24804978) |  | Japan | VUS |  |  |
|  | *HNF4a* | c.915_916insT | p.Arg306SerfsTer37 | [NM_001287184.1](https://www.ncbi.nlm.nih.gov/nuccore/NM_001287184.1)  [NP_001274113.1](https://www.ncbi.nlm.nih.gov/nuccore/NP_001274113.1) |  | Japan |  |  |  |
|  | *HNF4a* | c.970C>T | p.Arg324Cys | NP_001245284.1 |  | Japan | LP |  |  |
|  | *GCK* | c.492_494delTCT | p.Leu165del | [NM_000162.4](https://www.ncbi.nlm.nih.gov/nuccore/NM_000162.4)  [NP_000153.1](https://www.ncbi.nlm.nih.gov/nuccore/NP_000153.1) |  | Japan |  | 2018 | ([223](#_ENREF_223)) |
|  | *GCK* | c.533G>C | p.Gly178Ala | [NM_000162.4](https://www.ncbi.nlm.nih.gov/nuccore/NM_000162.4)  [NP_000153.1](https://www.ncbi.nlm.nih.gov/nuccore/NP_000153.1) |  | Japan | LP |  |  |
|  | *GCK* | c.538A>C | p.Asn180His | [NM_000162.4](https://www.ncbi.nlm.nih.gov/nuccore/NM_000162.4)  [NP_000153.1](https://www.ncbi.nlm.nih.gov/nuccore/NP_000153.1) |  | Japan | VUS |  |  |
|  | *GCK* | c.577G>T | p.Gly193Trp | [NM_000162.4](https://www.ncbi.nlm.nih.gov/nuccore/NM_000162.4)  [NP_000153.1](https://www.ncbi.nlm.nih.gov/nuccore/NP_000153.1)  (24804978) |  | Japan | VUS |  |  |
|  | *GCK* | c.671T>A | p.Met224Lys | [NM_000162.4](https://www.ncbi.nlm.nih.gov/nuccore/NM_000162.4)  [NP_000153.1](https://www.ncbi.nlm.nih.gov/nuccore/NP_000153.1) |  | Japan | VUS |  |  |
|  | *GCK* | c.707A>C | p.Glu236Ala | [NM_000162.4](https://www.ncbi.nlm.nih.gov/nuccore/NM_000162.4)  [NP_000153.1](https://www.ncbi.nlm.nih.gov/nuccore/NP_000153.1) |  | Japan | VUS |  |  |
|  | *GCK* | c.836_837delAG | p.Glu279Efs | [NM_000162.4](https://www.ncbi.nlm.nih.gov/nuccore/NM_000162.4) [NP_000153.1](https://www.ncbi.nlm.nih.gov/nuccore/NP_000153.1) |  | Japan |  |  |  |
|  | *GCK* | c.898G>T | p.Glu300* | [NM_000162.4](https://www.ncbi.nlm.nih.gov/nuccore/NM_000162.4)  [NP_000153.1](https://www.ncbi.nlm.nih.gov/nuccore/NP_000153.1)  (24804978) |  | Japan | P |  |  |
|  | *GCK* | c.1055T>G | p.Leu352Arg | [NM_000162.4](https://www.ncbi.nlm.nih.gov/nuccore/NM_000162.4)  [NP_000153.1](https://www.ncbi.nlm.nih.gov/nuccore/NP_000153.1)  (24804978) |  | Japan | VUS |  |  |
|  | *GCK* | c.1144_1149dupTGCTCG | p.Cys382_Ser383dup | [NM_000162.4](https://www.ncbi.nlm.nih.gov/nuccore/NM_000162.4)  [NP_000153.1](https://www.ncbi.nlm.nih.gov/nuccore/NP_000153.1) |  | Japan |  |  |  |
|  | *GCK* | c.1278_1286dupCGTGCGCAG | p.Ser426_Arg428dup | [NM_000162.4](https://www.ncbi.nlm.nih.gov/nuccore/NM_000162.4)  [NP_000153.1](https://www.ncbi.nlm.nih.gov/nuccore/NP_000153.1) |  | Japan |  |  |  |
|  | *GCK* | c.1340_1368del29 | p.Arg447LeufsTer2 | [NM_000162.4](https://www.ncbi.nlm.nih.gov/nuccore/NM_000162.4)  [NP_000153.1](https://www.ncbi.nlm.nih.gov/nuccore/NP_000153.1) |  | Japan |  |  |  |
|  | *HNF1a* | c.618G>A | p.Trp206Ter | [NM_000545.6](https://www.ncbi.nlm.nih.gov/nuccore/NM_000545.6)  [NP_000536.5](https://www.ncbi.nlm.nih.gov/nuccore/NP_000536.5)  (24642958) |  | Japan | P |  |  |
|  | *HNF1a* | c.752C>T | p.Ala251Val | [NM_000545.6](https://www.ncbi.nlm.nih.gov/nuccore/NM_000545.6)  [NP_000536.5](https://www.ncbi.nlm.nih.gov/nuccore/NP_000536.5) |  | Japan | VUS |  |  |
|  | *HNF1a* | c.778A>T | p.Thr260Ser | [NM_000545.6](https://www.ncbi.nlm.nih.gov/nuccore/NM_000545.6)  [NP_000536.5](https://www.ncbi.nlm.nih.gov/nuccore/NP_000536.5) |  | Japan | VUS |  |  |
|  | *HNF1a* | c.791T>C | p.Val264Ala | [NM_000545.6](https://www.ncbi.nlm.nih.gov/nuccore/NM_000545.6)  [NP_000536.5](https://www.ncbi.nlm.nih.gov/nuccore/NP_000536.5) |  | Japan | VUS |  |  |
|  | *HNF1b* | c.395A>C | p.His132Pro | [NM_001165923.3](https://www.ncbi.nlm.nih.gov/nuccore/NM_001165923.3)  [NP_001159395.1](https://www.ncbi.nlm.nih.gov/nuccore/NP_001159395.1)  (22060211) |  | Japan | VUS |  |  |
|  | *HNF4a* | c.146A>C | p.His49Pro | [NM_001287184.1](https://www.ncbi.nlm.nih.gov/nuccore/NM_001287184.1)  [NP_001274113.1](https://www.ncbi.nlm.nih.gov/nuccore/NP_001274113.1) |  | Japan | LP |  |  |
|  | *HNF4a* | c.426+1G>A |  | ? |  | Japan |  |  |  |
|  | *HNF4a* | c.857T>A | p.Ile286Asn | [NM_001287184.1](https://www.ncbi.nlm.nih.gov/nuccore/NM_001287184.1)  [NP_001274113.1](https://www.ncbi.nlm.nih.gov/nuccore/NP_001274113.1) |  | Japan | LP |  |  |
|  | *HNF4a* | c.874C>T | p.Gln292* | NP_787110.2 |  | Japan | P |  |  |
|  | *PDX1* | c.302C>T | p.Pro101Leu | NP_000200.1 | 0.00001536 | India |  | 2019 | ([224](#_ENREF_224)) |
|  | *GCK* | c.70G>A |  | NP_000153.1 | NA | France | P | 1993 | ([225](#_ENREF_225)) |
|  | *GCK* | c.98C>T |  | NP_000153.1 | NA | France |  |  |  |
|  | *GCK* | c.161-15bpdeletion |  |  | NA | France |  |  |  |
|  | *GCK* | c.175G>A |  | NP_000153.1 | NA | France | LP |  |  |
|  | *GCK* | c.182G>A |  | NP_000153.1 |  | France | VUS |  |  |
|  | *GCK* | c.186C>T | p.Tyr62Tyr | NP_000153.1 | 0.00003537 | France | Likely benign |  |  |
|  | *GCK* | c.203T>C |  | NP_000153.1 | NA | France | VUS |  |  |
|  | *GCK* | c.227G>T |  |  |  | France |  |  |  |
|  | *GCK* | c.228C>T |  | NP_000153.1 |  | France | Likely benign |  |  |
|  | *GCK* | c.261G>A |  | NP_000153.1 |  | France | VUS |  |  |
|  | *GCK* | c.279G>T |  | NP_000153.1 |  | France |  |  |  |
|  | *GCK* | c.300G>A |  | NP_000153.1 |  | France | Likely benign |  |  |
|  | *GCK* | c.300G>C |  | NP_000153.1 |  | France | VUS |  |  |
|  | *GCK* | c.309T>C |  | NP_000153.1 |  | France |  |  |  |
|  | *GCK* | c.414A>G |  | NP_000153.1 |  | France | Likely benign |  |  |
|  | *HNF1b* | heterozygous deletion |  |  |  | New Zealand |  | 2017 | ([226](#_ENREF_226)) |
|  | *HNF1b* | *HNF1b* deletion |  |  |  | USA |  | 2019 | ([227](#_ENREF_227)) |
|  | *GCK* | c.1116G>C | p.Glu372Asp | NP_000153.1 | NA | Italy | VUS | 2019 | ([228](#_ENREF_228)) |
|  | *HNF1b* | *HNF1b* deletion |  |  |  | Japan |  | 2019 | ([229](#_ENREF_229)) |
|  | *HNF1b* | *HNF1b* deletion |  |  |  | UK |  | 2018 | ([230](#_ENREF_230)) |
|  | *PDX1* | Pro63fsdelC |  | NP_000200.1 |  | USA |  | 2000 | ([231](#_ENREF_231)) |
|  | *HNF1b* | *HNF1b* deletion |  |  |  | UK |  | 2013 | ([232](#_ENREF_232)) |
|  | *GCK* | GCK exon2 |  |  |  | UK |  | 2007 | ([233](#_ENREF_233)) |
|  | *HNF1a* | *Exon1* |  |  |  | UK |  |  |  |
|  | *HNF1a* | *Exon2-10* |  |  |  | UK |  |  |  |
|  | *HNF1a* | *Exon 1-10* |  |  |  | UK |  |  |  |
|  | *HNF1b* | *HNF1b* deletion |  |  |  | Germany |  | 2009 | ([234](#_ENREF_234)) |
|  | *HNF1b* | *HNF1b* deletion |  |  |  | Germany |  | 2018 | ([235](#_ENREF_235)) |
|  | *ABCC8* |  | p.Arg826Trp | NP_001274103.1 |  | Argentina |  | 2016 | ([236](#_ENREF_236)) |
|  | *HNF1a* | *HNF1α* ex2-3del mutation |  |  |  | New Zealand |  | 2013 | ([237](#_ENREF_237)) |
|  | *HNF1a* | c.1512C>A | p.Ser504Arg | [NM_000545.6](https://www.ncbi.nlm.nih.gov/nuccore/NM_000545.6)  [NP_000536.5](https://www.ncbi.nlm.nih.gov/nuccore/NP_000536.5) | 0.00000797 | China | VUS | 2020 | ([238](#_ENREF_238)) |
|  | *HNF1a* | c.956-1G>C |  | [NM_000545.6](https://www.ncbi.nlm.nih.gov/nuccore/NM_000545.6)  [NP_000536.5](https://www.ncbi.nlm.nih.gov/nuccore/NP_000536.5) |  |  |  |  |  |
|  | *HNF1a* | c. 347C>T | p.Ala116Val | [NM_000545.6](https://www.ncbi.nlm.nih.gov/nuccore/NM_000545.6) [NP_000536.5](https://www.ncbi.nlm.nih.gov/nuccore/NP_000536.5)  (11315828) | 3.97946595566874E-06 |  | VUS |  |  |
|  | *HNF1a* | c.1192C>G | p.Gln398Glu | [NM_000545.6](https://www.ncbi.nlm.nih.gov/nuccore/NM_000545.6) [NP_000536.5](https://www.ncbi.nlm.nih.gov/nuccore/NP_000536.5) |  |  | VUS |  |  |
|  | *HNF1a* | c.1136C>A | p.Pro379His | NP_000536.6  (15883474) | 0.000051 | Mexico |  | 2005 | ([239](#_ENREF_239)) |

1. Papadimitriou DT, Willems PJ, Bothou C, Karpathios T, Papadimitriou A. A novel heterozygous mutation in the glucokinase gene is responsible for an early-onset mild form of maturity-onset diabetes of the young, type 2. Diabetes & metabolism. 2015;41(4):342-3.

2. Milenkovic T, Zdravkovic D, Mitrovic K. [Novel glucokinase mutation in a boy with maturity-onset diabetes of the young]. Srpski arhiv za celokupno lekarstvo. 2008;136(9-10):542-4.

3. Costa A, Bescos M, Velho G, Chevre J, Vidal J, Sesmilo G, et al. Genetic and clinical characterisation of maturity-onset diabetes of the young in Spanish families. European journal of endocrinology. 2000;142(4):380-6.

4. Mota AJ, Bruggemann S, Costa FF. MODY 2: mutation identification and molecular ancestry in a Brazilian family. Gene. 2013;512(2):486-91.

5. Anik A, Catli G, Abaci A, Sari E, Yesilkaya E, Korkmaz HA, et al. Molecular diagnosis of maturity-onset diabetes of the young (MODY) in Turkish children by using targeted next-generation sequencing. Journal of pediatric endocrinology & metabolism : JPEM. 2015;28(11-12):1265-71.

6. Aloi C, Salina A, Minuto N, Tallone R, Lugani F, Mascagni A, et al. Glucokinase mutations in pediatric patients with impaired fasting glucose. Acta diabetologica. 2017;54(10):913-23.

7. Brahm AJ, Wang G, Wang J, McIntyre AD, Cao H, Ban MR, et al. Genetic Confirmation Rate in Clinically Suspected Maturity-Onset Diabetes of the Young. Canadian journal of diabetes. 2016;40(6):555-60.

8. Lopez AP, de Dios A, Chiesa I, Perez MS, Frechtel GD. Analysis of mutations in the glucokinase gene in people clinically characterized as MODY2 without a family history of diabetes. Diabetes research and clinical practice. 2016;118:38-43.

9. Aykut A, Karaca E, Onay H, Goksen D, Cetinkalp S, Eren E, et al. Analysis of the GCK gene in 79 MODY type 2 patients: A multicenter Turkish study, mutation profile and description of twenty novel mutations. Gene. 2018;641:186-9.

10. Bazalova Z, Rypackova B, Broz J, Brunerova L, Polak J, Rusavy Z, et al. Three novel mutations in MODY and its phenotype in three different Czech families. Diabetes research and clinical practice. 2010;88(2):132-8.

11. Almeida C, Silva SR, Garcia E, Leite AL, Teles A, Campos RA. A novel genetic mutation in a Portuguese family with GCK-MODY. Journal of pediatric endocrinology & metabolism : JPEM. 2014;27(1-2):129-33.

12. Costantini S, Malerba G, Contreas G, Corradi M, Marin Vargas SP, Giorgetti A, et al. Genetic and bioinformatics analysis of four novel GCK missense variants detected in Caucasian families with GCK-MODY phenotype. Clinical genetics. 2015;87(5):440-7.

13. Delvecchio M, Ludovico O, Bellacchio E, Stallone R, Palladino T, Mastroianno S, et al. MODY type 2 P59S GCK mutant: founder effect in South of Italy. Clinical genetics. 2013;83(1):83-7.

14. Dusatkova P, Pruhova S, Borowiec M, Vesela K, Antosik K, Lebl J, et al. Ancestral mutations may cause a significant proportion of GCK-MODY. Pediatric diabetes. 2012;13(6):489-98.

15. Stern E, Strihan C, Potievsky O, Nimri R, Shalitin S, Cohen O, et al. Four novel mutations, including the first gross deletion in TCF1, identified in HNF-4alpha, GCK and TCF1 in patients with MODY in Israel. Journal of pediatric endocrinology & metabolism : JPEM. 2007;20(8):909-21.

16. Tatsi EB, Kanaka-Gantenbein C, Scorilas A, Chrousos GP, Sertedaki A. Next generation sequencing targeted gene panel in Greek MODY patients increases diagnostic accuracy. Pediatric diabetes. 2020;21(1):28-39.

17. Estalella I, Rica I, Perez de Nanclares G, Bilbao JR, Vazquez JA, San Pedro JI, et al. Mutations in GCK and HNF-1alpha explain the majority of cases with clinical diagnosis of MODY in Spain. Clinical endocrinology. 2007;67(4):538-46.

18. Giuffrida FM, Calliari LE, Manna TD, Ferreira JG, Saddi-Rosa P, Kunii IS, et al. A novel glucokinase deletion (p.Lys32del) and five previously described mutations co-segregate with the phenotype of mild familial hyperglycaemia (MODY2) in Brazilian families. Diabetes research and clinical practice. 2013;100(2):e42-5.

19. Giuffrida FMA, Moises RS, Weinert LS, Calliari LE, Manna TD, Dotto RP, et al. Maturity-onset diabetes of the young (MODY) in Brazil: Establishment of a national registry and appraisal of available genetic and clinical data. Diabetes research and clinical practice. 2017;123:134-42.

20. Gozlan Y, Tenenbaum A, Shalitin S, Lebenthal Y, Oron T, Cohen O, et al. The glucokinase mutation p.T206P is common among MODY patients of Jewish Ashkenazi descent. Pediatric diabetes. 2012;13(6):e14-21.

21. Guazzini B, Gaffi D, Mainieri D, Multari G, Cordera R, Bertolini S, et al. Three novel missense mutations in the glucokinase gene (G80S; E221K; G227C) in Italian subjects with maturity-onset diabetes of the young (MODY). Mutations in brief no. 162. Online. Human mutation. 1998;12(2):136.

22. Irgens HU, Molnes J, Johansson BB, Ringdal M, Skrivarhaug T, Undlien DE, et al. Prevalence of monogenic diabetes in the population-based Norwegian Childhood Diabetes Registry. Diabetologia. 2013;56(7):1512-9.

23. McKinney JL, Cao H, Robinson JF, Metzger DL, Cummings E, Riddell DC, et al. Spectrum of HNF1A and GCK mutations in Canadian families with maturity-onset diabetes of the young (MODY). Clinical and investigative medicine Medecine clinique et experimentale. 2004;27(3):135-41.

24. McKinney J, Cao H, Behme MT, Mahon JL, Hegele RA. Maturity-onset diabetes of the young (MODY) mutation in type 2 diabetes and latent autoimmune diabetes of the adult. Diabetes care. 2003;26(12):3358-9.

25. Bennett JT, Vasta V, Zhang M, Narayanan J, Gerrits P, Hahn SH. Molecular genetic testing of patients with monogenic diabetes and hyperinsulinism. Molecular genetics and metabolism. 2015;114(3):451-8.

26. Solera J, Arias P, Aminoso C, Gonzalez-Casado I, Garre P, Herranz L, et al. Identification of eight new mutations in the GCK gene by DHPLC screening in a Spanish population. Diabetes research and clinical practice. 2009;85(1):20-3.

27. Hwang JS, Shin CH, Yang SW, Jung SY, Huh N. Genetic and clinical characteristics of Korean maturity-onset diabetes of the young (MODY) patients. Diabetes research and clinical practice. 2006;74(1):75-81.

28. Johansen A, Ek J, Mortensen HB, Pedersen O, Hansen T. Half of clinically defined maturity-onset diabetes of the young patients in Denmark do not have mutations in HNF4A, GCK, and TCF1. The Journal of clinical endocrinology and metabolism. 2005;90(8):4607-14.

29. Hager J, Blanche H, Sun F, Vaxillaire NV, Poller W, Cohen D, et al. Six mutations in the glucokinase gene identified in MODY by using a nonradioactive sensitive screening technique. Diabetes. 1994;43(5):730-3.

30. Kawakita R, Hosokawa Y, Fujimaru R, Tamagawa N, Urakami T, Takasawa K, et al. Molecular and clinical characterization of glucokinase maturity-onset diabetes of the young (GCK-MODY) in Japanese patients. Diabetic medicine : a journal of the British Diabetic Association. 2014;31(11):1357-62.

31. Knebel B, Jacob S, Boxberg CV, Muller-Wieland D, Kotzka J. A novel nonsense mutation in GCK exon 9 co-segregates with diabetes phenotype. Experimental and clinical endocrinology & diabetes : official journal, German Society of Endocrinology [and] German Diabetes Association. 2004;112(6):298-301.

32. Lehto M, Wipemo C, Ivarsson SA, Lindgren C, Lipsanen-Nyman M, Weng J, et al. High frequency of mutations in MODY and mitochondrial genes in Scandinavian patients with familial early-onset diabetes. Diabetologia. 1999;42(9):1131-7.

33. Weinert LS, Silveiro SP, Giuffrida FM, Cunha VT, Bulcao C, Calliari LE, et al. Three unreported glucokinase (GCK) missense mutations detected in the screening of thirty-two Brazilian kindreds for GCK and HNF1A-MODY. Diabetes research and clinical practice. 2014;106(2):e44-8.

34. Caetano LA, Jorge AA, Malaquias AC, Trarbach EB, Queiroz MS, Nery M, et al. Incidental mild hyperglycemia in children: two MODY 2 families identified in Brazilian subjects. Arquivos brasileiros de endocrinologia e metabologia. 2012;56(8):519-24.

35. Liu L, Liu Y, Ge X, Liu X, Chen C, Wang Y, et al. Insights into pathogenesis of five novel GCK mutations identified in Chinese MODY patients. Metabolism: clinical and experimental. 2018;89:8-17.

36. Loomba-Albrecht LA, Jame M, Bremer AA. A novel glucokinase gene mutation and its effect on glycemic/C-peptide fluctuations in a patient with maturity-onset diabetes of the young type 2. Diabetes research and clinical practice. 2010;87(3):e23-5.

37. Kocova M, Elblova L, Pruhova S, Lebl J, Dusatkova P. Novel glucokinase gene mutation in the first Macedonian family tested for MODY. Diabetes research and clinical practice. 2017;130:86-9.

38. Stoffel M, Patel P, Lo YM, Hattersley AT, Lucassen AM, Page R, et al. Missense glucokinase mutation in maturity-onset diabetes of the young and mutation screening in late-onset diabetes. Nature genetics. 1992;2(2):153-6.

39. Mantovani V, Salardi S, Cerreta V, Bastia D, Cenci M, Ragni L, et al. Identification of eight novel glucokinase mutations in Italian children with maturity-onset diabetes of the young. Human mutation. 2003;22(4):338.

40. Delvecchio M, Mozzillo E, Salzano G, Iafusco D, Frontino G, Patera PI, et al. Monogenic Diabetes Accounts for 6.3% of Cases Referred to 15 Italian Pediatric Diabetes Centers During 2007 to 2012. The Journal of clinical endocrinology and metabolism. 2017;102(6):1826-34.

41. Henderson M, Levy E, Delvin E, Losekoot M, Lambert M. Prevalence and clinical phenotype of the p.Val226Met glucokinase gene mutation in French Canadians in Quebec, Canada. Molecular genetics and metabolism. 2007;90(1):87-92.

42. Njolstad PR, Cockburn BN, Bell GI, Sovik O. A missense mutation, Val62Ala, in the glucokinase gene in a Norwegian family with maturity-onset diabetes of the young. Acta Paediatr. 1998;87(8):853-6.

43. Afonso P, Ferraria N, Carvalho A, Castro SV. Maturity onset diabetes of young type 2 due to a novel de novo GKC mutation. Arquivos brasileiros de endocrinologia e metabologia. 2014;58(7):772-5.

44. Plengvidhya N, Boonyasrisawat W, Chongjaroen N, Jungtrakoon P, Sriussadaporn S, Vannaseang S, et al. Mutations of maturity-onset diabetes of the young (MODY) genes in Thais with early-onset type 2 diabetes mellitus. Clinical endocrinology. 2009;70(6):847-53.

45. Pruhova S, Dusatkova P, Sumnik Z, Kolouskova S, Pedersen O, Hansen T, et al. Glucokinase diabetes in 103 families from a country-based study in the Czech Republic: geographically restricted distribution of two prevalent GCK mutations. Pediatric diabetes. 2010;11(8):529-35.

46. Li Q, Cao X, Qiu HY, Lu J, Gao R, Liu C, et al. A three-step programmed method for the identification of causative gene mutations of maturity onset diabetes of the young (MODY). Gene. 2016;588(2):141-8.

47. Barrio R, Bellanne-Chantelot C, Moreno JC, Morel V, Calle H, Alonso M, et al. Nine novel mutations in maturity-onset diabetes of the young (MODY) candidate genes in 22 Spanish families. The Journal of clinical endocrinology and metabolism. 2002;87(6):2532-9.

48. Ben Khelifa S, Martinez R, Dandana A, Khochtali I, Ferchichi S, Castano L. Maturity Onset Diabetes of the Young (MODY) in Tunisia: Low frequencies of GCK and HNF1A mutations. Gene. 2018;651:44-8.

49. Sagen JV, Bjorkhaug L, Molnes J, Raeder H, Grevle L, Sovik O, et al. Diagnostic screening of MODY2/GCK mutations in the Norwegian MODY Registry. Pediatric diabetes. 2008;9(5):442-9.

50. Santana LS, Caetano LA, Costa-Riquetto AD, Quedas EPS, Nery M, Collett-Solberg P, et al. Clinical application of ACMG-AMP guidelines in HNF1A and GCK variants in a cohort of MODY families. Clinical genetics. 2017;92(4):388-96.

51. Agladioglu SY, Aycan Z, Cetinkaya S, Bas VN, Onder A, Peltek Kendirci HN, et al. Maturity onset diabetes of youth (MODY) in Turkish children: sequence analysis of 11 causative genes by next generation sequencing. Journal of pediatric endocrinology & metabolism : JPEM. 2016;29(4):487-96.

52. Kanthimathi S, Jahnavi S, Balamurugan K, Ranjani H, Sonya J, Goswami S, et al. Glucokinase gene mutations (MODY 2) in Asian Indians. Diabetes technology & therapeutics. 2014;16(3):180-5.

53. Park SS, Jang SS, Ahn CH, Kim JH, Jung HS, Cho YM, et al. Identifying Pathogenic Variants of Monogenic Diabetes Using Targeted Panel Sequencing in an East Asian Population. The Journal of clinical endocrinology and metabolism. 2019.

54. Jha S, Siddiqui S, Waghdhare S, Dubey S, Krishna S, Subramanian K, et al. Identification of a novel glucokinase mutation in an Indian woman with GCK-MODY. The lancet Diabetes & endocrinology. 2016;4(4):302.

55. Ozdemir TR, Kirbiyik O, Dundar BN, Abaci A, Kaya OO, Catli G, et al. Targeted next generation sequencing in patients with maturity-onset diabetes of the young (MODY). Journal of pediatric endocrinology & metabolism : JPEM. 2018;31(12):1295-304.

56. Taylor RAM, Mackie A, Mogra R, Pinner J, Rajendran S, Ross GP. Caudal regression syndrome in a fetus of a glucokinase-maturity-onset diabetes of the young pregnancy. Diabetic medicine : a journal of the British Diabetic Association. 2019;36(2):252-5.

57. DellaManna T, Silva MR, Chacra AR, Kunii IS, Rolim AL, Furuzawa G, et al. Clinical follow-up of two Brazilian subjects with glucokinase-MODY (MODY2) with description of a novel mutation. Arquivos brasileiros de endocrinologia e metabologia. 2012;56(8):490-5.

58. Toaima D, Nake A, Wendenburg J, Praedicow K, Rohayem J, Engel K, et al. Identification of novel GCK and HNF1A/TCF1 mutations and polymorphisms in German families with maturity-onset diabetes of the young (MODY). Human mutation. 2005;25(5):503-4.

59. Velho G, Blanche H, Vaxillaire M, Bellanne-Chantelot C, Pardini VC, Timsit J, et al. Identification of 14 new glucokinase mutations and description of the clinical profile of 42 MODY-2 families. Diabetologia. 1997;40(2):217-24.

60. Cho YK, Cho EH, Choi HS, Kim SW. Novel deletion mutation in the glucokinase gene from a korean man with GCK-MODY phenotype and situs inversus. Diabetes research and clinical practice. 2018;143:263-6.

61. Ming-Qiang Z, Yang-Li D, Ke H, Wei W, Jun-Fen F, Chao-Chun Z, et al. Maturity onset diabetes of the young (MODY) in Chinese children: genes and clinical phenotypes. Journal of pediatric endocrinology & metabolism : JPEM. 2019;32(7):759-65.

62. Bansal V, Gassenhuber J, Phillips T, Oliveira G, Harbaugh R, Villarasa N, et al. Spectrum of mutations in monogenic diabetes genes identified from high-throughput DNA sequencing of 6888 individuals. BMC medicine. 2017;15(1):213.

63. Beer NL, Osbak KK, van de Bunt M, Tribble ND, Steele AM, Wensley KJ, et al. Insights into the pathogenicity of rare missense GCK variants from the identification and functional characterization of compound heterozygous and double mutations inherited in cis. Diabetes care. 2012;35(7):1482-4.

64. Haliloglu B, Hysenaj G, Atay Z, Guran T, Abali S, Turan S, et al. GCK gene mutations are a common cause of childhood-onset MODY (maturity-onset diabetes of the young) in Turkey. Clinical endocrinology. 2016;85(3):393-9.

65. Bonfig W, Hermanns S, Warncke K, Eder G, Engelsberger I, Burdach S, et al. GCK-MODY (MODY 2) Caused by a Novel p.Phe330Ser Mutation. ISRN pediatrics. 2011;2011:676549.

66. Capuano M, Garcia-Herrero CM, Tinto N, Carluccio C, Capobianco V, Coto I, et al. Glucokinase (GCK) mutations and their characterization in MODY2 children of southern Italy. PloS one. 2012;7(6):e38906.

67. Chambers C, Fouts A, Dong F, Colclough K, Wang Z, Batish SD, et al. Characteristics of maturity onset diabetes of the young in a large diabetes center. Pediatric diabetes. 2016;17(5):360-7.

68. de Santana LS, Caetano LA, Costa-Riquetto AD, Franco PC, Dotto RP, Reis AF, et al. Targeted sequencing identifies novel variants in common and rare MODY genes. Molecular genetics & genomic medicine. 2019;7(12):e962.

69. Fu J, Wang T, Liu J, Wang X, Li M, Xiao X. Birthweight correlates with later metabolic abnormalities in Chinese patients with maturity-onset diabetes of the young type 2. Endocrine. 2019;65(1):53-60.

70. Gasperikova D, Tribble ND, Stanik J, Huckova M, Misovicova N, van de Bunt M, et al. Identification of a novel beta-cell glucokinase (GCK) promoter mutation (-71G>C) that modulates GCK gene expression through loss of allele-specific Sp1 binding causing mild fasting hyperglycemia in humans. Diabetes. 2009;58(8):1929-35.

71. Komazec J, Zdravkovic V, Sajic S, Jesic M, Andjelkovic M, Pavlovic S, et al. The importance of combined NGS and MLPA genetic tests for differential diagnosis of maturity onset diabetes of the young. Endokrynologia Polska. 2019;70(1):28-36.

72. Lorini R, Klersy C, d'Annunzio G, Massa O, Minuto N, Iafusco D, et al. Maturity-onset diabetes of the young in children with incidental hyperglycemia: a multicenter Italian study of 172 families. Diabetes care. 2009;32(10):1864-6.

73. Lukasova P, Vcelak J, Vankova M, Vejrazkova D, Andelova K, Bendlova B. Screening of mutations and polymorphisms in the glucokinase gene in Czech diabetic and healthy control populations. Physiological research. 2008;57 Suppl 1:S99-108.

74. Estica RM, Seelenfreund HD, Durruty AP, Briones BG. [Finding of a new mutation in a Chilean family with monogenic diabetes. Report of one case]. Revista medica de Chile. 2018;146(7):929-32.

75. Matyka KA, Beards F, Appleton M, Ellard S, Hattersley A, Dunger DB. Genetic testing for maturity onset diabetes of the young in childhood hyperglycaemia. Archives of disease in childhood. 1998;78(6):552-4.

76. Noorian S, Sayarifard F, Farhadi E, Barbetti F, Rezaei N. GCK Mutation in a Child with Maturity Onset Diabetes of the Young, Type 2. Iranian journal of pediatrics. 2013;23(2):226-8.

77. Pihoker C, Gilliam LK, Ellard S, Dabelea D, Davis C, Dolan LM, et al. Prevalence, characteristics and clinical diagnosis of maturity onset diabetes of the young due to mutations in HNF1A, HNF4A, and glucokinase: results from the SEARCH for Diabetes in Youth. The Journal of clinical endocrinology and metabolism. 2013;98(10):4055-62.

78. Gandica RG, Chung WK, Deng L, Goland R, Gallagher MP. Identifying monogenic diabetes in a pediatric cohort with presumed type 1 diabetes. Pediatric diabetes. 2015;16(3):227-33.

79. Tarantino RM, Abreu GM, Fonseca ACP, Kupfer R, Pereira MFC, Campos Junior M, et al. MODY probability calculator for GCK and HNF1A screening in a multiethnic background population. Archives of endocrinology and metabolism. 2019.

80. Sagen JV, Odili S, Bjorkhaug L, Zelent D, Buettger C, Kwagh J, et al. From clinicogenetic studies of maturity-onset diabetes of the young to unraveling complex mechanisms of glucokinase regulation. Diabetes. 2006;55(6):1713-22.

81. Schnyder S, Mullis PE, Ellard S, Hattersley AT, Fluck CE. Genetic testing for glucokinase mutations in clinically selected patients with MODY: a worthwhile investment. Swiss medical weekly. 2005;135(23-24):352-6.

82. Marks SD, Couch RM. Identification of two new mutations in the glucokinase gene that result in maturity-onset diabetes of the young. Diabetes care. 2010;33(7):e94.

83. Shepherd M, Shields B, Hammersley S, Hudson M, McDonald TJ, Colclough K, et al. Systematic Population Screening, Using Biomarkers and Genetic Testing, Identifies 2.5% of the U.K. Pediatric Diabetes Population With Monogenic Diabetes. Diabetes care. 2016;39(11):1879-88.

84. Shoemaker AH, Zienkiewicz J, Moore DJ. Clinical assessment of HNF1A and GCK variants and identification of a novel mutation causing MODY2. Diabetes research and clinical practice. 2012;96(2):e36-9.

85. Tinto N, Zagari A, Capuano M, De Simone A, Capobianco V, Daniele G, et al. Glucokinase gene mutations: structural and genotype-phenotype analyses in MODY children from South Italy. PloS one. 2008;3(4):e1870.

86. Valentinova L, Beer NL, Stanik J, Tribble ND, van de Bunt M, Huckova M, et al. Identification and functional characterisation of novel glucokinase mutations causing maturity-onset diabetes of the young in Slovakia. PloS one. 2012;7(4):e34541.

87. Wang Z, Diao C, Liu Y, Li M, Zheng J, Zhang Q, et al. Identification and functional analysis of GCK gene mutations in 12 Chinese families with hyperglycemia. Journal of diabetes investigation. 2019;10(4):963-71.

88. Wheeler BJ, Patterson N, Love DR, Prosser D, Tomlinson P, Taylor BJ, et al. Frequency and genetic spectrum of maturity-onset diabetes of the young (MODY) in southern New Zealand. Journal of diabetes and metabolic disorders. 2013;12(1):46.

89. Li X, Ting TH, Sheng H, Liang CL, Shao Y, Jiang M, et al. Genetic and clinical characteristics of Chinese children with Glucokinase-maturity-onset diabetes of the young (GCK-MODY). BMC pediatrics. 2018;18(1):101.

90. Juszczak A, Pavic T, Vuckovic F, Bennett AJ, Shah N, Pape Medvidovic E, et al. Plasma Fucosylated Glycans and C-Reactive Protein as Biomarkers of HNF1A-MODY in Young Adult-Onset Nonautoimmune Diabetes. Diabetes care. 2019;42(1):17-26.

91. Lopez AP, Foscaldi SA, Perez MS, Rodriguez M, Traversa M, Puchulu FM, et al. HNF1 alpha gene coding regions mutations screening, in a Caucasian population clinically characterized as MODY from Argentina. Diabetes research and clinical practice. 2011;91(2):208-12.

92. Beijers HJ, Losekoot M, Odink RJ, Bravenboer B. Hepatocyte nuclear factor (HNF)1A and HNF4A substitution occurring simultaneously in a family with maturity-onset diabetes of the young. Diabetic medicine : a journal of the British Diabetic Association. 2009;26(11):1172-4.

93. Chapla A, Mruthyunjaya MD, Asha HS, Varghese D, Varshney M, Vasan SK, et al. Maturity onset diabetes of the young in India - a distinctive mutation pattern identified through targeted next-generation sequencing. Clinical endocrinology. 2015;82(4):533-42.

94. Fang C, Huang J, Huang Y, Chen L, Chen X, Hu J. A novel nonsense mutation of the HNF1alpha in maturity-onset diabetes of the young type 3 in Asian population. Diabetes research and clinical practice. 2015;109(2):e5-7.

95. Chevre JC, Hani EH, Boutin P, Vaxillaire M, Blanche H, Vionnet N, et al. Mutation screening in 18 Caucasian families suggest the existence of other MODY genes. Diabetologia. 1998;41(9):1017-23.

96. Eide SA, Raeder H, Johansson S, Midthjell K, Sovik O, Njolstad PR, et al. Prevalence of HNF1A (MODY3) mutations in a Norwegian population (the HUNT2 Study). Diabetic medicine : a journal of the British Diabetic Association. 2008;25(7):775-81.

97. Ellard S, Bulman MP, Frayling TM, Shepherd M, Hattersley AT. Proposed mechanism for a novel insertion/deletion frameshift mutation (I414G415ATCG-->CCA) in the hepatocyte nuclear factor 1 alpha (HNF-1 alpha) gene which causes maturity-onset diabetes of the young (MODY). Human mutation. 2000;16(3):273.

98. Karaca E, Onay H, Cetinkalp S, Aykut A, Goksen D, Ozen S, et al. The spectrum of HNF1A gene mutations in patients with MODY 3 phenotype and identification of three novel germline mutations in Turkish Population. Diabetes & metabolic syndrome. 2017;11 Suppl 1:S491-S6.

99. Globa E, Zelinska N, Elblova L, Dusatkova P, Cinek O, Lebl J, et al. MODY in Ukraine: genes, clinical phenotypes and treatment. Journal of pediatric endocrinology & metabolism : JPEM. 2017;30(10):1095-103.

100. Fehmann HC, Gross U, Epe M. A new mutation in the hepatocyte nuclear factor-1-alpha gene (P224S) in a newly discovered German family with maturity-onset diabetes of the young 3 (MODY 3). Family members carry additionally the homozygous I27L amino acid polymorphism in the HNF1 alpha gene. Experimental and clinical endocrinology & diabetes : official journal, German Society of Endocrinology [and] German Diabetes Association. 2004;112(2):84-7.

101. Horikawa Y, Enya M, Fushimi N, Fushimi Y, Takeda J. Screening of diabetes of youth for hepatocyte nuclear factor 1 mutations: clinical phenotype of HNF1beta-related maturity-onset diabetes of the young and HNF1alpha-related maturity-onset diabetes of the young in Japanese. Diabetic medicine : a journal of the British Diabetic Association. 2014;31(6):721-7.

102. Iwabuchi A, Kamoda T, Shinohara H, Sumazaki R. Japanese boy with maturity-onset diabetes of the young type 3 who developed diabetes at 19 months old. Pediatrics international : official journal of the Japan Pediatric Society. 2013;55(2):e32-4.

103. Jap TS, Wu YC, Chiou JY, Kwok CF. A novel mutation in the hepatocyte nuclear factor-1alpha/MODY3 gene in Chinese subjects with early-onset Type 2 diabetes mellitus in Taiwan. Diabetic medicine : a journal of the British Diabetic Association. 2000;17(5):390-3.

104. Johansson BB, Irgens HU, Molnes J, Sztromwasser P, Aukrust I, Juliusson PB, et al. Targeted next-generation sequencing reveals MODY in up to 6.5% of antibody-negative diabetes cases listed in the Norwegian Childhood Diabetes Registry. Diabetologia. 2017;60(4):625-35.

105. Sagen JV, Bjorkhaug L, Haukanes BI, Grevle L, Molnes J, Nedrebo BG, et al. The HNF1A mutant Ala180Val: Clinical challenges in determining causality of a rare HNF1A variant in familial diabetes. Diabetes research and clinical practice. 2017;133:142-9.

106. Iwen KA, Klein J, Hubold C, Lehnert H, Weitzel JM. Maturity-onset diabetes of the young and hepatic adenomatosis - characterisation of a new mutation. Experimental and clinical endocrinology & diabetes : official journal, German Society of Endocrinology [and] German Diabetes Association. 2013;121(6):368-71.

107. Bjorkhaug L, Sagen JV, Thorsby P, Sovik O, Molven A, Njolstad PR. Hepatocyte nuclear factor-1 alpha gene mutations and diabetes in Norway. The Journal of clinical endocrinology and metabolism. 2003;88(2):920-31.

108. Lopez-Garrido MP, Herranz-Antolin S, Alija-Merillas MJ, Giralt P, Escribano J. Co-inheritance of HNF1a and GCK mutations in a family with maturity-onset diabetes of the young (MODY): implications for genetic testing. Clinical endocrinology. 2013;79(3):342-7.

109. Jesic MD, Sajic S, Jesic MM, Maringa M, Micic D, Necic S. A case of new mutation in maturity-onset diabetes of the young type 3 (MODY 3) responsive to a low dose of sulphonylurea. Diabetes research and clinical practice. 2008;81(1):e1-3.

110. Moritani M, Yokota I, Horikawa R, Urakami T, Nishii A, Kawamura T, et al. Identification of monogenic gene mutations in Japanese subjects diagnosed with type 1B diabetes between >5 and 15.1 years of age. Journal of pediatric endocrinology & metabolism : JPEM. 2016;29(9):1047-54.

111. Kyithar MP, Bacon S, Pannu KK, Rizvi SR, Colclough K, Ellard S, et al. Identification of HNF1A-MODY and HNF4A-MODY in Irish families: phenotypic characteristics and therapeutic implications. Diabetes & metabolism. 2011;37(6):512-9.

112. Frayling TM, Bulamn MP, Ellard S, Appleton M, Dronsfield MJ, Mackie AD, et al. Mutations in the hepatocyte nuclear factor-1alpha gene are a common cause of maturity-onset diabetes of the young in the U.K. Diabetes. 1997;46(4):720-5.

113. Kitanaka S, Miki Y, Hayashi Y, Igarashi T. Promoter-specific repression of hepatocyte nuclear factor (HNF)-1 beta and HNF-1 alpha transcriptional activity by an HNF-1 beta missense mutant associated with Type 5 maturity-onset diabetes of the young with hepatic and biliary manifestations. The Journal of clinical endocrinology and metabolism. 2004;89(3):1369-78.

114. Yamada S, Nishigori H, Onda H, Utsugi T, Yanagawa T, Maruyama T, et al. Identification of mutations in the hepatocyte nuclear factor (HNF)-1 alpha gene in Japanese subjects with IDDM. Diabetes. 1997;46(10):1643-7.

115. Kwak SH, Jung CH, Ahn CH, Park J, Chae J, Jung HS, et al. Clinical whole exome sequencing in early onset diabetes patients. Diabetes research and clinical practice. 2016;122:71-7.

116. Khelifa SB, Dendana A, Barboura I, Khochtali I, Chahed H, Ferchichi S, et al. Successful switch from insulin to oral sulfonylurea therapy in HNF1A-MODY Tunisian patient with the P291fsinsC mutation. Diabetes research and clinical practice. 2016;115:133-6.

117. Cantu S, Krier J, Hashemi N. Hepatocyte Nuclear Factor 1alpha Mutation-associated MODY-3 and Familial Liver Adenomatosis. Journal of clinical gastroenterology. 2016;50(2):181-2.

118. Elbein SC, Teng K, Yount P, Scroggin E. Linkage and molecular scanning analyses of MODY3/hepatocyte nuclear factor-1 alpha gene in typical familial type 2 diabetes: evidence for novel mutations in exons 8 and 10. The Journal of clinical endocrinology and metabolism. 1998;83(6):2059-65.

119. Awata T, Kurihara S, Inoue K, Inoue I, Takei S, Ishii C, et al. A novel missense mutation in the homeodomain of the hepatocyte nuclear factor-1alpha/maturity-onset diabetes of the young 3 in a Japanese early-onset type 2 diabetic patient and time-course of glucose-stimulated insulin secretion. Diabetes care. 1998;21(9):1569-71.

120. Tatsi C, Kanaka-Gantenbein C, Vazeou-Gerassimidi A, Chrysis D, Delis D, Tentolouris N, et al. The spectrum of HNF1A gene mutations in Greek patients with MODY3: relative frequency and identification of seven novel germline mutations. Pediatric diabetes. 2013;14(7):526-34.

121. Hansen T, Eiberg H, Rouard M, Vaxillaire M, Moller AM, Rasmussen SK, et al. Novel MODY3 mutations in the hepatocyte nuclear factor-1alpha gene: evidence for a hyperexcitability of pancreatic beta-cells to intravenous secretagogues in a glucose-tolerant carrier of a P447L mutation. Diabetes. 1997;46(4):726-30.

122. Radha V, Ek J, Anuradha S, Hansen T, Pedersen O, Mohan V. Identification of novel variants in the hepatocyte nuclear factor-1alpha gene in South Indian patients with maturity onset diabetes of young. The Journal of clinical endocrinology and metabolism. 2009;94(6):1959-65.

123. Wang X, Wang T, Yu M, Zhang H, Ping F, Zhang Q, et al. Screening of HNF1A and HNF4A mutation and clinical phenotype analysis in a large cohort of Chinese patients with maturity-onset diabetes of the young. Acta diabetologica. 2019;56(3):281-8.

124. Yang Z, Wu SH, Zheng TS, Lu HJ, Xiang KS. Identification of four novel mutations in the HNF-1A gene in Chinese early-onset and/or multiplex diabetes pedigrees. Chinese medical journal. 2006;119(13):1072-8.

125. Zubkova N, Burumkulova F, Plechanova M, Petrukhin V, Petrov V, Vasilyev E, et al. High frequency of pathogenic and rare sequence variants in diabetes-related genes among Russian patients with diabetes in pregnancy. Acta diabetologica. 2019;56(4):413-20.

126. Ovsyannikova AK, Rymar OD, Ivanoshchuk DE, Mikhailova SV, Shakhtshneider EV, Orlov PS, et al. A Case of Maturity Onset Diabetes of the Young (MODY3) in a Family with a Novel HNF1A Gene Mutation in Five Generations. Diabetes therapy : research, treatment and education of diabetes and related disorders. 2018;9(1):413-20.

127. Bellanne-Chantelot C, Carette C, Riveline JP, Valero R, Gautier JF, Larger E, et al. The type and the position of HNF1A mutation modulate age at diagnosis of diabetes in patients with maturity-onset diabetes of the young (MODY)-3. Diabetes. 2008;57(2):503-8.

128. Bonatto N, Nogaroto V, Svidnicki PV, Milleo FQ, Grassiolli S, Almeida MC, et al. Variants of the HNF1alpha gene: A molecular approach concerning diabetic patients from southern Brazil. Genetics and molecular biology. 2012;35(4):737-40.

129. Galan M, Garcia-Herrero CM, Azriel S, Gargallo M, Duran M, Gorgojo JJ, et al. Differential effects of HNF-1alpha mutations associated with familial young-onset diabetes on target gene regulation. Mol Med. 2011;17(3-4):256-65.

130. Glotov OS, Serebryakova EA, Turkunova ME, Efimova OA, Glotov AS, Barbitoff YA, et al. Wholeexome sequencing in Russian children with nontype 1 diabetes mellitus reveals a wide spectrum of genetic variants in MODYrelated and unrelated genes. Molecular medicine reports. 2019;20(6):4905-14.

131. Habeb AM, George ET, Mathew V, Hattersley AL. Response to oral gliclazide in a pre-pubertal child with hepatic nuclear factor-1 alpha maturity onset diabetes of the young. Annals of Saudi medicine. 2011;31(2):190-3.

132. Knebel B, Mack S, Haas J, Herman-Friede MK, Lange S, Schubert O, et al. Divergent phenotypes in siblings with identical novel mutations in the HNF-1alpha gene leading to maturity onset diabetes of the young type 3. BMC medical genetics. 2016;17(1):36.

133. Moghbeli M, Naghibzadeh B, Ghahraman M, Fatemi S, Taghavi M, Vakili R, et al. Mutations in HNF1A Gene are not a Common Cause of Familial Young-Onset Diabetes in Iran. Indian journal of clinical biochemistry : IJCB. 2018;33(1):91-5.

134. Nogaroto V, Svidnicki PV, Bonatto N, Milleo FQ, Almeida MC, Vicari MR, et al. New HNF-1alpha nonsense mutation causes maturity-onset diabetes of the young type 3. Clinics (Sao Paulo). 2011;66(1):167-8.

135. Pace NP, Rizzo C, Abela A, Gruppetta M, Fava S, Felice A, et al. Identification of an HNF1A p.Gly292fs Frameshift Mutation Presenting as Diabetes During Pregnancy in a Maltese Family. Clinical medicine insights Case reports. 2019;12:1179547619831034.

136. Pavic T, Juszczak A, Pape Medvidovic E, Burrows C, Sekerija M, Bennett AJ, et al. Maturity onset diabetes of the young due to HNF1A variants in Croatia. Biochemia medica. 2018;28(2):020703.

137. Plengvidhya N, Tangjittipokin W, Teerawattanapong N, Narkdontri T, Yenchitsomanus PT. HNF1A mutation in a Thai patient with maturity-onset diabetes of the young: A case report. World journal of diabetes. 2019;10(7):414-20.

138. Rama Chandran S, Bhalshankar J, Farhad Vasanwala R, Zhao Y, Owen KR, Su-Lyn Gardner D. Traditional clinical criteria outperform high-sensitivity C-reactive protein for the screening of hepatic nuclear factor 1 alpha maturity-onset diabetes of the young among young Asians with diabetes. Therapeutic advances in endocrinology and metabolism. 2018;9(9):271-82.

139. Shankar RK, Ellard S, Standiford D, Pihoker C, Gilliam LK, Hattersley A, et al. Digenic heterozygous HNF1A and HNF4A mutations in two siblings with childhood-onset diabetes. Pediatric diabetes. 2013;14(7):535-8.

140. Shields BM, Shepherd M, Hudson M, McDonald TJ, Colclough K, Peters J, et al. Population-Based Assessment of a Biomarker-Based Screening Pathway to Aid Diagnosis of Monogenic Diabetes in Young-Onset Patients. Diabetes care. 2017;40(8):1017-25.

141. Tang J, Tang CY, Wang F, Guo Y, Tang HN, Zhou CL, et al. Genetic diagnosis and treatment of a Chinese ketosis-prone MODY 3 family with depression. Diabetology & metabolic syndrome. 2017;9:5.

142. Urrutia I, Martinez R, Rica I, Martinez de LaPiscina I, Garcia-Castano A, Aguayo A, et al. Negative autoimmunity in a Spanish pediatric cohort suspected of type 1 diabetes, could it be monogenic diabetes? PloS one. 2019;14(7):e0220634.

143. Xu JY, Dan QH, Chan V, Wat NM, Tam S, Tiu SC, et al. Genetic and clinical characteristics of maturity-onset diabetes of the young in Chinese patients. European journal of human genetics : EJHG. 2005;13(4):422-7.

144. Mohammadi A, Eskandari A, Sarmadi A, Rahimi M, Iraj B, Hashemipour M, et al. Genetic Study of Hepatocyte Nuclear Factor 1 Alpha Variants in Development of Early-Onset Diabetes Type 2 and Maturity-Onset Diabetes of the Young 3 in Iran. Advanced biomedical research. 2019;8:55.

145. Moller AM, Dalgaard LT, Ambye L, Hansen L, Schmitz O, Hansen T, et al. A novel Phe75fsdelT mutation in the hepatocyte nuclear factor-4alpha gene in a Danish pedigree with maturity-onset diabetes of the young. The Journal of clinical endocrinology and metabolism. 1999;84(1):367-9.

146. Anuradha S, Radha V, Mohan V. Association of novel variants in the hepatocyte nuclear factor 4A gene with maturity onset diabetes of the young and early onset type 2 diabetes. Clinical genetics. 2011;80(6):541-9.

147. Bulman MP, Dronsfield MJ, Frayling T, Appleton M, Bain SC, Ellard S, et al. A missense mutation in the hepatocyte nuclear factor 4 alpha gene in a UK pedigree with maturity-onset diabetes of the young. Diabetologia. 1997;40(7):859-62.

148. Gragnoli C, Lindner T, Cockburn BN, Kaisaki PJ, Gragnoli F, Marozzi G, et al. Maturity-onset diabetes of the young due to a mutation in the hepatocyte nuclear factor-4 alpha binding site in the promoter of the hepatocyte nuclear factor-1 alpha gene. Diabetes. 1997;46(10):1648-51.

149. Furuta H, Iwasaki N, Oda N, Hinokio Y, Horikawa Y, Yamagata K, et al. Organization and partial sequence of the hepatocyte nuclear factor-4 alpha/MODY1 gene and identification of a missense mutation, R127W, in a Japanese family with MODY. Diabetes. 1997;46(10):1652-7.

150. Apperley L, Giri D, Houghton JAL, Flanagan SE, Didi M, Senniappan S. A rare case of congenital hyperinsulinism (CHI) due to dual genetic aetiology involving HNF4A and ABCC8. Journal of pediatric endocrinology & metabolism : JPEM. 2019;32(3):301-4.

151. Sanyoura M, Letourneau L, Knight Johnson AE, Del Gaudio D, Greeley SAW, Philipson LH, et al. GCK-MODY in the US Monogenic Diabetes Registry: Description of 27 unpublished variants. Diabetes research and clinical practice. 2019;151:231-6.

152. Ushijima K, Fukami M, Ayabe T, Narumi S, Okuno M, Nakamura A, et al. Comprehensive screening for monogenic diabetes in 89 Japanese children with insulin-requiring antibody-negative type 1 diabetes. Pediatric diabetes. 2018;19(2):243-50.

153. Arya VB, Rahman S, Senniappan S, Flanagan SE, Ellard S, Hussain K. HNF4A mutation: switch from hyperinsulinaemic hypoglycaemia to maturity-onset diabetes of the young, and incretin response. Diabetic medicine : a journal of the British Diabetic Association. 2014;31(3):e11-5.

154. Forlani G, Zucchini S, Di Rocco A, Di Luzio R, Scipione M, Marasco E, et al. Double heterozygous mutations involving both HNF1A/MODY3 and HNF4A/MODY1 genes: a case report. Diabetes care. 2010;33(11):2336-8.

155. Hani EH, Suaud L, Boutin P, Chevre JC, Durand E, Philippi A, et al. A missense mutation in hepatocyte nuclear factor-4 alpha, resulting in a reduced transactivation activity, in human late-onset non-insulin-dependent diabetes mellitus. The Journal of clinical investigation. 1998;101(3):521-6.

156. Hara K, Noda M, Waki H, Tobe K, Yamauchi T, Kadowaki H, et al. Maturity-onset diabetes of the young resulting from a novel mutation in the HNF-4alpha gene. Intern Med. 2002;41(10):848-52.

157. Johansson S, Irgens H, Chudasama KK, Molnes J, Aerts J, Roque FS, et al. Exome sequencing and genetic testing for MODY. PloS one. 2012;7(5):e38050.

158. Gordon K, Yao M, Siegel R, Stackpole K. A Case of a 13-Year-Old Female With Maturity Onset Diabetes of the Young (MODY) Identified by School-Based Cardiovascular Screening. Global pediatric health. 2019;6:2333794X19874215.

159. Laver TW, Colclough K, Shepherd M, Patel K, Houghton JA, Dusatkova P, et al. The Common p.R114W HNF4A Mutation Causes a Distinct Clinical Subtype of Monogenic Diabetes. Diabetes. 2016;65(10):3212-7.

160. Lindner T, Gragnoli C, Furuta H, Cockburn BN, Petzold C, Rietzsch H, et al. Hepatic function in a family with a nonsense mutation (R154X) in the hepatocyte nuclear factor-4alpha/MODY1 gene. The Journal of clinical investigation. 1997;100(6):1400-5.

161. Delvecchio M, Di Paola R, Mangiacotti D, Sacco M, Menzaghi C, Trischitta V. Clinical heterogeneity of abnormal glucose homeostasis associated with the HNF4A R311H mutation. Italian journal of pediatrics. 2014;40:58.

162. Raeder H, Bjorkhaug L, Johansson S, Mangseth K, Sagen JV, Hunting A, et al. A hepatocyte nuclear factor-4 alpha gene (HNF4A) P2 promoter haplotype linked with late-onset diabetes: studies of HNF4A variants in the Norwegian MODY registry. Diabetes. 2006;55(6):1899-903.

163. Taghavi SM, Fatemi SS, Rafatpanah H, Ganjali R, Tavakolafshari J, Valizadeh N. Mutations in the coding regions of the hepatocyte nuclear factor 4 alpha in Iranian families with maturity onset diabetes of the young. Cardiovascular diabetology. 2009;8:63.

164. Bellanne-Chantelot C, Chauveau D, Gautier JF, Dubois-Laforgue D, Clauin S, Beaufils S, et al. Clinical spectrum associated with hepatocyte nuclear factor-1beta mutations. Annals of internal medicine. 2004;140(7):510-7.

165. Mayer C, Bottcher Y, Kovacs P, Halbritter J, Stumvoll M. Phenotype of a patient with a de novo mutation in the hepatocyte nuclear factor 1beta/maturity-onset diabetes of the young type 5 gene. Metabolism: clinical and experimental. 2008;57(3):416-20.

166. Shimoda Y, Okada S, Shimoyama Y, Kusano M, Yamada M. Maturity onset diabetes of the young 5 accompanied by duodenal cysts. Journal of diabetes. 2016;8(3):448-9.

167. Thirumalai A, Holing E, Brown Z, Gilliam LK. A case of hepatocyte nuclear factor-1beta (TCF2) maturity onset diabetes of the young misdiagnosed as type 1 diabetes and treated unnecessarily with insulin. Journal of diabetes. 2013;5(4):462-4.

168. Wentworth JM, Lukic V, Bahlo M, Finlay M, Nguyen C, Morahan G, et al. Maturity-onset diabetes of the young type 5 in a family with diabetes and mild kidney disease diagnosed by whole exome sequencing. Internal medicine journal. 2014;44(11):1137-40.

169. Wang Y, Zhao Y, Zhang J, Yang Y, Liu F. A case of a novel mutation in HNF1beta-related maturity-onset diabetes of the young type 5 with diabetic kidney disease complication in a Chinese family. Journal of diabetes and its complications. 2017;31(7):1243-6.

170. Luo Y, Dai Z, Li L, Shan X, Wu C. Hepatocyte nuclear factor 1beta maturity-onset diabetes of the young in a Chinese child presenting with hyperglycemic hyperosmolar state. Acta diabetologica. 2017;54(10):969-73.

171. Bellanne-Chantelot C, Clauin S, Chauveau D, Collin P, Daumont M, Douillard C, et al. Large genomic rearrangements in the hepatocyte nuclear factor-1beta (TCF2) gene are the most frequent cause of maturity-onset diabetes of the young type 5. Diabetes. 2005;54(11):3126-32.

172. Carrillo E, Lomas A, Pines PJ, Lamas C. Long-lasting response to oral therapy in a young male with monogenic diabetes as part of HNF1B-related disease. Endocrinology, diabetes & metabolism case reports. 2017;2017.

173. Fujimoto K, Sasaki T, Hiki Y, Nemoto M, Utsunomiya Y, Yokoo T, et al. In vitro and pathological investigations of MODY5 with the R276X-HNF1beta (TCF2) mutation. Endocrine journal. 2007;54(5):757-64.

174. Jo W, Sano H, Sudo A, Matsunami Y, Kawamura N, Tajima T. A Case of Novel Mutation of HNF1B in Maturity-onset Diabetes of the Young Type 5 (MODY5). Clinical pediatric endocrinology : case reports and clinical investigations : official journal of the Japanese Society for Pediatric Endocrinology. 2012;21(3):53-5.

175. Kato T, Tanaka D, Muro S, Jambaljav B, Mori E, Yonemitsu S, et al. A Novel p.L145Q Mutation in the HNF1B Gene in a Case of Maturity-onset Diabetes of the Young Type 5 (MODY5). Intern Med. 2018;57(14):2035-9.

176. Kim EK, Lee JS, Cheong HI, Chung SS, Kwak SH, Park KS. Identification and Functional Characterization of P159L Mutation in HNF1B in a Family with Maturity-Onset Diabetes of the Young 5 (MODY5). Genomics & informatics. 2014;12(4):240-6.

177. Pace NP, Craus J, Felice A, Vassallo J. Case Report: Identification of an HNF1B p.Arg527Gln mutation in a Maltese patient with atypical early onset diabetes and diabetic nephropathy. BMC endocrine disorders. 2018;18(1):28.

178. Zhou P, Wei R, Guo Z, Zhu H, Campbell D, Li Q, et al. A Single Nucleotide Variant in HNF-1beta is Associated with Maturity-Onset Diabetes of the Young in a Large Chinese Family. Iranian journal of public health. 2016;45(2):170-8.

179. Piccini B, Artuso R, Lenzi L, Guasti M, Braccesi G, Barni F, et al. Clinical and molecular characterization of a novel INS mutation identified in patients with MODY phenotype. European journal of medical genetics. 2016;59(11):590-5.

180. Yan J, Jiang F, Zhang R, Xu T, Zhou Z, Ren W, et al. Whole-exome sequencing identifies a novel INS mutation causative of maturity-onset diabetes of the young 10. Journal of molecular cell biology. 2017;9(5):376-83.

181. Johnson SR, McGown I, Oppermann U, Conwell LS, Harris M, Duncan EL. A novel INS mutation in a family with maturity-onset diabetes of the young: Variable insulin secretion and putative mechanisms. Pediatric diabetes. 2018;19(5):905-9.

182. Dusatkova L, Dusatkova P, Vosahlo J, Vesela K, Cinek O, Lebl J, et al. Frameshift mutations in the insulin gene leading to prolonged molecule of insulin in two families with Maturity-Onset Diabetes of the Young. European journal of medical genetics. 2015;58(4):230-4.

183. Xiao X, Liu L, Xiao Y, Xie Z, Li L, Zhou H, et al. Novel frameshift mutation in the insulin (INS) gene in a family with maturity onset diabetes of the young (MODY). Journal of diabetes. 2019;11(1):83-6.

184. Boesgaard TW, Pruhova S, Andersson EA, Cinek O, Obermannova B, Lauenborg J, et al. Further evidence that mutations in INS can be a rare cause of Maturity-Onset Diabetes of the Young (MODY). BMC medical genetics. 2010;11:42.

185. Molven A, Ringdal M, Nordbo AM, Raeder H, Stoy J, Lipkind GM, et al. Mutations in the insulin gene can cause MODY and autoantibody-negative type 1 diabetes. Diabetes. 2008;57(4):1131-5.

186. Cattoni A, Jackson C, Bain M, Houghton J, Wei C. Phenotypic variability in two siblings with monogenic diabetes due to the same ABCC8 gene mutation. Pediatric diabetes. 2019;20(4):482-5.

187. Dallali H, Pezzilli S, Hechmi M, Sallem OK, Elouej S, Jmel H, et al. Genetic characterization of suspected MODY patients in Tunisia by targeted next-generation sequencing. Acta diabetologica. 2019;56(5):515-23.

188. Johnson SR, Leo P, Conwell LS, Harris M, Brown MA, Duncan EL. Clinical usefulness of comprehensive genetic screening in maturity onset diabetes of the young (MODY): A novel ABCC8 mutation in a previously screened family. Journal of diabetes. 2018;10(9):764-7.

189. Koufakis T, Sertedaki A, Tatsi EB, Trakatelli CM, Karras SN, Manthou E, et al. First Report of Diabetes Phenotype due to a Loss-of-Function ABCC8 Mutation Previously Known to Cause Congenital Hyperinsulinism. Case reports in genetics. 2019;2019:3654618.

190. Shima KR, Usuda R, Futatani T, Akahori H, Kaneko S, Yorifuji T, et al. Heterogeneous nature of diabetes in a family with a gain-of-function mutation in the ATP-binding cassette subfamily C member 8 (ABCC8) gene. Endocrine journal. 2018;65(10):1055-9.

191. Caetano LA, Santana LS, Costa-Riquetto AD, Lerario AM, Nery M, Nogueira GF, et al. PDX1 -MODY and dorsal pancreatic agenesis: New phenotype of a rare disease. Clinical genetics. 2018;93(2):382-6.

192. Deng M, Xiao X, Zhou L, Wang T. First Case Report of Maturity-Onset Diabetes of the Young Type 4 Pedigree in a Chinese Family. Frontiers in endocrinology. 2019;10:406.

193. Doddabelavangala Mruthyunjaya M, Chapla A, Hesarghatta Shyamasunder A, Varghese D, Varshney M, Paul J, et al. Comprehensive Maturity Onset Diabetes of the Young (MODY) Gene Screening in Pregnant Women with Diabetes in India. PloS one. 2017;12(1):e0168656.

194. Mangrum C, Rush E, Shivaswamy V. Genetically Targeted Dipeptidyl Peptidase-4 Inhibitor Use in a Patient with a Novel Mutation of MODY type 4. Clinical medicine insights Endocrinology and diabetes. 2015;8:83-6.

195. Horikawa Y, Enya M, Mabe H, Fukushima K, Takubo N, Ohashi M, et al. NEUROD1-deficient diabetes (MODY6): Identification of the first cases in Japanese and the clinical features. Pediatric diabetes. 2018;19(2):236-42.

196. Szopa M, Ludwig-Galezowska AH, Radkowski P, Skupien J, Machlowska J, Klupa T, et al. A family with the Arg103Pro mutation in the NEUROD1 gene detected by next-generation sequencing - Clinical characteristics of mutation carriers. European journal of medical genetics. 2016;59(2):75-9.

197. Abreu GM, Tarantino RM, Cabello PH, Zembrzuski VM, da Fonseca ACP, Rodacki M, et al. The first case of NEUROD1-MODY reported in Latin America. Molecular genetics & genomic medicine. 2019;7(12):e989.

198. Sujjitjoon J, Kooptiwut S, Chongjaroen N, Tangjittipokin W, Plengvidhya N, Yenchitsomanus PT. Aberrant mRNA splicing of paired box 4 (PAX4) IVS7-1G>A mutation causing maturity-onset diabetes of the young, type 9. Acta diabetologica. 2016;53(2):205-16.

199. Plengvidhya N, Kooptiwut S, Songtawee N, Doi A, Furuta H, Nishi M, et al. PAX4 mutations in Thais with maturity onset diabetes of the young. The Journal of clinical endocrinology and metabolism. 2007;92(7):2821-6.

200. Jo W, Endo M, Ishizu K, Nakamura A, Tajima T. A novel PAX4 mutation in a Japanese patient with maturity-onset diabetes of the young. The Tohoku journal of experimental medicine. 2011;223(2):113-8.

201. Pezzilli S, Ludovico O, Biagini T, Mercuri L, Alberico F, Lauricella E, et al. Insights From Molecular Characterization of Adult Patients of Families With Multigenerational Diabetes. Diabetes. 2018;67(1):137-45.

202. Borowiec M, Liew CW, Thompson R, Boonyasrisawat W, Hu J, Mlynarski WM, et al. Mutations at the BLK locus linked to maturity onset diabetes of the young and beta-cell dysfunction. Proceedings of the National Academy of Sciences of the United States of America. 2009;106(34):14460-5.

203. Johnson SR, Ellis JJ, Leo PJ, Anderson LK, Ganti U, Harris JE, et al. Comprehensive genetic screening: The prevalence of maturity-onset diabetes of the young gene variants in a population-based childhood diabetes cohort. Pediatric diabetes. 2019;20(1):57-64.

204. Ushijima K, Narumi S, Ogata T, Yokota I, Sugihara S, Kaname T, et al. KLF11 variant in a family clinically diagnosed with early childhood-onset type 1B diabetes. Pediatric diabetes. 2019;20(6):712-9.

205. Prudente S, Jungtrakoon P, Marucci A, Ludovico O, Buranasupkajorn P, Mazza T, et al. Loss-of-Function Mutations in APPL1 in Familial Diabetes Mellitus. American journal of human genetics. 2015;97(1):177-85.

206. Carmody D, Park SY, Ye H, Perrone ME, Alkorta-Aranburu G, Highland HM, et al. Continued lessons from the INS gene: an intronic mutation causing diabetes through a novel mechanism. Journal of medical genetics. 2015;52(9):612-6.

207. Dotto RP, Santana LS, Lindsey SC, Caetano LA, Franco LF, Moises R, et al. Searching for mutations in the HNF1B gene in a Brazilian cohort with renal cysts and hyperglycemia. Archives of endocrinology and metabolism. 2019;63(3):250-7.

208. Cho EH, Min JW, Choi SS, Choi HS, Kim SW. Identification of Maturity-Onset Diabetes of the Young Caused by Glucokinase Mutations Detected Using Whole-Exome Sequencing. Endocrinol Metab (Seoul). 2017;32(2):296-301.

209. Franco LF, Peixoto-Barbosa R, Dotto RP, Vieira JGH, Dias-da-Silva MR, Reis LCF, et al. More than kin, less than kind: one family and the many faces of diabetes in youth. Archives of endocrinology and metabolism. 2017;61(6):637-42.

210. Haring MPD, Vriesendorp TM, Klein Wassink-Ruiter JS, de Haas RJ, Gouw ASH, de Meijer VE. Diagnosis of hepatocellular adenoma in men before onset of diabetes in HNF1A-MODY: Watch out for winkers. Liver international : official journal of the International Association for the Study of the Liver. 2019;39(11):2042-5.

211. Gjesing AP, Rui G, Lauenborg J, Have CT, Hollensted M, Andersson E, et al. High Prevalence of Diabetes-Predisposing Variants in MODY Genes Among Danish Women With Gestational Diabetes Mellitus. Journal of the Endocrine Society. 2017;1(6):681-90.

212. Kleinberger JW, Copeland KC, Gandica RG, Haymond MW, Levitsky LL, Linder B, et al. Monogenic diabetes in overweight and obese youth diagnosed with type 2 diabetes: the TODAY clinical trial. Genetics in medicine : official journal of the American College of Medical Genetics. 2018;20(6):583-90.

213. Kapoor RR, Locke J, Colclough K, Wales J, Conn JJ, Hattersley AT, et al. Persistent hyperinsulinemic hypoglycemia and maturity-onset diabetes of the young due to heterozygous HNF4A mutations. Diabetes. 2008;57(6):1659-63.

214. Mohan V, Radha V, Nguyen TT, Stawiski EW, Pahuja KB, Goldstein LD, et al. Comprehensive genomic analysis identifies pathogenic variants in maturity-onset diabetes of the young (MODY) patients in South India. BMC medical genetics. 2018;19(1):22.

215. Ovsyannikova AK, Rymar OD, Shakhtshneider EV, Klimontov VV, Koroleva EA, Myakina NE, et al. ABCC8-Related Maturity-Onset Diabetes of the Young (MODY12): Clinical Features and Treatment Perspective. Diabetes therapy : research, treatment and education of diabetes and related disorders. 2016;7(3):591-600.

216. Poitou C, Francois H, Bellanne-Chantelot C, Noel C, Jacquet A, Clauin S, et al. Maturity onset diabetes of the young: clinical characteristics and outcome after kidney and pancreas transplantation in MODY3 and RCAD patients: a single center experience. Transplant international : official journal of the European Society for Organ Transplantation. 2012;25(5):564-72.

217. Yamagata K, Oda N, Kaisaki PJ, Menzel S, Furuta H, Vaxillaire M, et al. Mutations in the hepatocyte nuclear factor-1alpha gene in maturity-onset diabetes of the young (MODY3). Nature. 1996;384(6608):455-8.

218. Fendler W, Malachowska B, Baranowska-Jazwiecka A, Borowiec M, Wyka K, Malecki MT, et al. Population-based estimates for double diabetes amongst people with glucokinase monogenic diabetes, GCK-MODY. Diabetic medicine : a journal of the British Diabetic Association. 2014;31(7):881-3.

219. Huopio H, Miettinen PJ, Ilonen J, Nykanen P, Veijola R, Keskinen P, et al. Clinical, Genetic, and Biochemical Characteristics of Early-Onset Diabetes in the Finnish Population. The Journal of clinical endocrinology and metabolism. 2016;101(8):3018-26.

220. Yaghootkar H, Abbasi F, Ghaemi N, Rabbani A, Wakeling MN, Eshraghi P, et al. Type 1 diabetes genetic risk score discriminates between monogenic and Type 1 diabetes in children diagnosed at the age of <5 years in the Iranian population. Diabetic medicine : a journal of the British Diabetic Association. 2019;36(12):1694-702.

221. Yellapu NK, Valasani KR, Pasupuleti SK, Gopal S, Potukuchi Venkata Gurunadha Krishna S, Matcha B. Identification and analysis of novel R308K mutation in glucokinase of type 2 diabetic patient and its kinetic correlation. Biotechnology and applied biochemistry. 2014;61(5):572-81.

222. Yorifuji T, Fujimaru R, Hosokawa Y, Tamagawa N, Shiozaki M, Aizu K, et al. Comprehensive molecular analysis of Japanese patients with pediatric-onset MODY-type diabetes mellitus. Pediatric diabetes. 2012;13(1):26-32.

223. Yorifuji T, Higuchi S, Kawakita R, Hosokawa Y, Aoyama T, Murakami A, et al. Genetic basis of early-onset, maturity-onset diabetes of the young-like diabetes in Japan and features of patients without mutations in the major MODY genes: Dominance of maternal inheritance. Pediatric diabetes. 2018;19(7):1164-72.

224. Sahoo SK, Zaidi G, Vipin VP, Chapla A, Thomas N, Yu L, et al. Heterogeneity in the aetiology of diabetes mellitus in young adults: A prospective study from north India. The Indian journal of medical research. 2019;149(4):479-88.

225. Froguel P, Zouali H, Vionnet N, Velho G, Vaxillaire M, Sun F, et al. Familial hyperglycemia due to mutations in glucokinase. Definition of a subtype of diabetes mellitus. The New England journal of medicine. 1993;328(10):697-702.

226. Mikuscheva A, McKenzie E, Mekhail A. 21-Year-Old Pregnant Woman with MODY-5 Diabetes. Case reports in obstetrics and gynecology. 2017;2017:6431531.

227. Li HJ, Groden C, Hoenig MP, Ray EC, Ferreira CR, Gahl W, et al. Case report: extreme coronary calcifications and hypomagnesemia in a patient with a 17q12 deletion involving HNF1B. BMC nephrology. 2019;20(1):353.

228. Marucci A, Biagini T, Di Paola R, Menzaghi C, Fini G, Castellana S, et al. Association of a homozygous GCK missense mutation with mild diabetes. Molecular genetics & genomic medicine. 2019;7(7):e00728.

229. Omura Y, Yagi K, Honoki H, Iwata M, Enkaku A, Takikawa A, et al. Clinical manifestations of a sporadic maturity-onset diabetes of the young (MODY) 5 with a whole deletion of HNF1B based on 17q12 microdeletion. Endocrine journal. 2019;66(12):1113-6.

230. Stiles CE, Thuraisingham R, Bockenhauer D, Platts L, Kumar AV, Korbonits M. De novo HNF1 homeobox B mutation as a cause for chronic, treatment-resistant hypomagnesaemia. Endocrinology, diabetes & metabolism case reports. 2018;2018.

231. Clocquet AR, Egan JM, Stoffers DA, Muller DC, Wideman L, Chin GA, et al. Impaired insulin secretion and increased insulin sensitivity in familial maturity-onset diabetes of the young 4 (insulin promoter factor 1 gene). Diabetes. 2000;49(11):1856-64.

232. Edghill EL, Stals K, Oram RA, Shepherd MH, Hattersley AT, Ellard S. HNF1B deletions in patients with young-onset diabetes but no known renal disease. Diabetic medicine : a journal of the British Diabetic Association. 2013;30(1):114-7.

233. Ellard S, Thomas K, Edghill EL, Owens M, Ambye L, Cropper J, et al. Partial and whole gene deletion mutations of the GCK and HNF1A genes in maturity-onset diabetes of the young. Diabetologia. 2007;50(11):2313-7.

234. Raile K, Klopocki E, Holder M, Wessel T, Galler A, Deiss D, et al. Expanded clinical spectrum in hepatocyte nuclear factor 1b-maturity-onset diabetes of the young. The Journal of clinical endocrinology and metabolism. 2009;94(7):2658-64.

235. Roehlen N, Hilger H, Stock F, Glaser B, Guhl J, Schmitt-Graeff A, et al. 17q12 Deletion Syndrome as a Rare Cause for Diabetes Mellitus Type MODY5. The Journal of clinical endocrinology and metabolism. 2018;103(10):3601-10.

236. Taberner P, Flanagan SE, Mackay DJ, Ellard S, Taverna MJ, Ferraro M. Clinical and genetic features of Argentinian children with diabetes-onset before 12months of age: Successful transfer from insulin to oral sulfonylurea. Diabetes research and clinical practice. 2016;117:104-10.

237. Willson JS, Godwin TD, Wiggins GA, Guilford PJ, McCall JL. Primary hepatocellular neoplasms in a MODY3 family with a novel HNF1A germline mutation. Journal of hepatology. 2013;59(4):904-7.

238. Li M, Wang S, Xu K, Chen Y, Fu Q, Gu Y, et al. High Prevalence of a Monogenic Cause in Han Chinese Diagnosed With Type 1 Diabetes, Partly Driven by Nonsyndromic Recessive WFS1 Mutations. Diabetes. 2020;69(1):121-6.

239. Dominguez-Lopez A, Miliar-Garcia A, Segura-Kato YX, Riba L, Esparza-Lopez R, Ramirez-Jimenez S, et al. Mutations in MODY genes are not common cause of early-onset type 2 diabetes in Mexican families. JOP : Journal of the pancreas. 2005;6(3):238-45.
