# Supplementary material for: Causal variants in Maturity Onset Diabetes of the Young (MODY) – A systematic review
Source: BMC Endocr Disord. 2021 Nov 11;21:223. doi: 10.1186/s12902-021-00891-7 (PMC8582101; doi:10.1186/s12902-021-00891-7)
Supplement: Supplementary file 3 — Appendix C: Supplementary Table 3. [file 12902_2021_891_MOESM3_ESM.docx]

**Supplementary table 3: Mutations retrieved from Clinvar that could not be identified in literature review:**

| **S.No** | **DNA changes** | **Protein change** | **Accession number** | **Gene** | **Interpretation** |
| --- | --- | --- | --- | --- | --- |
|  | c.1253+2T>A |  | VCV000393449 | GCK | Pathogenic​ |
|  | c.1132G>A | p.Ala378Thr | VCV000016145 | GCK | Pathogenic​ |
|  | c.1031_1034dup | p.Lys346fs | VCV000802307 | GCK | Pathogenic​ |
|  | c.1016A>G | p.Glu339Gly | VCV000393450 | GCK | Pathogenic​ |
|  | c.1015G>A | p.Glu339Lys | VCV000039759 | GCK | Pathogenic​ |
|  | c.1003del | p.Val335fs | VCV000036166 | GCK | Pathogenic​ |
|  | c.944T>A | p.Leu315His | VCV000036266 | GCK | Pathogenic​ |
|  | c.941T>C | p.Leu314Pro | VCV000435305 | GCK | Pathogenic​ |
|  | c.835G>T | p.Glu279Ter | VCV000016132 | GCK | Pathogenic​ |
|  | c.793G>T | p.Glu265Ter | VCV000016139 | GCK | Pathogenic​ |
|  | c.781G>A | p.Gly261Arg | VCV000016135 | GCK | Pathogenic​ |
|  | c.775G>A | p.Ala259Thr | VCV000435302 | GCK | Pathogenic​ |
|  | c.683C>T | p.Thr228Met | VCV000016134 | GCK | Pathogenic​ |
|  | c.680-1G>A |  | VCV000393452 | GCK | Pathogenic​ |
|  | c.678_679+2del |  | VCV000435308 | GCK | Pathogenic​ |
|  | c.676G>A | p.Val226Met | VCV000036243 | GCK | Pathogenic​ |
|  | c.661G>A | p.Glu221Lys | VCV000036241 | GCK | Pathogenic |
|  | c.645C>G | p.Tyr215Ter | VCV000036238 | GCK | Pathogenic |
|  | c.571C>T | p.Arg191Trp | VCV000426122 | GCK | Pathogenic |
|  | c.449T>C | p.Phe150Ser | VCV000036218 | GCK | Pathogenic |
|  | c.391T>C | p.Ser131Pro | VCV000016138 | GCK | Pathogenic |
|  | c.317_333del | p.Gln106fs | VCV000211071 | GCK | Pathogenic |
|  | c.295del | p.Trp99fs | VCV000435304 | GCK | Pathogenic |
|  | c.291del | p.Gln98fs | VCV000590262 | GCK | Pathogenic |
|  | c.214G>A | p.Gly72Arg | VCV000036209 | GCK | Pathogenic |
|  | c.184G>A | p.Val62Met | VCV000419624 | GCK | Pathogenic |
|  | c.183C>A | p.Tyr61Ter | VCV000802309 | GCK | Pathogenic |
|  | c.148C>T | p.His50Tyr | VCV000631495 | GCK | Pathogenic |
|  | c.106C>T | p.Arg36Trp | VCV000431973 | GCK | Pathogenic |
|  | c.45+1G>T |  | VCV000619964 | GCK | Pathogenic |
|  | GCK, IVS4DS, 15-BP DEL |  | VCV000016137.1 | GCK | Pathogenic |
|  | c.130del | p.Leu44fs | VCV000036798 | HNF1a | Pathogenic​ |
|  | c.335C>T | p.Pro112Leu | VCV000014942 | HNF1a | Pathogenic​ |
|  | c.365A>G | p.Tyr122Cys | VCV000014930 | HNF1a | Pathogenic​ |
|  | c.476G>A | p.Arg159Gln | VCV000586792 | HNF1a | Pathogenic​ |
|  | c.694dup | p.Leu232fs | VCV000393456 | HNF1a | Pathogenic​ |
|  | c.714-1G>A |  | VCV000014941 | HNF1a | Pathogenic​ |
|  | c.815G>A | p.Arg272His | VCV000014931 | HNF1a | Pathogenic​ |
|  | c.864_897del | p.Pro290fs | VCV000585219 | HNF1a | Pathogenic​ |
|  | c.956-1G>C |  | VCV000617646 | HNF1a | Pathogenic​ |
|  | c.1328_1329CA | p.Gln444fs | VCV000617650 | HNF1a | Pathogenic​ |
|  | c.1359del | p.Ser454fs | VCV000435427.1 | HNF1a | Pathogenic​ |
|  | c.1592G>C | p.Ser531Thr | VCV000014947.1 | HNF1a | Pathogenic​ |
|  | c.1747C>G | p.Arg583Gly | VCV000014932 | HNF1a | Pathogenic​ |
|  | c.*3039dup |  | VCV000438709 | HNF1a | Pathogenic​ |
|  | c.*2998G>A |  | VCV000014935 | HNF1a | Pathogenic​ |
|  | c.331C>T | p.Gln111Ter | VCV000009212 | HNF1a | Pathogenic​ |
|  | HNF1A, 2-BP DEL, AG |  | VCV000014946.1 | HNF1a | Pathogenic​ |
|  | HNF1A, 4-BP DEL |  | VCV000014944.1 | HNF1a | Pathogenic​ |
|  | HNF1A, 1-BP DEL, -119G, PROMOTER |  | VCV000014936.1 | HNF1a | Pathogenic​ |
|  | HNF1A, A-C, -58, PROMOTER |  | VCV000014933 | HNF1a | Pathogenic​ |
|  | HNF1A, 1-BP DEL |  | VCV000014929 | HNF1a | Pathogenic​ |
|  | c.253C>T | p.Arg85Trp | VCV000156152 | HNF4a | Pathogenic |
|  | c.331C>T | p.Gln111Ter | [VCV000617653](https://www.ncbi.nlm.nih.gov/clinvar/variation/VCV000617653) | HNF4a | Pathogenic |
|  | c.487C>T | p.Arg163Ter | VCV000009211 | HNF4a | Pathogenic |
|  | c.493-1G>A |  | VCV000587398 | HNF4a | Pathogenic |
|  | c.648+1G>A |  | VCV000617652 | HNF4a | Pathogenic |
|  | c.649-2del |  | VCV000009215 | HNF4a | Pathogenic |
|  | c.829C>T | p.Gln277Ter | VCV000009210 | HNF4a | Pathogenic |
|  | c.1118T>G | p.Met373Arg | VCV000009216 | HNF4a | Pathogenic |
|  | HNF4A, 1-BP DEL, PHE75T |  | VCV000009214.1 | HNF4a | Pathogenic |
|  | c.137G>A | p.Arg46Gln | VCV000013391 | INS | Pathogenic |
|  | c.16C>T | p.Arg6Cys | [VCV000013390](https://www.ncbi.nlm.nih.gov/clinvar/variation/VCV000013390) | INS | Pathogenic |
|  | c.533A>G | p.Glu178Gly | VCV000030124 | PDX1 | Pathogenic |
|  | PDX1, 1-BP DEL,188C |  | VCV000008857.1 | PDX1 | Pathogenic |
|  | c.309C>G | p.Arg103 | VCV000218145 | NEUROD1 | Pathogenic |
|  | c.772-1G>A |  | VCV000013794 | PAX4 | Pathogenic |
|  | c.514C>T | p.Arg172Trp | VCV000013793.1 | PAX4 | Pathogenic |
|  | c.*505G>T |  | VCV000059736 | BLK | Pathogenic |
|  | c.967dup | p.Asp323fs | VCV000478917 | KCNJ11 | Pathogenic |
|  | c.124T>C | p.Cys42Arg | VCV000008676 | KCNJ11 | Pathogenic |
|  | c.679G>A | p.Glu227Lys | VCV000158682 | KCNJ11 | Pathogenic |
|  | c.1039G>T | p.Ala347Ser | VCV000006498 | KLF11 | Pathogenic |
|  | CEL, 1-BP DEL, 1686T |  | VCV000017600.1 | CEL | Pathogenic |
